# Impact of anti-fracture medications on bone material and strength properties: a systematic review and meta-analysis

**DOI:** 10.3389/fendo.2024.1426490

**Published:** 2024-08-27

**Authors:** Shivani Sharma, Vijay Shankar, Singh Rajender, Ambrish Mithal, Sudhaker D. Rao, Naibedya Chattopadhyay

**Affiliations:** ^1^ Division of Endocrinology and Centre for Research in ASTHI, CSIR-Central Drug Research Institute, Council of Scientific and Industrial Research, Lucknow, India; ^2^ Academy of Scientific and Innovative Research (AcSIR), Ghaziabad, India; ^3^ Institute of Endocrinology and Diabetes, Max Healthcare, New Delhi, India; ^4^ Division of Endocrinology Diabetes and Bone & Mineral Disorders, and Bone and Mineral Research Laboratory, Henry Ford Health/Michigan State University College of Human Medicine, Detroit, MI, United States

**Keywords:** bone-quality, bone-strength, osteoporosis, bisphosphonate, denosumab, raloxifene, teriparatide, strontium-ranelate

## Abstract

**Background and aims:**

Reduced bone mineral density (BMD) and microarchitectural deterioration contribute to increased fracture risk. Although the effects of anti-fracture medications (AFMs) on BMD are well-documented, their impact on bone material properties (BMPs) remains poorly characterized. Accordingly, we conducted a systematic review and meta-analysis to evaluate the effects of AFMs on BMPs. Based on data availability, we further categorized AFMs into anti-resorptives, bisphosphonates alone, and strontium ranelate subgroups to perform additional analyses of BMPs in osteoporotic patients.

**Methods:**

We did a comprehensive search of three databases, namely, PubMed, Web of Science, and Google Scholar, using various permutation combinations, and used Comprehensive Meta-Analysis software to analyze the extracted data.

**Results:**

The 15 eligible studies (randomized and non-randomized) compared the following: (1) 301 AFM-treated patients with 225 on placebo; (2) 191 patients treated with anti-resorptives with 131 on placebo; (3) 86 bisphosphonate-treated patients with 66 on placebo; and (4) 84 strontium ranelate-treated patients with 70 on placebo. Pooled analysis showed that AFMs significantly decreased cortical bone crystallinity [standardized difference in means (SDM) −1.394] and collagen maturity [SDM −0.855], and collagen maturity in cancellous bone [SDM −0.631]. Additionally, anti-resorptives (bisphosphonates and denosumab) significantly increased crystallinity [SDM 0.387], mineral–matrix ratio [SDM 0.771], microhardness [SDM 0.858], and contact hardness [SDM 0.952] of cortical bone. Anti-resorptives increased mineral–matrix ratio [SDM 0.543] and microhardness [SDM 0.864] and decreased collagen maturity [SDM −0.539] in cancellous bone. Restricted analysis of only bisphosphonate-treated studies showed a significant decrease in collagen maturity [SDM −0.650] in cancellous bone and an increase in true hardness [SDM 1.277] in cortical bone. In strontium ranelate-treated patients, there was no difference in BMPs compared to placebo.

**Conclusion:**

Collectively, our study suggests that AFMs improve bone quality, which explains their anti-fracture ability that is not fully accounted for by increased BMD in osteoporosis patients.

## Introduction

Osteoporosis, primarily age- and menopause-related, leads to declining bone strength in older individuals. Areal bone mineral density (aBMD) is a standard measure for diagnosis, but it only accounts for 30%–40% of bone strength variation (1, 2). Other factors like bone geometry, microarchitecture, mineralization, collagen integrity, and biomaterial composition also impact bone quality (2–4). Assessing these parameters aids in fracture risk identification and developing better bone health strategies.

Bone health in adults is maintained by a continuous process of bone remodeling, in which old damaged bone is resorbed by osteoclasts and subsequently replaced by new bone by osteoblasts. In healthy individuals, this remodeling process is balanced, with the amount of bone resorbed is replaced by almost an equal amount of new bone, ensuring the maintenance of bone strength and integrity. In osteoporosis, the bone remodeling rate is increased. Since the bone resorption phase that precedes the bone formation phase has a much shorter duration, increases in the remodeling rate lead to net bone loss (5, 6). In this regard, anti-resorptives, the drugs that suppress bone remodeling including bisphosphonates (BPs), neutralizing antibody against RANKL [denosumab (DMab)], and raloxifene (Ral), a selective estrogen receptor modulator (SERM), prevent bone loss over time and increase BMD (7, 8). As bone remodeling is a physiological process, inhibiting it could potentially affect the quality of the bone matrix.

Besides increases in bone resorption, osteoporosis is also marked by an impairment in new bone formation (9). In this context, osteo-anabolic drugs, including teriparatide (TPTD) and abaloparatide, signaling through the type 1 PTH receptor, increase BMD by stimulating osteoblast number and function without affecting the function of osteoclasts (9, 10). TPTD and abaloparatide maintain a higher rate of bone remodeling in which there is a time window when formation exceeds resorption, lasting approximately 2 years, often referred to as “anabolic window” (11). Romosozumab, a neutralizing antibody against sclerostin, although believed to have a dual action of promoting bone formation and inhibiting resorption, displayed a limited period when bone formation exceeds resorption (12, 13). Strontium ranelate (SR) showed dual action in preclinical studies, but it did not show significant osteoanabolic effect in clinical trials (14). Considering that remodeling–suppressing drugs and osteoanabolic drugs have two distinct mechanisms of action and target different aspects of bone remodeling, they can lead to variations in bone material composition, matrix properties, and strength.

To understand the effects of anti-fracture medications (AFMs) on bone material and strength, we conducted a systematic review and meta-analyses by comparing outcomes in osteoporosis patients on medication with those on a placebo. We then classified the AFM groups according to their modes of action (anti-resorptive and anabolic) and investigated their effects on bone material and strength measures. Various measurement techniques, including Raman spectroscopy (RS), Fourier transform infrared imaging (FTIRI), quantitative back-scattered electron imaging (qBEI), quantitative micro-radiography (QMR), x-ray diffraction (XRD), and nanoindentation, were employed to assess bone composition and mechanical properties. RS and FTIRI determine the chemical composition of bone, including collagen maturity, the mean degree of mineralization, and carbonate/phosphate and carbonate/amide-I ratios (15, 16). qBEI and QMR quantified mineral content and distribution within bone tissue, revealing mineralization state and density. XRD examined bone mineral crystal structure, assessing crystallinity and orientation (17). Nanoindentation and microindentation (Vickers indentation), which measure dynamic and static mechanical properties, such as hardness and elasticity, offer insights into bone strength and resilience (18). By analyzing this evidence, we sought to determine the effectiveness of various AFMs in improving bone quality and strength properties in osteoporosis patients.

## Methods

### Search strategy and inclusion criteria

**Figure 1:**
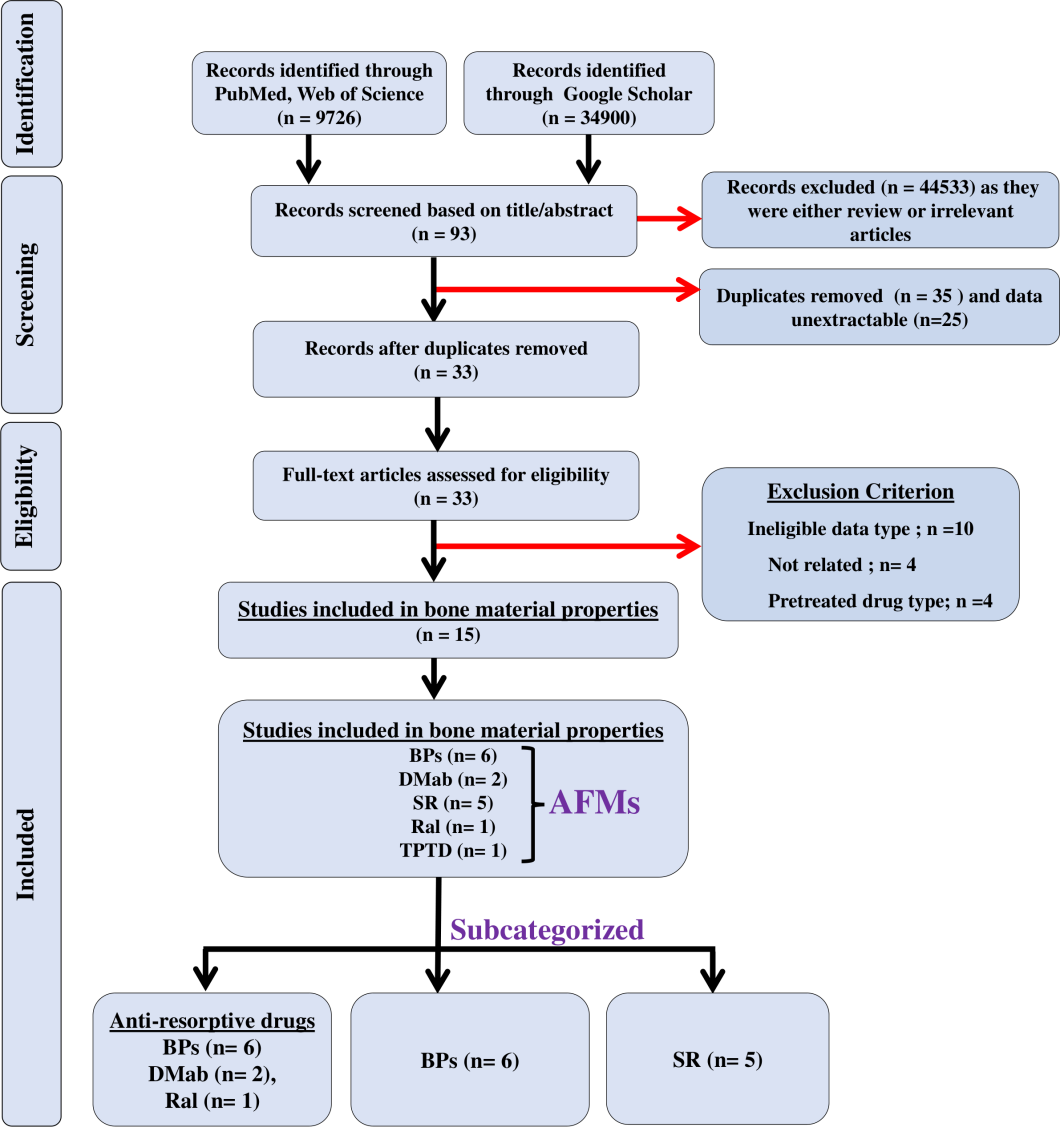
The flow of information is shown in a PRISMA diagram. *n* = the number of studies; black arrows indicate studies that were included in the meta-analyses; red arrows indicate excluded studies.

We searched three electronic databases, namely, PubMed, Web of Science, and Google Scholar, until 20 July 2024, to identify the studies that assessed the effect of AFM on bone quality, and articles were exported to Endnote. The search strategy was a combination of keywords and Boolean operators and was limited to original articles published in the English language (**Supplementary Table 1**). The search terms included: (“bisphosphonates” OR “alendronate” OR “residronate” OR “zoledronic acid” OR “ibandronate” OR “etidronate” OR “denosumab” OR “teriparatide” OR “abaloparatide” OR “raloxifene” OR “romosozumab” OR “strontium ranelate”), (“human” OR “men” OR “women”), (“bone quality” OR “mineral matrix ratio” OR “cross-linking” OR “carbonate phosphate ratio” OR “crystallinity” OR “water content” OR “heterogeneity index” OR “heterogeneity” OR “microdamage” OR “microdamage accumulation” OR “microdamage propagation”), (“Raman spectroscopy” OR “Fourier transform infrared imaging” OR “quantitative back-scattered electron imaging” OR “quantitative micro-radiography” OR “X-ray diffraction, OR “nanoindentation”), (“osteoporosis” OR “post-menopausal osteoporosis” OR “age-related Osteoporosis” OR “chronic kidney disease” OR “glucocorticoid-induced osteoporosis” OR “hypertension” OR “inflammatory bowel disease” OR “diabetes” OR “arthritis”). The PRISMA flowchart depicts the findings of the literature search (**Figure 1**).

### Inclusion and exclusion criteria

The literature search results were screened based on inclusion/exclusion criteria. Inclusion criteria encompassed original full-text articles related to osteoporosis from various causes [postmenopausal, aging, chronic kidney disease (CKD), glucocorticoid-induced osteoporosis (GIO), hypertension, inflammatory bowel disease (IBD), diabetes, and arthritis]. Exclusion criteria included review articles, case reports, book chapters, letters, editorials, and conference proceedings. No restrictions were imposed on age, gender, or treatment duration.

### Data extraction

Three authors (S.S., V.S., and N.C.) independently screened titles, abstracts, and full texts to confirm the eligibility of each study and compiled an Excel sheet (Windows 10 edition; Microsoft Corporation, Lisbon, Portugal) depicting the following parameters: author name, year of publication, techniques used, gender, age, and bone-related parameters. Data from each article were retrieved in numeric form from the tables and bar graphs using the WebPlotDigitilizer program.

### Quality and outcome assessment

Study quality was assessed through a checklist, including criteria such as the inclusion of osteoporosis patients, specific treatments, trial type, randomization, informed consent, ethics approval, and conflict of interest statements. We considered bone material and strength as primary outcome measures in response to AFM.

### Quantitative data analysis

All statistical analyses in this study were conducted using Comprehensive Meta-Analysis Software (CMA) Version 2. Cochran’s *Q*-test and heterogeneity index (*I* ^2^) were used to determine the degree of heterogeneity across the studies. The *I* ^2^ value ranges from 0% to 100%. High heterogeneity is present when *I* ^2^ value is >75%, moderate heterogeneity is present when *I* ^2^ value is 50%, and low heterogeneity is present when *I* ^2^ value is <25%. We appropriately applied the fixed- and random-effect models to calculate the pooled effect size, as previously described (19). The fixed-effect model assumes that different studies estimate the same effect, whereas the random-effect model assumes that different studies estimate different effects (19).

### Sensitivity analysis and publication bias

The level of sensitivity for each bone quality parameter was determined by a single study exclusion statistic using the CMA software as described previously (19). We examined publication bias qualitatively using the funnel plot and quantitatively using Egger’s regression intercept test (20), Begg and Mazumdar rank correlation test (21), and Duval and Tweedie’s trim-and-fill method (22).

### Software used

Data from each article were extracted and manually entered in numeric form. The data were presented in a Microsoft Excel spreadsheet (Windows 10 edition; Microsoft Corporation, Lisbon, Portugal) to record treatment, dose, treatment duration, study design, gender, disease, sample size, number of patients, age, and techniques used for quantifying bone quality. For the current study, references were managed using Endnote X7 (Thomson Reuters, Toronto, Canada), and data were analyzed using Comprehensive Meta-Analysis software.

## Results

### Study design and parameters measured

A literature search for the effect of AFM identified 44,626 articles: PubMed and Web of Science (*n* = 9,726), and Google Scholar (*n* = 34,900). Of these, 44,533 studies were excluded for being either reviews or not relevant and the remaining 93 were eligible after screening for the titles and abstracts. Of these, 35 studies were duplicates and the data were unextractable in 25 studies, leaving 33 studies that underwent full-text assessment. Of the 33 studies, 18 were excluded due to the following reasons: (a) patients who received a prior AFM different from the one being studied for BMPs, to avoid confounding effects and ensure accurate interpretation of outcomes; (b) no control group; and (c) data were not presented as mean ± SD/SEM. The remaining 15 studies of AFM were eligible for meta-analyses. Of these studies, six used BPs, two used DMab, five used SR, one used TPTD, and one used Ral (**Figure 1**); all studies were published from 2003 to 2022 (16, 23–36). Patient details are provided in **Table 1**.

**Table 1:** Characteristics of studies included for meta-analyses.

Sr.no.	Author, year	Drug	Study design	Gender/disease type	Age (years) (placebo)	Age (years) (treated)	Sample	Site	Dose	Duration (years)	N (placebo)	N (treated)
**1**	**Boivin G et al., 2003** (23)	RAL	Placebo-controlled, double-blind, multicenter trial	F/PMO	68 ± 5	67 ± 8 to 69 ± 5	Iliac crest bone biopsies	Total, cortical, cancellous bone	60 mg/day and 120 mg/day	2	24	60 mg/day: 22 120 mg/day: 18
**2**	**Paschalis EP et al., 2005** (29)	TPTD	Randomized, placebo trail	F/PMO	68 ± 4	69 ± 5	Transiliac crest bone biopsy	Cortical and cancellous bone	20 μg	1–2	12	13
					68 ± 4	67 ± 8			40 μg		12	13
**3**	**Durchschlag E et al., 2006** (30)	BPs	Double blind, placebo-controlled trial	F/PMO	66 ± 9 to 69 ± 9	66 ± 7 to 70 ± 7	Iliac crest biopsies	Cancellous bone	5 mg/day	3	8	10
**4**	**Boskey AL et al., 2009** (16)	BPs	Retrospective data, placebo double-blind, RCT	F/PMO	51 ± 1	52 ± 1	Transiliac bone biopsies	Cortical and cancellous bone	10 mg/day	3	10	7
**5**	**Li C et al., 2010** (31)	SR	Prospective, randomized, double-blind, placebo-controlled trial	F/PMO	69 ± 7 to 77 ± 5	69 ± 7 to 77 ± 5	Iliac core biopsy	Cancellous bone	2 g/day	3	5	5
**6**	**Boivin G et al., 2010** (32)	SR	Randomized, double-blind, placebo-controlled clinical trials	F/PMO	67 ± 7 to 77 ± 5	66 ± 7 to 77 ± 5	Iliac core biopsy	Total, cortical, cancellous bone	0.5, 1, 2 g/day	2 and 3	2 years = 7 3 years = 15	**2 years:** 0.5 g/day: 6 1 g/day: 6 2 g/day: 8 **3 years:** 2 g/day: 15
**7**	**Roschger P et al., 2010** (33)	SR	Placebo-controlled trial	F/PMO	69 ± 7 to 77 ± 5	69 ± 7 to 77 ± 5	Iliac core biopsy	Cancellous bone	2 g/day	3	6	6
**8**	**Doublier A et al., 2011** (34)	SR	Randomized, double-blind, placebo-controlled clinical trials	F/PMO	72 ± 5	72 ± 5	Iliac core biopsy	Total (cortical cancellous) bone	2 g/day	5	36M: 21 48M: 6 60M: 7	36M: 17 48M: 7 60M: 7
**9**	**Tjhia CK et al., 2011** (35)	BPs	Placebo-controlled trial	M/F (aged)	66 ± 8	64 ± 9	Transiliac core biopsy	Cortical and cancellous bone	ALN: 10 mg, RIS: 5mg	Minimum of 3	11	12
**10**	**Doublier A et al., 2013** (28)	SR	Randomized, double-blind, placebo-controlled clinical trials SOTI and TROPOS	F/PMO	75 ± 4	75 ± 4	Paired transiliac bone biopsies	Cortical and cancellous bone	2 g/day	3	3	7
**11**	**Bala Y et al., 2012** (36)	BPs	Placebo, cross-sectional design	F/PMO	69 ± 2	72 ± 8	Transiliac bone biopsies	Cortical bone	10 mg daily or 70 mg weekly	8 ± 2	5	6
**12**	**Donnelly E et al., 2012** (24)	BPs	Placebo, blinded fashion	F/PMO	87 ± 6	80 ± 11 to 75 ± 10	Iliac crest biopsies	Cortical and cancellous bone	NA	All: 7.0 ± 5 Typical fractures: 6.6 ± 5.1 Atypical fractures: 7.8 ± 2.6	20	20
**13**	**Bala Y et al., 2013** (25)	BPs	Multicenter, double-blind, placebo-controlled, randomized	F/PMO	66 ± 6	66 ± 6	Transiliac biopsy	Cortical and cancellous bone	2.5 mg/day or 20 mg intermittent	3	12	2.5 mg/day: 16 20 mg intermittent: 15
**14**	**Jähn-Rickert K et al., 2020** (26)	DMab	Treatment naïve	M/F (Aged)	59 ± 15	68 ± 11	Iliac core biopsy	Cortical bone	Two semiannual injections of 60 mg	Approximately 2	11	23
**15**	**Farlay D et al., 2022** (27)	DMab	Randomized, blinded fashion (cross-sectional data)	F	73 ± 6	75 ± 5	Iliac core biopsy	Cortical and cancellous bone	60 mg	2–3	30	42

BPs: bisphosphonate, Ral: raloxifene, Sr: strontium ranelate, DMab: denosumab, TPTD: teriparatide, M: male, F: female, PMO, Postmenopausal osteoporotic; NA, not available, FTIRI: Fourier transform infrared, SAXS/XRD: small-angle x-ray scattering vs. x-ray diffraction.

We first pooled data across all AFMs to study their combined effects on BMPs. We then categorized AFMs by their mode of action (anti-resorptive vs. anabolic) and investigated their effects on BMPs. Adequate data on anti-resorptive drugs (BPs, DMab, and Ral) allowed us to form this category and analyze their impact on bone material and strength. Subgroup analyses of BPs and SR were possible due to sufficient datasets, comparing their effects to placebo in osteoporotic patients. However, insufficient data for DMab, Ral, and the anabolic agent teriparatide prevented individual assessments of these treatments.

#### Effect of pooled AFM compared to placebo

##### Degree of mineralization of bone

QMR and XRD measured degree of mineralization of bone (DMB), representing the amount of mineral in a unit volume of tissue matrix (37, 38). Pooled data from three studies (nine datasets) for a total skeleton, standardized difference in means [(SDM) = 0.230, 95% CI = −0.038 to 0.498, *p* = 0.093, *I* ^2 =^ 0.000; *Q* = 3.326, *p* = 0.912], four studies (11 datasets) for cortical bones (SDM = 0.206, 95% CI = −0.033 to 0.445, *p* = 0.091, *I* ^2 =^ 0.000; *Q* = 3.033, *p* = 0.981), and three studies (10 datasets) for cancellous bones (SDM = 0.196, 95% CI = −0.049 to 0.441, *p* = 0.117, *I* ^2 =^ 0.000; *Q* = 7.172, *p* = 0.619) (**Supplementary Figures 1A–C**) revealed that AFM did not affect DMB (**Table 2**).

**Table 2:** Summary of analyzed pooled data from all AFM of various parameters.

Parameters	Sites	Test of heterogeneity			Test model	Type of association				Level of significance
							95% CI			
		*Q*	*p*-value	*I* ^2^		SDM	Lower limit	Upper limit	*p*-value	
**DMB**	Total	3.326	0.912	0.000	Fixed	0.230	−0.038	0.498	0.093	Non-significant
	Cortical	3.033	0.981	0.000	Fixed	0.206	−0.033	0.445	0.091	Non-significant
	Cancellous	7.172	0.619	0.000	Fixed	0.196	−0.049	0.441	0.117	Non-significant
**HI**	Cortical	11.255	0.188	28.922	Fixed	0.020	−0.274	0.313	0.896	Non-significant
	Cancellous	24.067	0.001	70.914	Random	0.434	−0.163	1.030	0.154	Non-significant
**XST**	**Cortical**	**120.808**	**0.000**	**92.550**	**Random**	**−1.394**	**−2.525**	**−0.263**	**0.016**	**Significant**
	Cancellous	46.634	0.000	87.134	Random	−0.902	−1.837	0.034	0.059	Non-significant
**MMTR**	Cortical	125.177	0.000	95.207	Random	−1.304	−2.815	0.207	0.091	Non-significant
	Cancellous	53.656	0.000	92.545	Random	−0.668	−1.931	0.596	0.300	Non-significant
**C/P**	Cortical	1.935	0.380	0.000	Fixed	−0.093	−0.443	0.257	0.603	Non-significant
	Cancellous	7.254	0.027	72.428	Random	−0.255	−0.998	0.487	0.500	Non-significant
**XLR**	**Cortical**	**33.241**	**0.000**	**78.942**	**Random**	**−0.855**	**−1.481**	**−0.229**	**0.007**	**Significant**
	**Cancellous**	**7.980**	**0.240**	**24.815**	**Fixed**	**−0.631**	**−0.913**	**−0.348**	**0.000**	**Significant**

AFM, anti-fracture medication; DMB, degree of mineralization; HI, heterogeneity index; XST, mineral crystallinity; MMTR, mineral–matrix ratio; C/P, carbonate-to-phosphate ratio; XRL, collagen maturity ratio. Significant parameters are presented in bold.

##### Heterogeneity index

Heterogeneity index (HI) is the heterogeneity of the distribution of the DMB, calculated as the width at half-maximum of the curve quantified by QMR (32, 34). Pooled data from three studies (nine datasets) for cortical bones (SDM = 0.020, 95% CI = −0.274 to 0.313, *p* = 0.896, *I* ^2 =^ 28.920; *Q* = 11.255, *p* = 0.188) and two studies (eight datasets) for cancellous bones (SDM = 0.434, 95% CI = −0.163 to 1.030, *p* = 0.154, *I* ^2 =^ 70.914; *Q* = 24.067, *p* = 0.001) (**Supplementary Figures 2A, B**) revealed that AFM had no significant impact on HI (**Table 2**).

##### Mineral crystallinity

**Figure 2:**
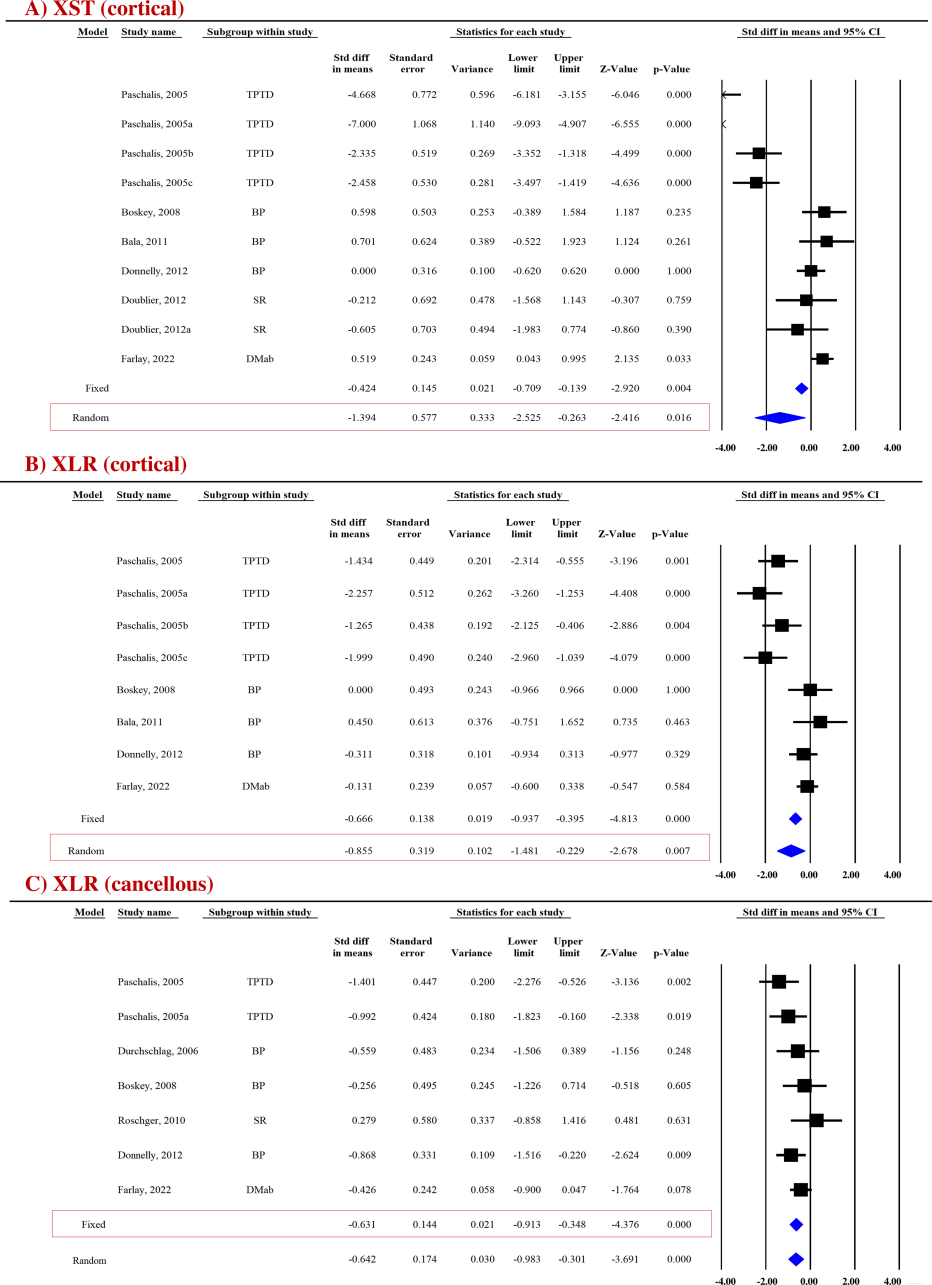
Forest plot showing the impact of AFM on **(A)** XST and **(B, C)** XLR at the indicated sites. The *Z*-value denotes the strength and degree of the relationship, and the *p*-value indicates the significance of outcomes. A fixed- or random-effect model was used, as indicated in the red box.

FTIR-based measurement of crystallinity reflects the size and shape of hydroxyapatite crystals. Appropriate packing of thin nanocrystals of carbonated apatite and calcium phosphate within the organic matrix is an important determinant of bone strength with smaller-sized crystals improving mechanical behavior (39). Pooled data were available from six studies (10 datasets) for cortical bones and five studies (7 datasets) for cancellous bones, which revealed that AFM significantly reduced mineral crystallinity (XST) in cortical (SDM = −1.394, 95% CI = −2.525 to −0.263, *p* = 0.016, *I* ^2 ^= 92.550; *Q* = 120.808, *p* = 0.000) (**Figure 2A**) and cancellous bones (SDM = −0.902, 95% CI = −1.837 to 0.034, *p* = 0.059, *I* ^2^ = 87.134; *Q* = 46.634, *p* = 0.000) (**Supplementary Figure 3A**) compared with placebo (**Table 2**).

##### Mineral-to-matrix ratio

FTIR-based measurement of mineral-to-matrix ratio (MMTR) represents the amount of mineral ion substitution in the collagenous matrix in the bone tissue and is a determinant of bone fragility and strength (40). Data from four studies provided a total of seven datasets at the cortical site and five datasets at the cancellous site. Analysis indicated no significant difference in MMTR between patients who received AFM and those on a placebo, in both cortical (SDM = −1.304, 95% CI = −2.815 to 0.207 and, *p* = 0.091, *I* ^2 =^ 95.207; *Q* = 125.177, *p* = 0.000) (**Supplementary Figure 3B**) and cancellous bones (SDM = −0.668, 95% CI = −1.931 to 0.596, *p* = 0.300, *I* ^2 =^ 92.545; *Q* = 53.656, *p* = 0.000) (**Supplementary Figure 3C**; **Table 2**).

##### Carbonate/phosphate ratio

FTIR-based measurement of carbonate/phosphate ratio (C/P) represents how much PO_4_^3−^ ion in the organic matrix is exchanged by a CO_3_^2−^ ion (41). Data from three studies provided as many datasets for cortical (SDM = −0.093, 95% CI = −0.443 to 0.257, *p* = 0.603, *I* ^2^ = 0.000; *Q* = 1.935, *p* = 0.380) (**Supplementary Figure 4A**) and cancellous bones (SDM = −0.255, 95% CI = −0.998 to 0.487, *p* = 0.500, *I* ^2^ = 72.428; *Q* = 7.254, *p* = 0.027) (**Supplementary Figure 4B**), revealing no effect of AFM on the C/P ratio (**Table 2**).

##### Collagen maturity ratio

FTIR and RS techniques were used to evaluate collagen maturity ratio (XLR), which indicates the proportion of mature to immature cross-links and is influenced by both age and bone turnover (42). Eight datasets from five studies and seven datasets from six studies were available for pooled analysis for cortical (**Figure 2B**) and cancellous bones (**Figure 2C**), respectively. AFM reduced XLR in cortical (SDM = −0.855, 95% CI = −1.481 to −0.229, *p* = 0.007, *I* ^2^ = 78.942; *Q* = 33.241, *p* = 0.000) and cancellous bones (SDM = −0.631, 95% CI = −0.913 to −0.348, *p* = 0.000, *I* ^2^ = 24.815; *Q* = 7.980, *p* = 0.240) compared with placebo (**Table 2**).

#### Effect of pooled anti-resorptive drugs compared to placebo

##### DMB

Pooled data from three studies (seven datasets) for cortical bones (SDM = 0.208, 95% CI = −0.066 to 0.482, *p* = 0.136, *I* ^2^ = 0.000; *Q* = 1.055, *p* = 0.983) (**Supplementary Figure 5A**) and two studies (six datasets) for cancellous bones (SDM = 0.165, 95% CI = −0.117 to 0.448, *p* = 0.252, *I* ^2^ = 0.000; *Q* = 4.806, *p* = 0.440) (**Supplementary Figure 5B**) revealed that anti-resorptive drugs (BPs and Ral) did not affect DMB (**Table 3**).

**Table 3:** Summary of analyzed pooled data from anti-resorptive drugs of various parameters.

Parameters	Sites	Test of heterogeneity			Test model	Type of association				Level of significance
							95% CI			
		*Q*	*p*-value	*I* ^2^		SDM	Lower limit	Upper limit	*p*-value	
**DMB**	Cortical	1.055	0.983	0.000	Fixed	0.208	−0.066	0.482	0.136	Non-significant
	Cancellous	4.806	0.440	0.000	Fixed	0.165	−0.117	0.448	0.252	Non-significant
**XST**	**Cortical**	**2.220**	**0.528**	**0.000**	**Fixed**	**0.387**	**0.048**	**0.726**	**0.025**	**Significant**
	Cancellous	4.280	0.118	53.275	Random	−0.140	−0.698	0.417	0.622	Non-significant
**XLR**	Cortical	1.287	0.732	0.000	Fixed	−0.122	−0.457	0.214	0.477	Non-significant
	**Cancellous**	**1.535**	**0.674**	**0.000**	**Fixed**	**−0.539**	**−0.872**	**−0.206**	**0.002**	**Significant**
**MMTR**	**Cortical**	**3.864**	**0.145**	**48.245**	**Fixed**	**0.771**	**0.407**	**1.134**	**0.000**	**Significant**
	**Cancellous**	**0.477**	**0.788**	**0.000**	**Fixed**	**0.543**	**0.188**	**0.899**	**0.003**	**Significant**
**MH**	**Cortical**	**2.652**	**0.618**	**0.000**	**Fixed**	**0.858**	**0.551**	**1.166**	**0.000**	**Significant**
	**Cancellous**	**15.301**	**0.004**	**73.858**	**Random**	**0.864**	**0.230**	**1.498**	**0.008**	**Significant**
**Hc**	**Cortical**	**9.005**	**0.029**	**66.684**	**Random**	**0.952**	**0.092**	**1.811**	**0.003**	**Significant**
**EM**	Cortical	4.093	0.252	26.708	Fixed	0.190	−0.258	0.637	0.406	Non-significant

DMB, degree of mineralization; XST, mineral crystallinity; XRL, collagen maturity ratio; MMTR, mineral–matrix ratio; MH, microhardness (kg/mm^2^); Hc, contact hardness (GPa); H, true hardness (GPa); EM, elastic modulus (GPa). Significant parameters are presented in bold.

##### XST

**Figure 3:**
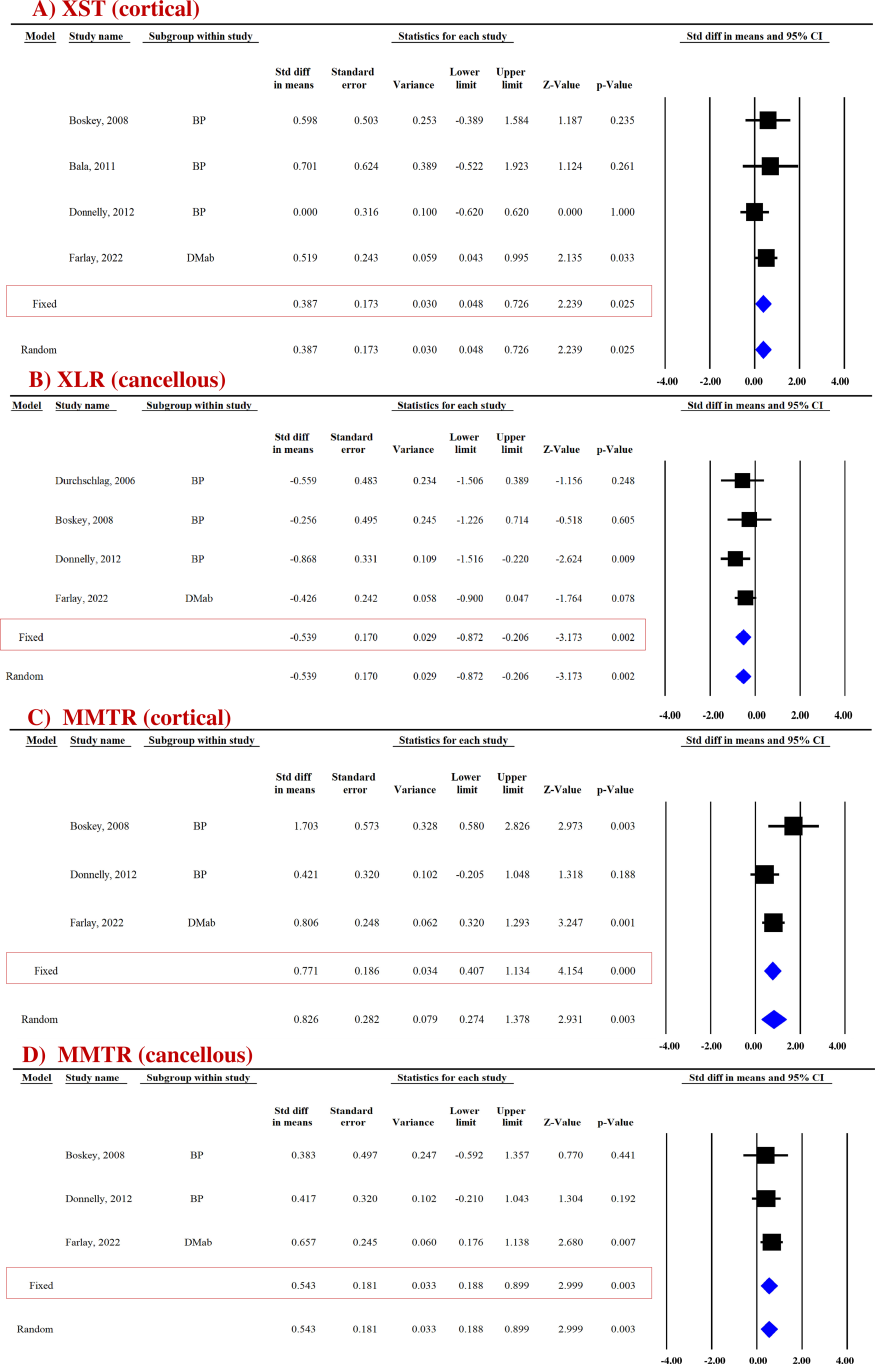
Forest plot showing the impact of anti-resorptive drugs on **(A)** XST, **(B)** XLR, and **(C, D)** MMTR at the indicated sites, compared with the placebo group. A fixed-effect model was used.

Pooled data from four studies (four datasets) for cortical bones and three studies (three datasets) for cancellous bones revealed that compared with the placebo group, the anti-resorptives (BPs and DMAb) significantly increased XST in the cortical (SDM = 0.387, 95% CI = 0.048 to 0.726, *p* = 0.025, *I* ^2^ = 0.000; *Q* = 2.220, *p* = 0.528) (**Figure 3A**) but not in the cancellous bone (SDM = −0.140, 95% CI = −0.698 to 0.417, *p* = 0.622, *I* ^2^ = 53.275; *Q* = 4.280, *p* = 0.118) (**Supplementary Figure 6A**) (**Table 3**).

##### XLR

Pooled data from four studies (four datasets) were available for cortical (**Supplementary Figure 6B**) and cancellous bones (**Figure 3B**). Anti-resorptive drugs (BPs and DMAb) decreased XLR in the cancellous bone (SDM = −0.539, 95% CI = −0.872 to −0.206, *p* = 0.002, *I* ^2^ = 0.000; *Q* = 1.535, *p* = 0.674) compared with placebo (**Figure 3B**), but had no effect in cortical bones (SDM = − 0.122, 95% CI = −0.457 to 0.214, *p* = 0.477, *I* ^2^ = 0.000; *Q* = 1.287, *p* = 0.732) (**Supplementary Figure 6B**) (**Table 3**).

##### MMTR

Pooled data from three studies (three datasets) were available for cortical (SDM = 0.771, 95% CI = 0.407 to 1.134, *p* = 0.000, *I* ^2^ = 48.245; *Q* = 3.864, *p* = 0.145) (**Figure 3C**) and cancellous (SDM = 0.543, 95% CI = 0.188 to 0.899, *p* = 0.003, *I* ^2^ = 0.000; *Q* = 0.477, *p* = 0.788) bones (**Figure 3D**). BPs and DMAb increased MMTR at both sites compared with the placebo group (**Table 3**).

##### Micro-hardness

**Figure 4:**
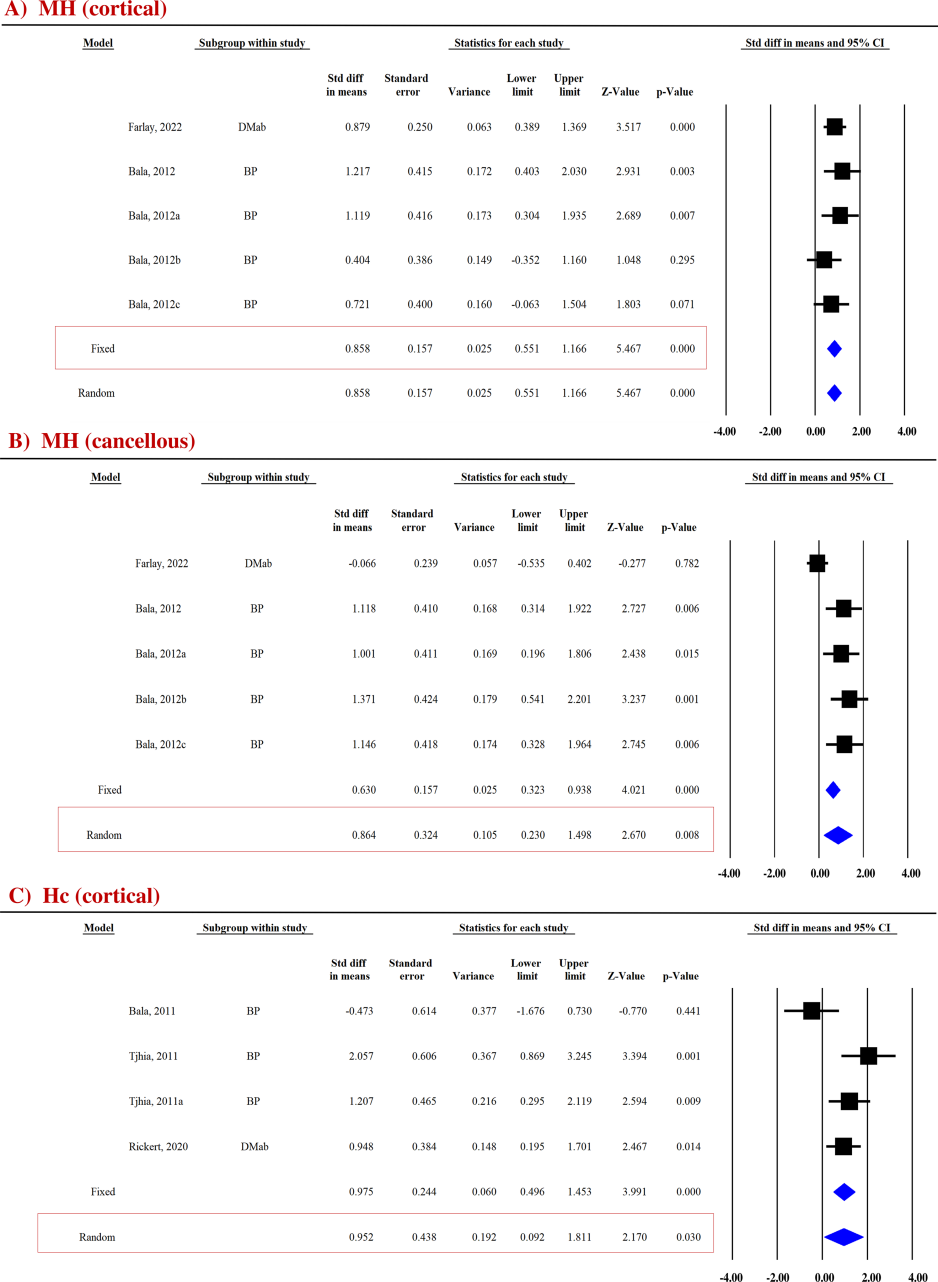
Forest plot showing the effect of anti-resorptive drugs on **(A, B)** MH and **(C)** Hc at indicated sites. A fixed- or random-effect model was used, as indicated in the red box.

Micro-hardness (MH) obtained by the microindentation technique (Vickers hardness test) is a measure of bone’s mechanical properties during deformation at the levels of the bone structural unit (43, 44). Pooled data from two studies (five datasets) were available for cortical (SDM = 0.858, 95% CI = 0.551 to 1.166, *p* = 0.000, *I* ^2^ = 0.000; *Q* = 2.652, *p* = 0.618) (**Figure 4A**) and cancellous bones (SDM = 0.864, 95% CI = 0.230 to 1.498, *p* = 0.008, *I* ^2^ = 73.858; *Q* = 15.301, *p* = 0.004) (**Figure 4B**). Anti-resorptive drugs (BPs and DMAb) significantly increased MH at both sites compared with placebo (**Table 3**).

##### Contact hardness

Bone hardness, encompassing both elastic and plastic deformation, was assessed using nanoindentation. Contact hardness (Hc) was calculated from both elastic and plastic properties using the Oliver–Pharr method (35, 36). For the cortical bones, pooled data from three studies (four datasets) for Hc (SDM = 0.952, 95% CI = 0.092 to 1.811, *p* = 0.030, *I* ^2^ = 66.684; *Q* = 9.005, *p* = 0.029) (**Figure 4C**) were available. Anti-resorptive drugs (BPs and DMAb) significantly increased Hc compared with placebo (**Table 3**).

##### Elastic modulus

Elastic modulus (EM) is the measure of elastic properties of bone material. EM is a function of the unloading stiffness of the force–displacement curve, as measured by the nanoindentation method (36). Pooled data from three studies (4 datasets) were available from cortical bone (SDM = 0.190, 95% CI = −0.258 to 0.637, *p* = 0.406, *I* ^2^ = 26.708; *Q* = 4.093, *p* = 0.252) that showed no significant effect of anti-resorptive drugs (BPs and DMAb) on EM (**Supplementary Figure 6C**; **Table 3**).

#### Effect of BPs

##### DMB and XST

Pooled analysis from two studies (five datasets) for cortical DMB (SDM = 0.205, 95% CI = −0.155 to 0.566, *p* = 0.265, *I^2 =^* 0.000; *Q*= 0.806, *p*= 0.938) (**Supplementary Figure 7A**; **Table 4**) and three studies (three datasets) for cortical XST (SDM = 0.252, 95% CI = −0.230 to 0.734, *p* = 0.306, *I* ^2^ = 0.000; *Q* = 1.624, *p* = 0.444) (**Supplementary Figure 7B**; **Table 4**) showed no significant difference between the BP-treated and placebo groups.

**Table 4:** Summary of analyzed pooled data from BPs of various parameters.

Parameters	Sites	Test of heterogeneity			Test model	Type of association				Level of significance
							95% CI			
		*Q*	*p*-value	*I* ^2^		SDM	Lower limit	Upper limit	*p*-value	
**DMB**	Cortical	0.806	0.938	0.000	Fixed	0.205	−0.155	0.566	0.265	Non-significant
**XST**	Cortical	1.624	0.444	0.000	Fixed	0.252	−0.230	0.734	0.306	Non-significant
**XLR**	Cortical	1.284	0.526	0.000	Fixed	−0.112	−0.593	0.368	0.646	Non-significant
	**Cancellous**	**3.697**	**0.448**	**0.000**	**Fixed**	**−0.650**	**−1.118**	**−0.181**	**0.007**	**Significant**
**Hc**	Cortical	8.997	0.011	77.769	Random	0.944	−0.399	2.286	0.168	Non-significant
**H**	**Cortical**	**7.322**	**0.026**	**72.684**	**Random**	**1.277**	**0.040**	**2.514**	**0.043**	**Significant**
**EM**	Cortical	2.350	0.309	14.890	Fixed	−0.043	−0.609	0.522	0.881	Non-significant

BPs, bisphosphonates; DMB, degree of mineralization; XST, mineral crystallinity; XRL, collagen maturity ratio; Hc, contact hardness (GPa); H, true hardness (GPa); EM, elastic modulus (GPa). Significant parameters are presented in bold.

##### XLR

**Figure 5:**
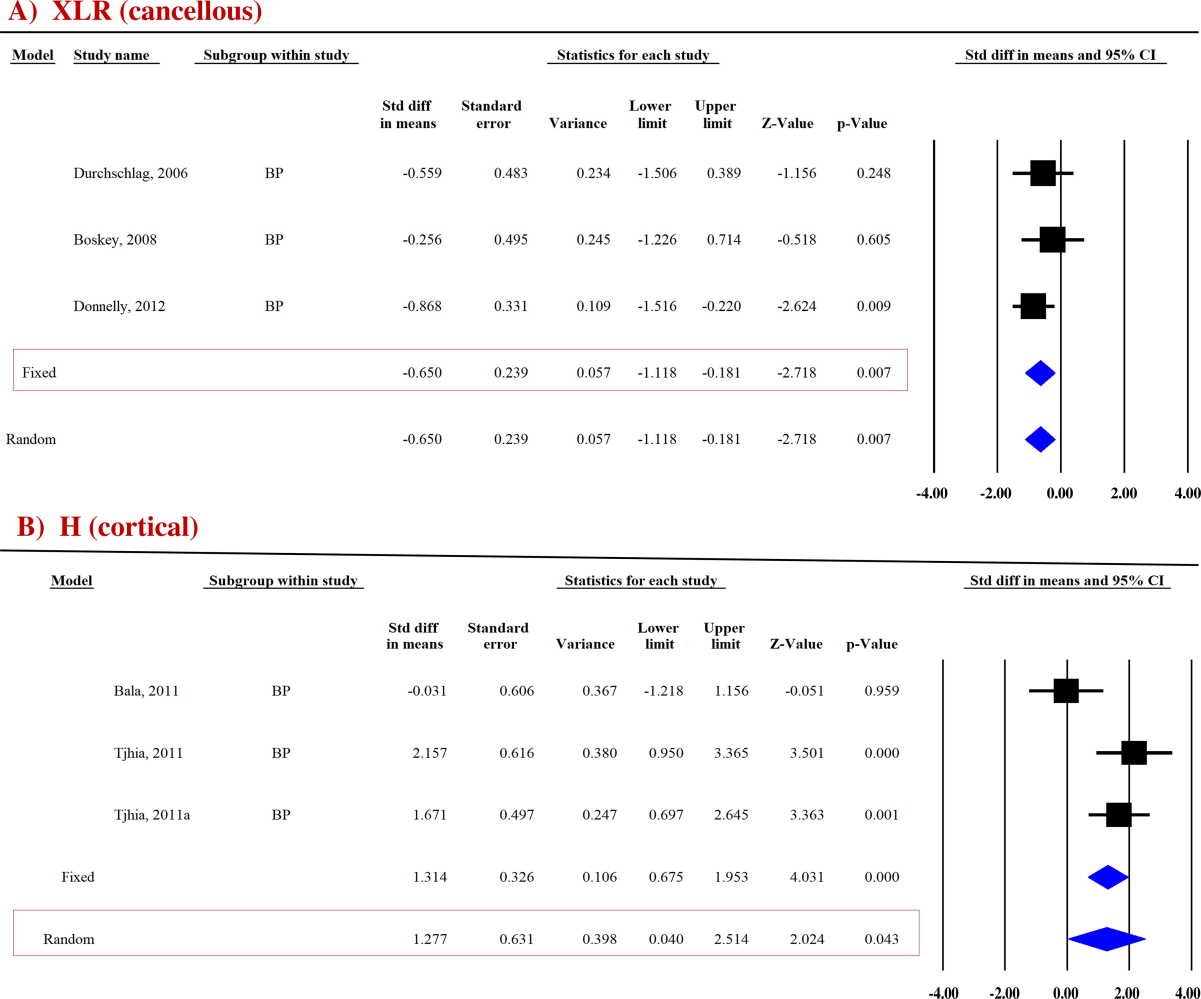
Forest plot showing the effect of BPs on **(A)** XLR and **(B)** H compared to placebo. A fixed- or random-effect model was used, as indicated by a red box.

Three datasets from as many studies were analyzed for cancellous and cortical bones. Pooled analysis showed that cancellous XLR was significantly reduced (SDM = −0.650, 95% CI = −1.118 to −0.181, *p* = 0.007, *I^2^* = 0.000; *Q* = 3.697, *p* = 0.448) in BP-treated patients compared with the placebo (**Figure 5**; **Table 4**). However, there was no change at the cortical site (SDM = −0.112, 95% CI = −0.593 to 0.368, *p* = 0.646, *I* ^2^ = 0.000; *Q* = 1.284, *p* = 0.526) (**Supplementary Figure 7C**; **Table 4**).

##### True hardness

True hardness (H) represents resistance to purely plastic deformation relative to the EM (35, 36). For the cortical bones, pooled data from two studies (three datasets) for H (SDM = 1.277, 95% CI = 0.040 to 2.514 and *p* = 0.043, *I* ^2^ = 72.684; *Q* = 7.322, *p* = 0.026) (**Figure 5B**) were available. BPs significantly increased H compared with placebo (**Table 4**).

##### Hc and EM

At the cortical site, two studies (three datasets) were analyzed for Hc (SDM = 0.944, 95% CI = −0.399 to 2.286, *p* = 0.168, *I* ^2^ = 77.769; *Q* = 8.997, *p* = 0.011) (**Supplementary Figure 8A**; **Table 4**) and EM data (SDM = −0.043, 95% CI = −0.609 to 0.522, *p* = 0.881, *I^2^* = 14.890; *Q* = 2.350, *p* = 0.309) (**Supplementary Figure 8B**; **Table 4**), which showed no difference between the BP-treated and placebo groups.

#### Effect of SR

Since a sufficient number of studies were available for examining the effect of SR on bone quality and strength, we went ahead with conducting a meta-analysis. SR is used as a treatment for postmenopausal osteoporosis as it has a dual action on bone remodeling by increasing both bone formation and decreasing bone resorption, resulting in the prevention of bone loss and an increase in bone mass and strength (32, 34). Therefore, in this present study, SR could not be included in either the category of anti-resorptive or anabolic.

### DMB and HI

Pooled analysis of two studies (seven datasets) for total bones DMB (SDM = 0.270, 95% CI = −0.077 to 0.618, *p* = 0.127, *I* ^2^ = 0.000; *Q* = 3.128, *p* = 0.793) (**Supplementary Figure 9A**) and HI (SDM = 0.588, 95% CI = −0.014 to 1.190, *p* = 0.055, *I* ^2^ = 60.335; *Q* = 15.127, *p* = 0.019) (**Supplementary Figure 9B**) showed no differences between the SR-treated and placebo groups (**Table 5**).

**Table 5:** Summary of analyzed pooled data from SR of various parameters.

Parameters	Sites	Test of heterogeneity			Test model	Type of association				Level of significance
							95% CI			
		*Q*	*p*-value	*I* ^2^		SDM	Lower limit	Upper limit	*p*-value	
**DMB**	Total bone	3.128	0.793	0.000	Fixed	0.270	−0.077	0.618	0.127	Non-significant
**HI**	Total bone	15.127	0.019	60.335	Random	0.588	−0.014	1.190	0.055	Non-significant

SR, strontium ranelate; DMB, degree of mineralization; HI, heterogeneity index.

### Publication bias

The funnel plot showed an asymmetric distribution in cortical XST (Egger’s regression intercept = 0.51077, *p* = 0.76503) and cancellous MMTR (Egger’s regression intercept = −1.121539, *p* = 0.40103) and MH (Egger’s regression intercept = 6.97054, *p* = 0.00079), suggesting the presence of publication bias in anti-resorptive (BPs and DMab)-treated patients compared to placebo. Thus, we used trim-and-fill analysis to compute unbiased estimates and adjusted the values under the random-effect model. The imputed point estimate for XST was 0.36090 (95% CI = 0.72583 –0.68741), MMTR was 0.54344 (95% CI = 0.18831 – 0.89857), and MH was 0.26402 (95% CI = −0.37207 – 0.90011). Based on Egger’s regression, Duval and Tweedie’s trim and fill, and Begg and Mazumdar’s rank correlation test, we conclude that for the majority of measurements, the computed effects are free of publication bias (**Table 6**).

**Table 6:** Summary of the risk of bias in various parameters included in the study.

Parameters	Sites	Egger’s regression			Test model	Duval and Tweedie’s trim and fill			Begg and Mazumdar rank correlation			Biasness (present/absent)
		Intercept (95% confidence interval)	*p*-value			Studies trimmed	Observed values (95% confidence interval)	Adjusted values (95% confidence interval)	Kendall’s tau with continuity correction			
									Tau	*p*-value		
			1-tailed	2-tailed						1-tailed	2-tailed	
Biasness in pooled data for AFM												
**DMB**	Total	0.91792 (−0.77520, 2.61104)	0.12034	0.24068	**Fixed**	0	0.22957 (−0.03850, 0.49763)	0.22957 (−0.03850, 0.49763)	0.19444	0.23276	0.46551	**Absent**
	Cortical	0.62471 (−0.92136, 2.17078)	0.19226	0.38452	**Fixed**	0	0.20597 (−0.03322, 0.44517)	0.20597 (−0.03322, 0.44517)	0.21818	0.17510	0.35020	**Absent**
	Cancellous	1.76408 (−0.82390, 4.35205)	0.07731	0.15462	**Random**	2	0.19605 (−0.04903, 0.44114)	0.14353 (−0.09251, 0.379581)	0.31111	0.10525	0.21050	**Absent**
**HI**	Total	4.96267 (3.64524, 6.28009)	0.00010	0.00020	**Random**	3	0.58822 (−0.01379, 1.19023)	0.07779 (−0.56738, 0.72296)	0.85714	0.00343	0.00686	**Absent**
	Cortical	−2.31717 (−6.21015, 1.57581)	0.10106	0.20211	**Fixed**	0	0.01955 (−0.27358, 0.31268)	0.01955 (−0.27358, 0.31268)	−0.02778	0.45848	0.91697	**Absent**
	Cancellous	6.16754 (3.01715, 9.31792)	0.00152	0.00303	**Random**	3	0.43374 (−0.16263, 1.03011)	−0.10605 (−0.77982, 0.56772)	0.60714	0.01772	0.03545	**Absent**
**XST**	Cortical	−5.77089 (−10.65839, −0.88339)	0.01307	0.02614	**Random**	0	−1.39372 (−2.5243, −0.26291)	−1.39372 (−2.5243, −0.26291)	−0.62222	0.00613	0.01227	**Absent**
	Cancellous	−3.91548 (−10.00555, 2.17458)	0.07965	0.15930	**Random**	0	−0.90173 (−1.83725, 0.03380)	−0.90173 (−1.83725, 0.03380)	−0.19048	0.27400	0.54801	**Absent**
**MMTR**	Cortical	−8.83086 (−17.31994, −0.34179)	0.02207	0.04413	**Random**	0	−1.30368 (−2.81453, 0.20718)	−1.30368 (−2.81453, 0.20718)	−0.66667	0.01775	0.03550	**Absent**
	Cancellous	−8.26295 (−18.90190, 2.37600)	0.04496	0.08993	**Random**	0	−0.66753 (−1.93077, 0.59572)	−0.66753 (−1.93077, 0.59572)	−0.90000	0.01374	0.02749	**Absent**
**C/P**	Cortical	−2.62097 (−17.16980, 11.92786)	0.13111	0.26221	**Fixed**	0	−0.09296 (−0.44302, 0.25709)	−0.09296 (−0.44302, 0.25709)	−0.66667	0.14813	0.29627	**Absent**
	Cancellous	−1.76704 (−66.47584, 62.94176)	0.39369	0.78738	**Random**	0	−0.25539 (−0.99817, 0.4879)	−0.25539 (−0.99817, 0.4879)	0.00000	0.50000	1.00000	**Absent**
**XLR**	Cortical	−3.24309 (−9.12314, 2.63696)	0.11292	0.22585	**Random**	0	−0.85535 (−1.48136, −0.22934)	−0.85535 (−1.48136, −0.22934)	−0.25000	0.19324	0.38648	**Absent**
	Cancellous	−1.30681 (−3.81482, 1.20119)	0.12884	0.25769	**Fixed**	0	−0.69524 (−0.97075, −0.41973)	−0.69524 (−0.97075, −0.41973)	−0.13889	0.30108	0.60217	**Absent**
Biasness in pooled data for anti-resorptive drugs												
**DMB**	Cortical	−0.23306 (−2.57062, 2.10450)	0.40397	0.80794	**Fixed**	0	0.20834 (−0.06576, 0.48243)	0.20834 (−0.06576, 0.48243)	0.00000	0.50000	1.00000	**Absent**
	Cancellous	3.38901 (−5.5015, 12.32817)	0.17595	0.35191	**Fixed**	1	0.16529 (−0.11743, 0.44802)	0.09040 (−0.17489, 0.35568)	0.40000	0.12983	0.25966	**Absent**
**XST**	Cortical	0.51077 (−5.91761, 6.91761)	0.38251	0.76503	**Fixed**	1	0.38701 (0.04819, 0.72583)	0.36090 (0.72583, 0.68741)	0.00000	0.50000	1.00000	**Absent**
	Cancellous	−3.68972 (−32.48443, 25.10499)	0.17532	0.35064	**Random**	0	−0.14033 (−0.69773, 0.41707)	−0.14033 (−0.69773, 0.41707)	0.00000	0.50000	1.00000	**Absent**
**MMTR**	Cortical	2.48517 (−33.42746, 38.39780)	0.27042	0.54084	**Fixed**	0	0.77076 (0.40710, 1.13442)	0.66149 (0.31552, 1.00745)	0.00000	0.50000	1.00000	**Absent**
	Cancellous	−1.121539 (−12.47343, 10.04266)	0.20051	0.40103	**Fixed**	1	0.54344 (0.18831, 0.89857)	0.54344 (0.18831, 0.89857)	0.00000	0.50000	1.00000	**Absent**
**XLR**	Cortical	1.18053 (−2.38337, 4.74444)	0.14508	0.29015	**Fixed**	2	−0.12188 (−0.45741, 0.21366)	−0.19591 (−0.50238, 0.11057)	0.50000	0.15409	0.30818	**Absent**
	Cancellous	0.01345 (−6.41022, 6.43712)	0.49681	0.99363	**Fixed**	0	−0.53917 (−0.87225, −0.20608)	−0.53917 (−0.87225, −0.20608)	0.00000	0.50000	1.00000	**Absent**
**MH**	Cortical	0.21502 (−5.98981, 6.41985)	0.45957	0.91915	**Fixed**	0	0.85842 (0.55068, 1.16616)	0.85842 (0.55068, 1.16616)	0.30000	0.23122	0.46243	**Absent**
	Cancellous	6.97054 (5.38575, 8.55532)	0.00039	0.00079	**Random**	3	0.86397 (0.22983, 1.49811)	0.26402 (−0.37207, 0.90011)	0.70000	0.04321	0.08641	**Absent**
**Hc**	Cortical	−0.91680 (−24.01329, 22.17970)	0.44005	0.88010	**Random**	0	0.95168 (0.09229, 1.81106)	0.95168 (0.09229, 1.81106)	0.00000	0.50000	1.00000	**Absent**
**H**	Cortical	−4.43024 (−194.22365, 185.36317)	0.40822	0.81644	**Random**	0	1.27688 (0.04008, 2.51368)	1.27688 (0.04008, 2.51368)	0.00000	0.50000	1.00000	**Absent**
**EM**	Cortical	−4.76060 (−12.89968, 3.37848)	0.06411	0.12822	**Fixed**	0	0.18979 (−0.25771, 0.63728)	0.18979 (−0.25771, 0.63728)	−0.50000	0.15409	0.30818	**Absent**
	Cortical	−20.98159 (−26.80824, −15.15494)	0.00696	0.01391	**Random**	0	0.17269 (−0.52698, 0.87236)	0.17269 (−0.52698, 0.87236)	−0.66667	0.14813	0.29627	**Absent**
	Cancellous	−22.13515 (−23.69759, −20.57272)	0.00177	0.00354	**Random**	0	0.13273 (−0.64293, 0.90839	0.13273 (−0.64293, 0.90839)	−0.66667	0.14813	0.29627	**Absent**
Biasness in pooled data for BPs												
**DMB**	Cortical	−0.26666 (−5.01021, 4.47688)	0.43471	0.86941	**Fixed**	0	0.20522 (−0.15550, 0.56594)	0.20522 (−0.15550, 0.56594)	0.100000	0.40325	0.80650	**Absent**
**XST**	Cortical	2.54582 (−4.36510, 9.45673)	0.06700	0.13400	**Fixed**	2	0.25188 (−0.23038, 0.73414)	−0.00000 (−0.40837, 0.40837)	0.00000	0.50000	1.00000	**Absent**
**XLR**	Cortical	2.36093 (−3.68051, 8.40236)	0.06326	0.12652	**Fixed**	2	−0.11243 (−0.59264, 0.36779)	−0.31082 (−0.71568, 0.09404)	0.66667	0.14813	0.29627	**Absent**
	Cancellous	−1.73435 (−4.77010, 1.30140)	0.08331	0.16663	**Fixed**	0	−0.88041 (−1.32072, −0.44009)	−0.88041 (−1.32072, −0.44009)	−0.50000	0.11034	0.22067	**Absent**
**Hc**	Cortical	−3.25898 (−165.01038, 158.49243)	0.42022	0.84045	**Random**	0	0.94360 (−0.39863, 2.28583)	0.94360 (−0.39863, 2.28583)	0.00000	0.50000	1.00000	**Absent**
**EM**	Cortical	−4.39167 (−50.83882, 42.05547)	0.22096	0.44192	**Fixed**	0	−0.04328 (−0.60893, 0.52236)	−0.04328 (−0.60893, 0.52236)	0.00000	0.50000	1.00000	**Absent**
Biasness in pooled data for SR												
**DMB**	Total	1.09661 (−1.62651, 3.81974)	0.17402	0.34803	**Fixed**	0	0.27048 (−0.07719, 0.61816)	0.27048 (−0.07719, 0.61816)	0.28571	0.18376	0.36752	**Absent**
**HI**	Total	4.96259 (3.64483, 6.28035)	0.00010	0.00020	**Random**	3	0.58819 (−0.01382, 1.19020)	0.07775 (−0.56743, 0.72293)	0.85714	0.00343	0.00686	**Absent**

AFM, anti-fracture medications; DMB, degree of mineralization; HI, heterogeneity index; XST, mineral crystallinity; MMTR, mineral–matrix ratio; C/P, carbonate/phosphate ratio; XRL, collagen maturity ratio; MH, microhardness (Kg/mm^2^); Hc, contact hardness (GPa); H, true hardness (GPa); EM, elastic modulus (GPa); BP, bisphosphonates; SR, strontium ranelate.

### Sensitivity analysis

The present study revealed modest changes; within 10% of the pooled estimates and the 95% CIs. No single study was sufficiently sensitive to change the results.

## Discussion

In the pooled analysis, we observed that in comparison to placebo, AFM decreased XST (cortical) and XLR (at both compartments). Subgroup analyses revealed that (a) anti-resorptive drugs (BPs and DMab) increased cortical XST, Hc, MMTR, and MH (at both compartments), and decreased cortical XLR; (b) BPs reduced XLR at the cancellous site and increased H at cortical site compared to placebo; and (c) SR did not affect either the bone material or strength. Assessments of study quality, heterogeneity, publication biases, and sensitivity analysis verified the validity of the findings, suggesting that AFM favorably altered bone material and mechanical behavior.

### The effects on the minerals

Anti-resorptive medication, by increasing bone mass, may have led to an enhanced MMTR by inhibiting osteoclast activity, reducing the bone remodeling rate, and refiling the remodeling area (45, 46). Despite the rise in MMTR, DMB remained unchanged, suggesting that anti-resorptive medications maintain the mineral–organic matrix balance, enhancing stiffness and hardness, and thereby increasing resistance to fracture. Since all studies were from BPs and Ral treatments, the effect of other classes of drugs on DMB needs to be studied.

The C/P ratio is an essential indicator of the chemical composition of the mineral component of bone and an increased C substitution of P in the hydroxyapatite lattice has a negative impact on bone mechanical strength (47, 48). The lack of effect of AFM on the C/P ratio suggests that the bone’s mineralization pattern and maturity level remained relatively stable during the treatment.

AFM in a pooled analysis showed decreased XST, suggesting that hydroxyapatite crystals in bone are less perfect and have smaller crystal sizes that could enhance the energy-absorbing capacity of bone, making it less prone to fractures. Less perfect crystals enhance heterogeneity in bone microarchitecture, composition, and density and are less likely to propagate microfractures through the bone structure. This may contribute to enhanced bone strength and decreased fragility by making it more difficult for microfractures to propagate within the bone. The decrease in XST could be attributable to TPTD, which is known to lower it, resulting in reduced matrix mineralization (29, 49) similar to that seen in young bones. In contrast, a pooled analysis of anti-resorptive medications revealed that DMab appeared to be the main contributor to increased XST through lowering the rate of bone remodeling, which further supports the transition of newly deposited crystals to more mature crystals in size/perfection and accelerates the rate of secondary matrix mineralization (45, 46).

### The effects on collagen

Collagen properties including composition and cross-links (XLR) contribute to bone’s elastic property and only the data on XLR were available in the selected studies. The primary determinant of XLR is the ratio between mature and immature cross-links within the collagen matrix. Higher collagen maturity, which represents mature collagen, typically implies a greater degree of organization and cross-linking of collagen fibers, resulting in a stronger and more stable collagen network within a tissue. Lysyl oxidase (LOX) drives the formation of enzymatic cross-links between and within the collagen fibrils to impart stability to the bone matrix and provide mechanical strength. LOX first catalyzes the formation of divalent reducible collagen cross-links, dehydro-hydoxylysino-norleucine and hydroxylysino-norleucine, which are then matured to become trivalent pyridinium cross-links such as pyrdinoline (PYD), deoxypyridinoline (DPD), and pyrrolic analogs by interacting with aldehyde groups (42, 50). The contribution of enzymatic cross-linking in bone strength has been demonstrated in animal studies, where LOX inhibition by beta aminoproprionitrile resulted in a 50% decrease in pyridinium cross-links leading to reductions in bending strength and modulus of cortical bone, and compressive energy of cancellous bone without affecting stiffness (51, 52). In the vertebrae of human cadaveric specimens, compressive strength correlated with the PYD/DPD ratio but not their concentrations or BMD (53). However, in another study, the degree of anisotropy (orientation and connectivity of trabeculae with the bone matrix) of vertebrae was correlated with vertebral compression strength but not PYD, DPD, and pentosidine (54). Thus, it appears that trabecular microarchitecture contributes to vertebral strength and is notably unaffected by matrix-level composition such as cross-link profile (55). In another study, Patrick Garnero et al. used an *in vitro* model of fetal bovine cortical bone specimens, incubated them at 37°C for 60 days, and showed an increased level of PYD and DPD that were associated with a 30% decrease in bending and compressive yield stress and a 2.5-fold increase in compressive post-yield energy absorption, with no significant change in bone stiffness (56). Thus, an inconclusive relationship was noticed across the studies among cross-linking and strength parameters. Therefore, the extent of enzymatic cross-links that contribute to bone strength at physiological levels remains unclear. AFM and anti-resorptive drugs in pooled analysis lowered XLR, suggesting weaker cross-links. Because the anti-resorptive drugs suppress bone remodeling, the normal turnover of collagen may be disrupted, potentially leading to the accumulation of older collagen and a decrease in collagen maturity. Indeed, subgroup analysis, which was possible only with BPs, showed a significant decrease in XLR compared with placebo. The combined effects of increased BMD and improved mineral properties by the AFM/anti-resorptive drugs including BPs could outweigh the potential negative impact of decreased XLR. Moreover, decreased XLR could reduce the stress concentrations that lead to fractures. However, there are no studies on the types of collagen present, their relative proportions, and their organization within the bone, which could have shed more light on the mechanical behavior of bone.

### The effect on bone mechanics

Nanoindentation measurements provide an understanding of bone mechanics at the microscale, including MH indices, Hc, H, and EM. These measurements specifically determine bone’s resilience to microdamage and its overall material behavior in response to small loads at a very localized level, dependent on hardness and EM (18, 35, 36). While hardness measurements were increased by BPs alone and anti-resorptive drugs (BPs and DMab), EM remained unchanged by these treatments. Furthermore, increased MMTR likely contributed to an increase in the hardness parameters, which could enhance resistance against micro-fractures and their accumulation (57). Thus, it appears that the reduction in fracture risk by AFM goes beyond merely increasing BMD, as it maintains a healthy mineral composition and promotes proper mineralization and deposition within the bone matrix. These effects could contribute to bone’s improved structural integrity and ability to absorb and distribute mechanical forces, reducing the risk of fractures.

### Limitations and future perspectives

The major limitation of this meta-analysis is the inclusion of both randomized control trials (RCTs) and non-RCT studies because the number of RCT-designed studies was insufficient for conducting a meta-analysis. Second, of the various types of osteoporosis, we were able to obtain sufficient data concerning the impacts of AFM on post-menopausal and age-related osteoporosis. The number of studies on the effects of such medications on osteoporosis caused by CKD, GIO, hypertension, IBD, diabetes, and arthritis was insufficient to allow for a meta-analysis. Third, owing to the absence of a placebo group and small sample size, we could not conduct a subgroup analysis to assess the impact of TPTD, DMab, and Ral treatments on bone quality and strength in osteoporosis patients. In addition, in a few studies involving TPTD treatment, patients had previously undergone treatment with BPs (49, 58, 59). Consequently, the specific effects attributed to each drug could not be delineated, leading to the exclusion of these studies from the present meta-analysis. Fourth, High-Resolution Peripheral Quantitative Computed Tomography (HR-pQCT) provides 3D reconstituted data on bone microarchitecture at peripheral sites that go beyond BMD and reveals how bone structures are arranged, interconnected, and oriented, which is crucial for bone strength. There are no studies assessing the effect of anti-osteoporosis drugs on HR-pQCT measures. Fifth, Finite Element Analysis (FEA) is based on simulated bone mechanical testing that is employed to predict how bones respond to forces, stresses, and strains. There were insufficient studies evaluating the response of bones to AFM to different simulated loads and predicting areas of high stress or potential failure by the FEA-based technique (60). Sixth, like BMD, all BMPs are presumed to be negatively influenced by age and estrogen deficiency. However, normative data for BMPs are lacking. The included studies vary in recruitment age and treatment duration, leading to insufficient data for consistent age correction of BMPs. Finally, there are concerns regarding the accumulation of microdamage in response to long-term use of BPs potentially affecting bone strength. There are insufficient studies that assess the extent of microdamage accumulation in the bones of BP-treated patients towards the goal of determining the duration of BP treatment that is safe (61, 62).

## Conclusion

AFM significantly influences bone quality by decreasing mineral crystallinity and XLR. Anti-resorptive (BPs and DMab) drugs promote mineral maturation, improving hardness and deformation resistance. Long-term BP treatment does not compromise bone strength despite reducing collagen maturity. Further research is needed to investigate the effects of BP therapy duration beyond 8 years. Taken together, these findings underscore the multi-scale impact of AFM on bone quality that encompasses changes in both mineralization and matrix properties.

## Data Availability

The original contributions presented in the study are included in the article/**Supplementary Material**. Further inquiries can be directed to the corresponding authors.

## Supplementary material

Supplementary Figure 1 Impact of pooled AFM on **(A, B)** DMB at the indicated- effect sites (a fixed model was used).

Supplementary Figure 2 Impact of pooled AFM on **(A, B)** HI at the indicated sites (a fixed- or random-effect model was used, as designated by a red box).

Supplementary Figure 3 Impact of pooled AFM compared with placebo on **(A)** XST, and **(B, C)** MMTR at the indicated sites (a random-effect model was used).

Supplementary Figure 4 Impact of pooled AFM compared with placebo on **(A, B)** C/P at the indicated sites. A fixed- or random-effect model was used, as designated by a red box.

Supplementary Figure 5 Impact of pooled anti-resorptive treatments compared with placebo on **(A, B)** DMB at the indicated sites. A fixed-effect model was used.

Supplementary Figure 6 Impact of pooled anti-resorptive treatments compared with placebo on **(A)** XST, **(B)** XLR, and **(C)** EM at the indicated sites. A fixed- or random-effect model was used, as designated by a red box.

Supplementary Figure 7 Impact of BPs on **(A)** DMB, **(B)** XST, and **(C)** XLR at the indicated sites. A fixed- effect model was used.

Supplementary Figure 8 Impact of BPs on **(A)** Hc and **(B)** EM compared with placebo at the indicated sites. A fixed- or random-effect model was used, as designated by a red box.

Supplementary Figure 9 Impact of SR on **(A)** DMB and **(B)** HI compared with placebo at the indicated sites. A fixed- or random- effect model was used, as designated by a red box.
