# Supplementary material for: Impact of anti-fracture medications on bone material and strength properties: a systematic review and meta-analysis
Source: Front Endocrinol (Lausanne). 2024 Aug 27;15:1426490. doi: 10.3389/fendo.2024.1426490 (PMC11384599; doi:10.3389/fendo.2024.1426490)
Supplement: Supplementary file 10 [file Table1.doc]

**Impact of anti-fracture medications on bone material and strength properties: a systematic review and meta-analysis**

Shivani Sharma^a,b^, Vijay Shankar^a^, Singh Rajender^a,b^, Ambrish Mithal^c^, Sudhaker D Rao^d,*^, Naibedya Chattopadhyay^a,b^

^a^ Division of Endocrinology and Centre for Research in ASTHI, CSIR-Central Drug Research Institute, Council of Scientific and Industrial Research, Lucknow, 226031, India.

^b^ Academy of Scientific and Innovative Research (AcSIR), Ghaziabad- 201002, India.

^c^ Institute of Endocrinology and Diabetes, Max Healthcare, Institutional Area, Press Enclave Road, Saket, New Delhi, India.

^d^ Division of Endocrinology Diabetes and Bone & Mineral Disorders, and Bone and Mineral Research Laboratory, Henry Ford Health/Michigan State University College of Human Medicine, Detroit, [Michigan](https://en.wikipedia.org/wiki/Michigan), USA.

* Corresponding author: Sudhaker D. Rao

Henry Ford Medical Center, New Center One

3031 W. Grand Blvd; Suite # 800

Detroit, MI, 48202

**Keywords:** Bone-quality, Bone-strength, Osteoporosis, Bisphosphonate, Denosumab, Raloxifene, Teriparatide, Strontium-ranelate.

**Suppl. Table 1:** Search strategy

**Keywords in Google S**cholar

| "Bisphosphonate" AND "Osteoporosis" AND "carbonate to phosphate ratio" AND "Human" |
| --- |
| "Bisphosphonate" AND "Osteoporosis" AND "carbonate to amide ratio" AND "Human" |
| "Bisphosphonate" AND "Osteoporosis" AND "Mineral matrix ratio" AND "Human" |
| "Bisphosphonate" AND "Osteoporosis" AND "Collagen maturity" AND "Human" |
| "Bisphosphonate" AND "Osteoporosis" AND "Heterogeneity index" AND "Human" |
| "Bisphosphonate" AND "Osteoporosis" AND "Pentosidine" AND "Human" |
| "Bisphosphonate" AND "Osteoporosis" AND "Degree of mineralization" AND "Human" |
| "Bisphosphonate" AND "Osteoporosis" AND "Homocysteine" AND "Human" AND "Clinical trial" |
| "Bisphosphonate" AND "Osteoporosis" AND "FEA" AND "Human" AND "Clinical trial" |
| "Bisphosphonate" AND "Osteoporosis" AND "3 point bending" AND "Human" AND "Clinical trial" |
| "Bisphosphonate" AND "Osteoporosis" AND "Nanoindentation" AND "Human" AND “Clinical trial” |
| "Bisphosphonate" AND "Osteoporosis" AND "Crystallinity" AND "Human" AND “Clinical trial” |
| "Bisphosphonate"AND "Osteoporosis" AND "Advanced glycation end products (AGEs)" AND "Human" |
| "Bisphosphonate" AND "Osteoporosis" AND "Bone loss" AND "Human" AND “Clinical trial” |
| "Bisphosphonate" AND "osteoporosis" AND "Enzymatic to non-enzymatic cross-linking ratio" AND "Human" |
| "Denosumab" AND "Osteoporosis" AND "carbonate to phosphate ratio" AND "Human" |
| "Denosumab" AND "Osteoporosis" AND "carbonate to amide ratio" AND "Human" |
| "Denosumab" AND "Osteoporosis" AND "Mineral matrix ratio" AND "Human" |
| "Denosumab" AND "Osteoporosis" AND "Collagen maturity" AND "Human" |
| "Denosumab" AND "Osteoporosis" AND "Microdamage accumulation" AND "Human" |
| AND "Osteoporosis" AND "Heterogeneity index" AND "Human" |
| "Denosumab" AND "Osteoporosis" AND "Pentosidine" AND "Human" |
| "Denosumab" AND "Osteoporosis" AND "Degree of mineralization" AND "Human" |
| "Denosumab" AND "Osteoporosis" AND "Homocysteine" AND "Human" AND "Clinical trial" |
| "Denosumab" AND "Osteoporosis" AND "FEA" AND "Human" AND "Clinical trial" |
| "Denosumab'' AND "Osteoporosis" AND "3 point bending" AND "Human" AND "Clinical trial" |
| "Denosumab'' AND "Osteoporosis" AND "Nanoindentation" AND "Human" AND “Clinical trial” |
| "Denosumab'' AND "Osteoporosis" AND "Crystallinity" AND "Human" AND “Clinical trial” |
| "Denosumab'' AND "Osteoporosis" AND "Advanced glycation end products (AGEs)" AND "Human" |
| "Denosumab'' AND "Osteoporosis" AND "Bone loss" |
| "Denosumab" AND "osteoporosis" AND "Enzymatic to non-enzymatic cross-linking ratio" AND "Human" |
| "Teriparatide" AND "Osteoporosis" AND "carbonate to phosphate ratio" AND "Human" |
| "Teriparatide" AND "Osteoporosis" AND "carbonate to amide ratio" |
| "Teriparatide" AND "Osteoporosis" AND "Mineral matrix ratio" AND "Human" |
| "Teriparatide" AND "Osteoporosis" AND "Collagen maturity" AND "Human" |
| "Teriparatide" AND "Osteoporosis" AND "Microdamage accumulation" AND "Human" |
| "Teriparatide" AND "Osteoporosis" AND "Heterogeneity index" AND "Human" |
| "Teriparatide" AND "Osteoporosis" AND "Pentosidine" AND "Human" |
| "Teriparatide" AND "Osteoporosis" AND "Degree of mineralization" AND "Human" |
| "Teriparatide" AND "Osteoporosis" AND "Homocysteine" AND "Human" |
| "Teriparatide" AND "Osteoporosis" AND "FEA" AND "Human" |
| "Teriparatide" AND "Osteoporosis" AND "3 point bending" AND "Human" |
| "Teriparatide" AND "Osteoporosis" AND "Nanoindentation" AND "Human" |
| "Teriparatide" AND "Osteoporosis" AND "Crystallinity" AND "Human" |
| "Teriparatide" AND "Osteoporosis" AND "Advanced glycation end products (AGEs)" AND "Human" |
| "Teriparatide" AND "Osteoporosis" AND "Bone loss" AND "Human" |
| "Teriparatide" AND "osteoporosis" AND "Enzymatic to non-enzymatic cross-linking ratio" AND "Human" |
| "Abaloparatide" AND "Osteoporosis" AND "carbonate to phosphate ratio" AND "Human" |
| "Abaloparatide" AND "Osteoporosis" AND "carbonate to amide ratio" |
| "Abaloparatide" AND "Osteoporosis" AND "Mineral matrix ratio" AND "Human" |
| "Abaloparatide" AND "Osteoporosis" AND "Collagen maturity" AND "Human" |
| "Abaloparatide" AND "Osteoporosis" AND "Microdamage accumulation" AND "Human" |
| "Abaloparatide" AND "Osteoporosis" AND "Heterogeneity index" AND "Human" |
| "Abaloparatide" AND "Osteoporosis" AND "Pentosidine" AND "Human" |
| "Abaloparatide" AND "Osteoporosis" AND "Degree of mineralization" AND "Human" |
| "Abaloparatide" AND "Osteoporosis" AND "Homocysteine" AND "Human" |
| "Abaloparatide" AND "Osteoporosis" AND "FEA" AND "Human" |
| "Abaloparatide" AND "Osteoporosis" AND "3 point bending" AND "Human" |
| "Abaloparatide" AND "Osteoporosis" AND "Nanoindentation" AND "Human" |
| "Abaloparatide" AND "Osteoporosis" AND "Crystallinity" AND "Human" |
| "Abaloparatide" AND "Osteoporosis" AND "Advanced glycation end products (AGEs)" AND "Human" |
| "Abaloparatide" AND "Osteoporosis" AND "Bone loss" AND "Human" |
| "Abaloparatide" AND "osteoporosis" AND "Enzymatic to non-enzymatic cross-linking ratio" AND "Human" |
| "Raloxifene" AND "Osteoporosis" AND "carbonate to phosphate ratio" AND "Human" |
| "Raloxifene" AND "Osteoporosis" AND "carbonate to amide ratio" |
| "Raloxifene" AND "Osteoporosis" AND "Mineral matrix ratio" AND "Human" |
| "Raloxifene" AND "Osteoporosis" AND "Collagen maturity" AND "Human" |
| "Raloxifene" AND "Osteoporosis" AND "Microdamage accumulation" AND "Human" |
| "Raloxifene" AND "Osteoporosis" AND "Heterogeneity index" AND "Human" |
| "Raloxifene" AND "Osteoporosis" AND "Pentosidine" AND "Human" |
| "Raloxifene" AND "Osteoporosis" AND "Degree of mineralization" AND "Human" |
| "Raloxifene" AND "Osteoporosis" AND "Homocysteine" AND "Human" |
| "Raloxifene" AND "Osteoporosis" AND "FEA" AND "Human" |
| "Raloxifene" AND "Osteoporosis" AND "3 point bending" AND "Human" |
| "Raloxifene" AND "Osteoporosis" AND "Nanoindentation" AND "Human" |
| "Raloxifene" AND "Osteoporosis" AND "Crystallinity" AND "Human" |
| "Raloxifene" AND "Osteoporosis" AND "Advanced glycation end products (AGEs)" AND "Human" |
| "Raloxifene" AND "Osteoporosis" AND "Bone loss" AND "Human" |
| "Raloxifene" AND "osteoporosis" AND "Enzymatic to non-enzymatic cross-linking ratio" AND "Human" |
| "Romosozumab" AND "Osteoporosis" AND "carbonate to phosphate ratio" AND "Human" |
| "Romosozumab" AND "Osteoporosis" AND "carbonate to amide ratio" |
| "Romosozumab" AND "Osteoporosis" AND "Mineral matrix ratio" AND "Human" |
| "Romosozumab" AND "Osteoporosis" AND "Collagen maturity" AND "Human" |
| "Romosozumab" AND "Osteoporosis" AND "Microdamage accumulation" AND "Human" |
| "Romosozumab" AND "Osteoporosis" AND "Heterogeneity index" AND "Human" |
| "Romosozumab" AND "Osteoporosis" AND "Pentosidine" AND "Human" |
| "Romosozumab" AND "Osteoporosis" AND "Degree of mineralization" AND "Human" |
| "Romosozumab" AND "Osteoporosis" AND "Homocysteine" AND "Human" |
| "Romosozumab” AND "Osteoporosis" AND “FEA" AND "Human" |
| "Romosozumab" AND "Osteoporosis" AND “3 point bending" AND "Human" |
| "Romosozumab" AND "Osteoporosis" AND “Nanoindentation" AND "Human" |
| "Romosozumab" AND "Osteoporosis" AND "Crystallinity" AND "Human" |
| "Romosozumab" AND "Osteoporosis" AND "Advanced glycation end products (AGEs)" AND "Human" |
| "Romosozumab” AND "Osteoporosis" AND "Bone loss" AND "Human" |
| "Romosozumab" AND "osteoporosis" AND "Enzymatic to non-enzymatic cross-linking ratio" AND "Human" |
| "Strontium ranelate" AND "Osteoporosis" AND "carbonate to phosphate ratio" AND "Human" |
| "Strontium ranelate" AND "Osteoporosis" AND "carbonate to amide ratio" |
| "Strontium ranelate" AND "Osteoporosis" AND “Mineral matrix ratio" AND "Human" |
| "Strontium ranelate" AND "Osteoporosis" AND “Collagen maturity" AND "Human" |
| "Strontium ranelate" AND "Osteoporosis" AND “Microdamage accumulation" AND "Human" |
| "Strontium ranelate" AND "Osteoporosis" AND “Heterogeneity index" AND "Human" |
| "Strontium ranelate" AND "Osteoporosis" AND “Pentosidine" AND "Human" |
| "Strontium ranelate” AND "Osteoporosis" AND “Degree of mineralization" AND "Human" |
| "Strontium ranelate” AND "Osteoporosis" AND “Homocysteine" AND "Human" |
| "Strontium ranelate” AND "Osteoporosis" AND “FEA" AND "Human" |
| "Strontium ranelate" AND "Osteoporosis" AND “3 point bending" AND "Human" |
| "Strontium ranelate" AND "Osteoporosis" AND “Nanoindentation" AND "Human" |
| "Strontium ranelate" AND "Osteoporosis" AND "Crystallinity" AND "Human" |
| "Strontium ranelate" AND "Osteoporosis" AND "Advanced glycation end products (AGEs)" AND "Human" |
| "Strontium ranelate” AND "Osteoporosis" AND "Bone loss" AND "Human" |
| "Strotium ranelate" AND "osteoporosis" AND "Enzymatic to non-enzymatic cross-linking ratio" AND "Human" |

| " Strontium ranelate " AND "Osteoporosis" AND “IBD” AND "Crystallinity" AND "Human" AND “Clinical trial” |
| --- |
| "Abaloparatide” AND “Arthritis”AND "Osteoporosis" AND “Collagen maturity" AND "Human" |
| "Abaloparatide” AND “CKD”AND "Osteoporosis" AND “Collagen maturity" AND "Human" |
| "Abaloparatide” AND “DIABETES”AND "Osteoporosis" AND "Collagen maturity" AND "Human" |
| "Abaloparatide" AND “GIO”AND "Osteoporosis" AND "Collagen maturity" AND "Human" |
| "Abaloparatide" AND “Hypertension”AND "Osteoporosis" AND "Collagen maturity" AND "Human" |
| "Abaloparatide" AND “IBD”AND "Osteoporosis" AND "Collagen maturity" AND "Human" |
| "Abaloparatide" AND "Osteoporosis" AND “Arthritis”AND "FEA" AND "Human" AND "Clinical trial" |
| "Abaloparatide" AND "Osteoporosis" AND “Arthritis”AND "Homocysteine" AND "Human" AND "Clinical trial" |
| "Abaloparatide" AND "Osteoporosis" AND “CKD”AND "FEA" AND "Human" AND "Clinical trial" |
| "Abaloparatide" AND "Osteoporosis" AND “CKD”AND "Homocysteine" AND "Human" AND "Clinical trial" |
| "Abaloparatide" AND "Osteoporosis" AND “DIABETES”AND "FEA" AND "Human" AND "Clinical trial" |
| "Abaloparatide" AND "Osteoporosis" AND “DIABETES”AND "Homocysteine" AND "Human" AND "Clinical trial" |
| "Abaloparatide" AND "Osteoporosis" AND “GIO”AND "FEA" AND "Human" AND "Clinical trial" |
| "Abaloparatide" AND "Osteoporosis" AND “GIO”AND "Homocysteine" AND "Human" AND "Clinical trial" |
| "Abaloparatide" AND "Osteoporosis" AND “Hypertension”AND "FEA" AND "Human" AND "Clinical trial" |
| "Abaloparatide" AND "Osteoporosis" AND “Hypertension”AND "Homocysteine" AND "Human" AND "Clinical trial" |
| "Abaloparatide" AND "Osteoporosis" AND “IBD”AND "FEA" AND "Human" AND "Clinical trial" |
| "Abaloparatide" AND "Osteoporosis" AND “IBD”AND "Homocysteine" AND "Human" AND "Clinical trial" |
| "Abaloparatide" AND "Osteoporosis" AND “Arthritis” AND "3 point bending" AND "Human" AND "Clinical trial" |
| "Abaloparatide" AND "Osteoporosis" AND “Arthritis” AND "Degree of mineralization" AND "Human" |
| "Abaloparatide" AND "Osteoporosis" AND “Arthritis” AND "Heterogeneity index" AND "Human" |
| "Abaloparatide" AND "Osteoporosis" AND “Arthritis” AND "Microdamage accumulation" AND "Human" |
| "Abaloparatide" AND "Osteoporosis" AND “Arthritis” AND "Mineral matrix ratio" AND "Human" |
| "Abaloparatide" AND "Osteoporosis" AND “Arthritis” AND "Nanoindentation" AND "Human" AND “Clinical trial” |
| "Abaloparatide" AND "Osteoporosis" AND “Arthritis” AND “Pentosidine" AND "Human" |
| "Abaloparatide" AND "Osteoporosis" AND “Arthritis” AND "Bone loss" AND "Human" AND “Clinical trial” |
| "Abaloparatide" AND "Osteoporosis" AND “Arthritis” AND "carbonate to amide ratio" AND "Human" |
| "Abaloparatide" AND "Osteoporosis" AND “Arthritis” AND "Crystallinity" AND "Human" AND “Clinical trial” |
| "Abaloparatide" AND "osteoporosis" AND “Arthritis” AND "Enzymatic to non-enzymatic cross-linking ratio" AND "Human" |
| "Abaloparatide" AND "Osteoporosis" AND “Arthritis “AND "carbonate to phosphate ratio" AND "Human" |
| "Abaloparatide" AND "Osteoporosis" AND “CKD” AND "3 point bending" AND "Human" AND "Clinical trial" |
| "Abaloparatide" AND "Osteoporosis" AND “CKD” AND "Degree of mineralization" AND "Human" |
| "Abaloparatide" AND "Osteoporosis" AND “CKD” AND “Heterogeneity index" AND "Human" |
| "Abaloparatide" AND "Osteoporosis" AND “CKD” AND “Microdamage accumulation" AND "Human" |
| "Abaloparatide" AND "Osteoporosis" AND “CKD” AND “Mineral matrix ratio" AND "Human" |
| "Abaloparatide" AND "Osteoporosis" AND “CKD” AND “Nanoindentation" AND "Human" AND “Clinical trial” |
| "Abaloparatide" AND "Osteoporosis" AND “CKD” AND “Pentosidine" AND "Human" |
| "Abaloparatide" AND "Osteoporosis" AND “CKD” AND "Bone loss" AND "Human" AND “Clinical trial” |
| "Abaloparatide" AND "Osteoporosis" AND “CKD” AND "carbonate to amide ratio" AND "Human" |
| "Abaloparatide" AND "Osteoporosis" AND “CKD” AND "Crystallinity" AND "Human" AND “Clinical trial” |
| "Abaloparatide" AND "osteoporosis" AND “CKD” AND "Enzymatic to non-enzymatic cross-linking ratio" AND "Human" |
| "Abaloparatide" AND "Osteoporosis" AND “CKD”AND "carbonate to phosphate ratio" AND "Human" |
| "Abaloparatide" AND "Osteoporosis" AND “DIABETES” AND “3 point bending" AND "Human" AND "Clinical trial" |
| "Abaloparatide" AND "Osteoporosis" AND “DIABETES” AND “Degree of mineralization" AND "Human" |
| "Abaloparatide" AND "Osteoporosis" AND “DIABETES” AND “Heterogeneity index" AND "Human" |
| "Abaloparatide" AND "Osteoporosis" AND “DIABETES” AND “Microdamage accumulation" AND "Human" |
| "Abaloparatide" AND "Osteoporosis" AND “DIABETES” AND “Mineral matrix ratio" AND "Human" |
| "Abaloparatide" AND "Osteoporosis" AND “DIABETES” AND “Nanoindentation" AND "Human" AND “Clinical trial” |
| "Abaloparatide" AND "Osteoporosis" AND “DIABETES” AND “Pentosidine" AND "Human" |
| "Abaloparatide" AND "Osteoporosis" AND “DIABETES” AND "Bone loss" AND "Human" AND “Clinical trial” |
| "Abaloparatide" AND "Osteoporosis" AND “DIABETES” AND "carbonate to amide ratio" AND "Human" |
| "Abaloparatide" AND "Osteoporosis" AND “DIABETES” AND "Crystallinity" AND "Human" AND “Clinical trial” |
| "Abaloparatide" AND "osteoporosis" AND “DIABETES” AND "Enzymatic to non-enzymatic cross-linking ratio" AND "Human" |
| "Abaloparatide" AND "Osteoporosis" AND “DIABETES”AND "carbonate to phosphate ratio" AND "Human" |
| "Abaloparatide" AND "Osteoporosis" AND “GIO” AND “3 point bending" AND "Human" AND "Clinical trial" |
| "Abaloparatide" AND "Osteoporosis" AND “GIO” AND “Degree of mineralization" AND "Human" |
| "Abaloparatide" AND "Osteoporosis" AND “GIO” AND “Heterogeneity index" AND "Human" |
| "Abaloparatide" AND "Osteoporosis" AND “GIO” AND “Microdamage accumulation" AND "Human" |
| "Abaloparatide" AND "Osteoporosis" AND “GIO” AND “Mineral matrix ratio" AND "Human" |
| "Abaloparatide" AND "Osteoporosis" AND “GIO” AND “Nanoindentation" AND "Human" AND “Clinical trial” |
| "Abaloparatide" AND "Osteoporosis" AND “GIO” AND “Pentosidine" AND "Human" |
| "Abaloparatide" AND "Osteoporosis" AND “GIO” AND "Bone loss" AND "Human" AND “Clinical trial” |
| "Abaloparatide" AND "Osteoporosis" AND “GIO” AND "carbonate to amide ratio" AND "Human" |
| "Abaloparatide" AND "Osteoporosis" AND “GIO” AND "Crystallinity" AND "Human" AND “Clinical trial” |
| "Abaloparatide" AND "osteoporosis" AND “GIO” AND "Enzymatic to non-enzymatic cross-linking ratio" AND "Human" |
| "Abaloparatide" AND "Osteoporosis" AND “GIO”AND "carbonate to phosphate ratio" AND "Human" |
| "Abaloparatide" AND "Osteoporosis" AND “Hypertension” AND “3 point bending" AND "Human" AND "Clinical trial" |
| "Abaloparatide" AND "Osteoporosis" AND “Hypertension” AND “Degree of mineralization" AND "Human" |
| "Abaloparatide" AND "Osteoporosis" AND “Hypertension” AND “Heterogeneity index" AND "Human" |
| "Abaloparatide" AND "Osteoporosis" AND “Hypertension” AND “Microdamage accumulation" AND "Human" |
| "Abaloparatide" AND "Osteoporosis" AND “Hypertension” AND “Mineral matrix ratio" AND "Human" |
| "Abaloparatide" AND "Osteoporosis" AND “Hypertension” AND “Nanoindentation" AND "Human" AND “Clinical trial” |
| "Abaloparatide" AND "Osteoporosis" AND “Hypertension” AND “Pentosidine" AND "Human" |
| "Abaloparatide" AND "Osteoporosis" AND “Hypertension” AND "Bone loss" AND "Human" AND “Clinical trial” |
| "Abaloparatide" AND "Osteoporosis" AND “Hypertension” AND "carbonate to amide ratio" AND "Human" |
| "Abaloparatide" AND "Osteoporosis" AND “Hypertension” AND "Crystallinity" AND "Human" AND “Clinical trial” |
| "Abaloparatide" AND "osteoporosis" AND “Hypertension” AND "Enzymatic to non-enzymatic cross-linking ratio" AND "Human" |
| "Abaloparatide" AND "Osteoporosis" AND “Hypertension”AND "carbonate to phosphate ratio" AND "Human" |
| "Abaloparatide" AND "Osteoporosis" AND “IBD” AND “3 point bending" AND "Human" AND " Clinical trial " |
| "Abaloparatide" AND "Osteoporosis" AND “IBD” AND “Degree of mineralization" AND "Human" |
| "Abaloparatide" AND "Osteoporosis" AND “IBD” AND “Heterogeneity index" AND "Human" |
| "Abaloparatide" AND "Osteoporosis" AND “IBD” AND “Microdamage accumulation" AND "Human" |
| "Abaloparatide" AND "Osteoporosis" AND “IBD” AND “Mineral matrix ratio" AND "Human" |
| "Abaloparatide" AND "Osteoporosis" AND “IBD” AND “Nanoindentation" AND "Human" AND “Clinical trial” |
| "Abaloparatide" AND "Osteoporosis" AND “IBD” AND “Pentosidine" AND "Human" |
| "Abaloparatide" AND "Osteoporosis" AND “IBD” AND "Bone loss" AND "Human" AND “Clinical trial” |
| "Abaloparatide" AND "Osteoporosis" AND “IBD” AND "carbonate to amide ratio" AND "Human" |
| "Abaloparatide" AND "Osteoporosis" AND “IBD” AND "Crystallinity" AND "Human" AND “Clinical trial” |
| "Abaloparatide" AND "osteoporosis" AND “IBD” AND "Enzymatic to non-enzymatic cross-linking ratio" AND "Human" |
| "Abaloparatide" AND "Osteoporosis" AND “IBD”AND "carbonate to phosphate ratio" AND "Human" |
| "Abaloparatide" AND "Osteoporosis" AND “Arthritis” AND "Advanced glycation end products (AGEs)" AND "Human" |
| "Abaloparatide" AND "Osteoporosis" AND “CKD” AND "Advanced glycation end products (AGEs)" AND "Human" |
| "Abaloparatide" AND "Osteoporosis" AND “DIABETES” AND "Advanced glycation end products (AGEs)" AND "Human" |
| "Abaloparatide" AND "Osteoporosis" AND “GIO” AND "Advanced glycation end products (AGEs)" AND "Human" |
| "Abaloparatide" AND "Osteoporosis" AND “Hypertension” AND "Advanced glycation end products (AGEs)" AND "Human" |
| "Abaloparatide" AND "Osteoporosis" AND “IBD” AND "Advanced glycation end products (AGEs)" AND "Human" |
| "Bisphosphonate" AND “Arthritis “AND "Osteoporosis" AND "Collagen maturity" AND "Human" |
| "Bisphosphonate" AND “CKD”AND "Osteoporosis" AND "Collagen maturity" AND "Human" |
| "Bisphosphonate" AND “DIABETES”AND "Osteoporosis" AND "Collagen maturity" AND "Human" |
| "Bisphosphonate" AND “GIO”AND "Osteoporosis" AND "Collagen maturity" AND "Human" |
| "Bisphosphonate" AND “Hypertension” AND "Osteoporosis" AND "Collagen maturity" AND "Human" |
| "Bisphosphonate" AND “IBD”AND "Osteoporosis" AND "Collagen maturity" AND "Human" |
| "Bisphosphonate" AND "Osteoporosis" AND “Arthritis” AND "FEA" AND "Human" AND “Clinical trial” |
| "Bisphosphonate" AND "Osteoporosis" AND “Arthritis” AND "Homocysteine" AND "Human" AND “Clinical trial” |
| "Bisphosphonate" AND "Osteoporosis" AND “CKD”AND "FEA" AND "Human" AND “Clinical trial" |
| "Bisphosphonate" AND "Osteoporosis" AND “CKD”AND "Homocysteine" AND "Human" AND “Clinical trial” |
| "Bisphosphonate" AND "Osteoporosis" AND “DIABETES”AND "FEA" AND "Human" AND "Clinical trial" |
| "Bisphosphonate" AND "Osteoporosis" AND “DIABETES”AND "Homocysteine" AND "Human" AND “Clinical trial” |
| "Bisphosphonate" AND "Osteoporosis" AND “GIO”AND "FEA" AND "Human" AND "Clinical trial" |
| "Bisphosphonate" AND "Osteoporosis" AND “GIO”AND "Homocysteine" AND "Human" AND "Clinical trial" |
| "Bisphosphonate" AND "Osteoporosis" AND “Hypertension”AND "FEA" AND "Human" AND "Clinical trial" |
| "Bisphosphonate" AND "Osteoporosis" AND “Hypertension”AND "Homocysteine" AND "Human" AND "Clinical trial" |
| "Bisphosphonate" AND "Osteoporosis" AND “IBD”AND "FEA" AND "Human" AND "Clinical trial" |
| "Bisphosphonate" AND "Osteoporosis" AND “IBD”AND "Homocysteine" AND "Human" AND "Clinical trial" |
| "Bisphosphonate" AND "Osteoporosis" AND “Arthritis” AND "3 point bending" AND "Human" AND "Clinical trial" |
| "Bisphosphonate" AND "Osteoporosis" AND “Arthritis” AND "Degree of mineralization" AND "Human" |
| "Bisphosphonate" AND "Osteoporosis" AND “Arthritis” AND "Heterogeneity index" AND "Human" |
| "Bisphosphonate" AND "Osteoporosis" AND “Arthritis” AND "Microdamage accumulation" AND "Human" |
| "Bisphosphonate" AND "Osteoporosis" AND “Arthritis” AND "Mineral matrix ratio" AND "Human" |
| "Bisphosphonate" AND "Osteoporosis" AND “Arthritis” AND "Nanoindentation" AND "Human" AND “Clinical trial” |
| "Bisphosphonate" AND "Osteoporosis" AND “Arthritis” AND "Pentosidine" AND "Human" |
| "Bisphosphonate" AND "Osteoporosis" AND “Arthritis” AND "Bone loss" AND "Human" AND “Clinical trial” |
| "Bisphosphonate" AND "Osteoporosis" AND “Arthritis” AND "carbonate to amide ratio" AND "Human" |
| "Bisphosphonate" AND "Osteoporosis" AND “Arthritis” AND "Crystallinity" AND "Human" AND “Clinical trial” |
| "Bisphosphonate" AND "osteoporosis" AND “Arthritis” AND "Enzymatic to non-enzymatic cross-linking ratio" AND "Human" |
| "Bisphosphonate" AND "Osteoporosis" AND “Arthritis” AND "carbonate to phosphate ratio" AND "Human" |
| "Bisphosphonate" AND "Osteoporosis" AND “CKD” AND "3 point bending" AND "Human" AND "Clinical trial" |
| "Bisphosphonate" AND "Osteoporosis" AND “CKD” AND "Degree of mineralization" AND "Human" |
| "Bisphosphonate" AND "Osteoporosis" AND “CKD” AND "Heterogeneity index" AND "Human" |
| "Bisphosphonate" AND "Osteoporosis" AND “CKD” AND "Microdamage accumulation" AND "Human" |
| "Bisphosphonate" AND "Osteoporosis" AND “CKD” AND "Mineral matrix ratio" AND "Human" |
| "Bisphosphonate" AND "Osteoporosis" AND “CKD” AND "Nanoindentation" AND "Human" AND “Clinical trial” |
| "Bisphosphonate" AND "Osteoporosis" AND “CKD” AND "Pentosidine" AND "Human" |
| "Bisphosphonate" AND "Osteoporosis" AND “CKD” AND "Bone loss" AND "Human" AND “Clinical trial” |
| "Bisphosphonate" AND "Osteoporosis" AND “CKD” AND "carbonate to amide ratio" AND "Human" |
| "Bisphosphonate" AND "Osteoporosis" AND “CKD” AND "Crystallinity" AND "Human" AND “Clinical trial” |
| "Bisphosphonate" AND "osteoporosis" AND “CKD” AND "Enzymatic to non-enzymatic cross-linking ratio" AND "Human" |
| "Bisphosphonate" AND "Osteoporosis" AND “CKD”AND "carbonate to phosphate ratio" AND "Human" |
| "Bisphosphonate" AND "Osteoporosis" AND “DIABETES” AND "3 point bending" AND "Human" AND "Clinical trial" |
| "Bisphosphonate" AND "Osteoporosis" AND “DIABETES” AND "Degree of mineralization" AND "Human" |
| "Bisphosphonate" AND "Osteoporosis" AND “DIABETES” AND "Heterogeneity index" AND "Human" |
| "Bisphosphonate" AND "Osteoporosis" AND “DIABETES” AND "Microdamage accumulation" AND "Human" |
| "Bisphosphonate" AND "Osteoporosis" AND “DIABETES” AND "Mineral matrix ratio" AND "Human" |
| "Bisphosphonate" AND "Osteoporosis" AND “DIABETES” AND "Nanoindentation" AND "Human" AND “Clinical trial” |
| "Bisphosphonate" AND "Osteoporosis" AND “DIABETES” AND "Pentosidine" AND "Human" |
| "Bisphosphonate" AND "Osteoporosis" AND “DIABETES” AND "Bone loss" AND "Human" AND “Clinical trial” |
| "Bisphosphonate" AND "Osteoporosis" AND “DIABETES” AND "carbonate to amide ratio" AND "Human" |
| "Bisphosphonate" AND "Osteoporosis" AND “DIABETES” AND "Crystallinity" AND "Human" AND “Clinical trial” |
| "Bisphosphonate" AND "osteoporosis" AND “DIABETES” AND "Enzymatic to non-enzymatic cross-linking ratio" AND "Human" |
| "Bisphosphonate" AND "Osteoporosis" AND “DIABETES”AND "carbonate to phosphate ratio" AND "Human" |
| "Bisphosphonate" AND "Osteoporosis" AND “GIO” AND "3 point bending" AND "Human" AND "Clinical trial" |
| "Bisphosphonate" AND "Osteoporosis" AND “GIO” AND "Degree of mineralization" AND "Human" |
| "Bisphosphonate" AND "Osteoporosis" AND “GIO” AND "Heterogeneity index" AND "Human" |
| "Bisphosphonate" AND "Osteoporosis" AND “GIO” AND "Microdamage accumulation" AND "Human" |
| "Bisphosphonate" AND "Osteoporosis" AND “GIO” AND "Mineral matrix ratio" AND "Human" |
| "Bisphosphonate" AND "Osteoporosis" AND “GIO” AND "Nanoindentation" AND "Human" AND “Clinical trial” |
| "Bisphosphonate" AND "Osteoporosis" AND “GIO” AND "Pentosidine" AND "Human" |
| "Bisphosphonate" AND "Osteoporosis" AND “GIO” AND "Bone loss" AND "Human" AND “Clinical trial” |
| "Bisphosphonate" AND "Osteoporosis" AND “GIO” AND "carbonate to amide ratio" AND "Human" |
| "Bisphosphonate" AND "Osteoporosis" AND “GIO” AND "Crystallinity" AND "Human" AND “Clinical trial” |
| "Bisphosphonate" AND "osteoporosis" AND “GIO” AND "Enzymatic to non-enzymatic cross-linking ratio" AND "Human" |
| "Bisphosphonate" AND "Osteoporosis" AND “GIO”AND "carbonate to phosphate ratio" AND "Human" |
| "Bisphosphonate" AND "Osteoporosis" AND “Hypertension” AND "3 point bending" AND "Human" AND "Clinical trial" |
| "Bisphosphonate" AND "Osteoporosis" AND “Hypertension” AND "Degree of mineralization" AND "Human" |
| "Bisphosphonate" AND "Osteoporosis" AND “Hypertension” AND "Heterogeneity index" AND "Human" |
| "Bisphosphonate" AND "Osteoporosis" AND “Hypertension” AND "Microdamage accumulation" AND "Human" |
| "Bisphosphonate" AND "Osteoporosis" AND “Hypertension” AND "Mineral matrix ratio" AND "Human" |
| "Bisphosphonate" AND "Osteoporosis" AND “Hypertension” AND "Nanoindentation" AND "Human" AND “Clinical trial” |
| "Bisphosphonate" AND "Osteoporosis" AND “Hypertension” AND "Pentosidine" AND "Human" |
| "Bisphosphonate" AND "Osteoporosis" AND “Hypertension” AND "Bone loss" AND "Human" AND “Clinical trial” |
| "Bisphosphonate" AND "Osteoporosis" AND “Hypertension” AND "carbonate to amide ratio" AND "Human" |
| "Bisphosphonate" AND "Osteoporosis" AND “Hypertension” AND "Crystallinity" AND "Human" AND “Clinical trial” |
| "Bisphosphonate" AND "osteoporosis" AND “Hypertension” AND "Enzymatic to non-enzymatic cross-linking ratio" AND "Human" |
| "Bisphosphonate" AND "Osteoporosis" AND “Hypertension”AND "carbonate to phosphate ratio" AND "Human" |
| "Bisphosphonate" AND "Osteoporosis" AND “IBD” AND "3 point bending" AND "Human" AND "Clinical trial" |
| "Bisphosphonate" AND "Osteoporosis" AND “IBD” AND "Degree of mineralization" AND "Human" |
| "Bisphosphonate" AND "Osteoporosis" AND “IBD” AND "Heterogeneity index" AND "Human" |
| "Bisphosphonate" AND "Osteoporosis" AND “IBD” AND "Microdamage accumulation" AND "Human" |
| "Bisphosphonate" AND "Osteoporosis" AND “IBD” AND "Mineral matrix ratio" AND "Human" |
| "Bisphosphonate" AND "Osteoporosis" AND “IBD” AND "Nanoindentation" AND "Human" AND “Clinical trial” |
| "Bisphosphonate" AND "Osteoporosis" AND “IBD” AND "Pentosidine" AND "Human" |
| "Bisphosphonate" AND "Osteoporosis" AND “IBD” AND "Bone loss" AND "Human" AND “Clinical trial” |
| "Bisphosphonate" AND "Osteoporosis" AND “IBD” AND "carbonate to amide ratio" AND "Human" |
| "Bisphosphonate" AND "Osteoporosis" AND “IBD” AND "Crystallinity" AND "Human" AND “Clinical trial” |
| "Bisphosphonate" AND "osteoporosis" AND “IBD” AND "Enzymatic to non-enzymatic cross-linking ratio" AND "Human" |
| "Bisphosphonate" AND "Osteoporosis" AND “IBD”AND "carbonate to phosphate ratio" AND "Human" |
| "Bisphosphonate" AND "Osteoporosis" AND “Arthritis” AND "Advanced glycation end products (AGEs)" AND "Human" |
| "Bisphosphonate" AND "Osteoporosis" AND “CKD” AND "Advanced glycation end products (AGEs)" AND "Human" |
| "Bisphosphonate" AND "Osteoporosis" AND “DIABETES” AND "Advanced glycation end products (AGEs)" AND "Human" |
| "Bisphosphonate" AND "Osteoporosis" AND “GIO” AND "Advanced glycation end products (AGEs)" AND "Human" |
| "Bisphosphonate" AND "Osteoporosis" AND “Hypertension” AND "Advanced glycation end products (AGEs)" AND "Human" |
| "Bisphosphonate" AND "Osteoporosis" AND “IBD” AND "Advanced glycation end products (AGEs)" AND "Human" |
| "Denosumab" AND “Arthritis” AND "Osteoporosis" AND "Collagen maturity" AND "Human" |
| "Denosumab" AND “CKD”AND "Osteoporosis" AND "Collagen maturity" AND "Human" |
| "Denosumab" AND “DIABETES”AND "Osteoporosis" AND "Collagen maturity" AND "Human" |
| "Denosumab" AND “GIO”AND "Osteoporosis" AND "Collagen maturity" AND "Human" |
| "Denosumab" AND “Hypertension” AND "Osteoporosis" AND "Collagen maturity" AND "Human" |
| "Denosumab" AND “IBD”AND "Osteoporosis" AND "Collagen maturity" AND "Human" |
| "Denosumab" AND "Osteoporosis" AND “Arthritis” AND "FEA" AND "Human" AND "Clinical trial" |
| "Denosumab" AND "Osteoporosis" AND “Arthritis” AND "Homocysteine" AND "Human" AND "Clinical trial" |
| "Denosumab" AND "Osteoporosis" AND “CKD”AND "FEA" AND "Human" AND "Clinical trial" |
| "Denosumab" AND "Osteoporosis" AND “CKD”AND "Homocysteine" AND "Human" AND "Clinical trial" |
| "Denosumab" AND "Osteoporosis" AND “DIABETES”AND "FEA" AND "Human" AND "Clinical trial" |
| "Denosumab" AND "Osteoporosis" AND “DIABETES”AND "Homocysteine" AND "Human" AND "Clinical trial" |
| "Denosumab" AND "Osteoporosis" AND “GIO”AND "FEA" AND "Human" AND "Clinical trial" |
| "Denosumab" AND "Osteoporosis" AND “GIO”AND "Homocysteine" AND "Human" AND "Clinical trial" |
| "Denosumab" AND "Osteoporosis" AND “Hypertension” AND "FEA" AND "Human" AND "Clinical trial" |
| "Denosumab" AND "Osteoporosis" AND “Hypertension” AND "Homocysteine" AND "Human" AND "Clinical trial" |
| "Denosumab" AND "Osteoporosis" AND “IBD”AND "FEA" AND "Human" AND "Clinical trial" |
| "Denosumab" AND "Osteoporosis" AND “IBD”AND "Homocysteine" AND "Human" AND "Clinical trial" |
| "Denosumab" AND "Osteoporosis" AND “Arthritis” AND "3 point bending" AND "Human" AND "Clinical trial" |
| "Denosumab" AND "Osteoporosis" AND “Arthritis” AND "Degree of mineralization" AND "Human" |
| "Denosumab" AND "Osteoporosis" AND “Arthritis” AND "Heterogeneity index" AND "Human" |
| "Denosumab" AND "Osteoporosis" AND “Arthritis” AND "Microdamage accumulation" AND "Human" |
| "Denosumab" AND "Osteoporosis" AND “Arthritis” AND "Mineral matrix ratio" AND "Human" |
| "Denosumab" AND "Osteoporosis" AND “Arthritis” AND "Nanoindentation" AND "Human" AND “Clinical trial” |
| "Denosumab" AND "Osteoporosis" AND “Arthritis” AND "Pentosidine" AND "Human" |
| "Denosumab" AND "Osteoporosis" AND “Arthritis” AND "Bone loss" AND "Human" AND “Clinical trial” |
| "Denosumab" AND "Osteoporosis" AND “Arthritis” AND "carbonate to amide ratio" AND "Human" |
| "Denosumab" AND "Osteoporosis" AND “Arthritis” AND "Crystallinity" AND "Human" AND “Clinical trial” |
| "Denosumab" AND "osteoporosis" AND “Arthritis” AND "Enzymatic to non-enzymatic cross-linking ratio" AND "Human" |
| "Denosumab" AND "Osteoporosis" AND “Arthritis” AND "carbonate to phosphate ratio" AND "Human" |
| "Denosumab" AND "Osteoporosis" AND “CKD” AND "3 point bending" AND "Human" AND "Clinical trial" |
| "Denosumab" AND "Osteoporosis" AND “CKD” AND "Degree of mineralization" AND "Human" |
| "Denosumab" AND "Osteoporosis" AND “CKD” AND "Heterogeneity index" AND "Human" |
| "Denosumab" AND "Osteoporosis" AND “CKD” AND "Microdamage accumulation" AND "Human" |
| "Denosumab" AND "Osteoporosis" AND “CKD” AND "Mineral matrix ratio" AND "Human" |
| "Denosumab" AND "Osteoporosis" AND “CKD” AND "Nanoindentation" AND "Human" AND “Clinical trial” |
| "Denosumab" AND "Osteoporosis" AND “CKD” AND "Pentosidine" AND "Human" |
| "Denosumab" AND "Osteoporosis" AND “CKD” AND "Bone loss" AND "Human" AND “Clinical trial” |
| "Denosumab" AND "Osteoporosis" AND “CKD” AND "carbonate to amide ratio" AND "Human" |
| "Denosumab" AND "Osteoporosis" AND “CKD” AND "Crystallinity" AND "Human" AND “Clinical trial” |
| "Denosumab" AND "osteoporosis" AND “CKD” AND "Enzymatic to non-enzymatic cross-linking ratio" AND "Human" |
| "Denosumab" AND "Osteoporosis" AND “CKD”AND "carbonate to phosphate ratio" AND "Human" |
| "Denosumab" AND "Osteoporosis" AND “DIABETES” AND "3 point bending" AND "Human" AND "Clinical trial" |
| "Denosumab" AND "Osteoporosis" AND “DIABETES” AND "Degree of mineralization" AND "Human" |
| "Denosumab" AND "Osteoporosis" AND “DIABETES” AND "Heterogeneity index" AND "Human" |
| "Denosumab" AND "Osteoporosis" AND “DIABETES” AND "Microdamage accumulation" AND "Human" |
| "Denosumab" AND "Osteoporosis" AND “DIABETES” AND "Mineral matrix ratio" AND "Human" |
| "Denosumab" AND "Osteoporosis" AND “DIABETES” AND "Nanoindentation" AND "Human" AND “Clinical trial” |
| "Denosumab" AND "Osteoporosis" AND “DIABETES” AND "Pentosidine" AND "Human" |
| "Denosumab" AND "Osteoporosis" AND “DIABETES” AND "Bone loss" AND "Human" AND “Clinical trial” |
| "Denosumab" AND "Osteoporosis" AND “DIABETES” AND "carbonate to amide ratio" AND "Human" |
| "Denosumab" AND "Osteoporosis" AND “DIABETES” AND "Crystallinity" AND "Human" AND “Clinical trial” |
| "Denosumab" AND "osteoporosis" AND “DIABETES” AND "Enzymatic to non-enzymatic cross-linking ratio" AND "Human" |
| "Denosumab" AND "Osteoporosis" AND “DIABETES”AND "carbonate to phosphate ratio" AND "Human" |
| "Denosumab" AND "Osteoporosis" AND “GIO” AND “3 point bending" AND "Human" AND "Clinical trial" |
| "Denosumab" AND "Osteoporosis" AND “GIO” AND “Degree of mineralization" AND "Human" |
| "Denosumab" AND "Osteoporosis" AND “GIO” AND "Heterogeneity index" AND "Human" |
| "Denosumab" AND "Osteoporosis" AND “GIO” AND "Microdamage accumulation" AND "Human" |
| "Denosumab" AND "Osteoporosis" AND “GIO” AND "Mineral matrix ratio" AND "Human" |
| "Denosumab" AND "Osteoporosis" AND “GIO” AND "Nanoindentation" AND "Human" AND “Clinical trial” |
| "Denosumab" AND "Osteoporosis" AND “GIO” AND "Pentosidine" AND "Human" |
| "Denosumab" AND "Osteoporosis" AND “GIO” AND "Bone loss" AND "Human" AND “Clinical trial” |
| "Denosumab" AND "Osteoporosis" AND “GIO” AND "carbonate to amide ratio" AND "Human" |
| "Denosumab" AND "Osteoporosis" AND “GIO” AND "Crystallinity" AND "Human" AND “Clinical trial” |
| "Denosumab" AND "osteoporosis" AND “GIO” AND "Enzymatic to non-enzymatic cross-linking ratio" AND "Human" |
| "Denosumab" AND "Osteoporosis" AND “GIO”AND "carbonate to phosphate ratio" AND "Human" |
| "Denosumab" AND "Osteoporosis" AND “Hypertension” AND "3 point bending" AND "Human" AND "Clinical trial" |
| "Denosumab" AND "Osteoporosis" AND “Hypertension” AND "Degree of mineralization" AND "Human" |
| "Denosumab" AND "Osteoporosis" AND “Hypertension” AND "Heterogeneity index" AND "Human" |
| "Denosumab" AND "Osteoporosis" AND “Hypertension” AND "Microdamage accumulation" AND "Human" |
| "Denosumab" AND "Osteoporosis" AND “Hypertension” AND "Mineral matrix ratio" AND "Human" |
| "Denosumab" AND "Osteoporosis" AND “Hypertension” AND "Nanoindentation" AND "Human" AND “Clinical trial” |
| "Denosumab" AND "Osteoporosis" AND “Hypertension” AND "Pentosidine" AND "Human" |
| "Denosumab" AND "Osteoporosis" AND “Hypertension” AND "Bone loss" AND "Human" AND “Clinical trial” |
| "Denosumab" AND "Osteoporosis" AND “Hypertension” AND "carbonate to amide ratio" AND "Human" |
| "Denosumab" AND "Osteoporosis" AND “Hypertension” AND "Crystallinity" AND "Human" AND “Clinical trial” |
| "Denosumab" AND "osteoporosis" AND “Hypertension” AND "Enzymatic to non-enzymatic cross-linking ratio" AND "Human" |
| "Denosumab" AND "Osteoporosis" AND “Hypertension” AND "carbonate to phosphate ratio" AND "Human" |
| "Denosumab" AND "Osteoporosis" AND “IBD” AND "3 point bending" AND "Human" AND "Clinical trial" |
| "Denosumab" AND "Osteoporosis" AND “IBD” AND "Degree of mineralization" AND "Human" |
| "Denosumab" AND "Osteoporosis" AND “IBD” AND "Heterogeneity index" AND "Human" |
| "Denosumab" AND "Osteoporosis" AND “IBD” AND "Microdamage accumulation" AND "Human" |
| "Denosumab" AND "Osteoporosis" AND “IBD” AND "Mineral matrix ratio" AND "Human" |
| "Denosumab" AND "Osteoporosis" AND “IBD” AND "Nanoindentation" AND "Human" AND “Clinical trial” |
| "Denosumab" AND "Osteoporosis" AND “IBD” AND "Pentosidine" AND "Human" |
| "Denosumab" AND "Osteoporosis" AND “IBD” AND "Bone loss" AND "Human" AND “Clinical trial” |
| "Denosumab" AND "Osteoporosis" AND “IBD” AND "carbonate to amide ratio" AND "Human" |
| "Denosumab" AND "Osteoporosis" AND “IBD” AND "Crystallinity" AND "Human" AND “Clinical trial” |
| "Denosumab" AND "osteoporosis" AND “IBD” AND "Enzymatic to non-enzymatic cross-linking ratio" AND "Human" |
| "Denosumab" AND "Osteoporosis" AND “IBD”AND "carbonate to phosphate ratio" AND "Human" |
| "Denosumab" AND "Osteoporosis" AND “Arthritis” AND "Advanced glycation end products (AGEs)" AND "Human" |
| "Denosumab" AND "Osteoporosis" AND “CKD” AND "Advanced glycation end products (AGEs)" AND "Human" |
| "Denosumab" AND "Osteoporosis" AND “DIABETES” AND "Advanced glycation end products (AGEs)" AND "Human" |
| "Denosumab" AND "Osteoporosis" AND “GIO” AND "Advanced glycation end products (AGEs)" AND "Human" |
| "Denosumab" AND "Osteoporosis" AND “Hypertension” AND "Advanced glycation end products (AGEs)" AND "Human" |
| "Denosumab" AND "Osteoporosis" AND “IBD” AND "Advanced glycation end products (AGEs)" AND "Human" |
| "Raloxifene" AND “Arthritis” AND "Osteoporosis" AND "Collagen maturity" AND "Human" |
| "Raloxifene" AND “CKD”AND "Osteoporosis" AND "Collagen maturity" AND "Human" |
| "Raloxifene" AND “DIABETES”AND "Osteoporosis" AND "Collagen maturity" AND "Human" |
| "Raloxifene" AND “GIO”AND "Osteoporosis" AND "Collagen maturity" AND "Human" |
| "Raloxifene" AND “Hypertension” AND "Osteoporosis" AND "Collagen maturity" AND "Human" |
| "Raloxifene" AND “IBD”AND "Osteoporosis" AND "Collagen maturity" AND "Human" |
| "Raloxifene" AND "Osteoporosis" AND “Arthritis” AND "FEA" AND "Human" AND "Clinical trial" |
| "Raloxifene" AND "Osteoporosis" AND “Arthritis” AND "Homocysteine" AND "Human" AND "Clinical trial" |
| "Raloxifene" AND "Osteoporosis" AND “CKD”AND "FEA" AND "Human" AND "Clinical trial" |
| "Raloxifene" AND "Osteoporosis" AND “CKD”AND "Homocysteine" AND "Human" AND "Clinical trial" |
| "Raloxifene" AND "Osteoporosis" AND “DIABETES”AND "FEA" AND "Human" AND "Clinical trial" |
| "Raloxifene" AND "Osteoporosis" AND “DIABETES”AND "Homocysteine" AND "Human" AND "Clinical trial" |
| "Raloxifene" AND "Osteoporosis" AND “GIO”AND "FEA" AND "Human" AND "Clinical trial" |
| "Raloxifene" AND "Osteoporosis" AND “GIO”AND "Homocysteine" AND "Human" AND "Clinical trial" |
| "Raloxifene" AND "Osteoporosis" AND “Hypertension” AND "FEA" AND "Human" AND "Clinical trial" |
| "Raloxifene" AND "Osteoporosis" AND “Hypertension” AND "Homocysteine" AND "Human" AND "Clinical trial" |
| "Raloxifene" AND "Osteoporosis" AND “IBD”AND "FEA" AND "Human" AND "Clinical trial" |
| "Raloxifene" AND "Osteoporosis" AND “IBD”AND "Homocysteine" AND "Human" AND "Clinical trial" |
| "Raloxifene" AND "Osteoporosis" AND “Arthritis” AND "3 point bending" AND "Human" AND "Clinical trial" |
| "Raloxifene" AND "Osteoporosis" AND “Arthritis” AND "Degree of mineralization" AND "Human" |
| "Raloxifene" AND "Osteoporosis" AND “Arthritis” AND "Heterogeneity index" AND "Human" |
| "Raloxifene" AND "Osteoporosis" AND “Arthritis” AND "Microdamage accumulation" AND "Human" |
| "Raloxifene" AND "Osteoporosis" AND “Arthritis” AND "Mineral matrix ratio" AND "Human" |
| "Raloxifene" AND "Osteoporosis" AND “Arthritis” AND "Nanoindentation" AND "Human" AND “Clinical trial” |
| "Raloxifene" AND "Osteoporosis" AND “Arthritis” AND "Pentosidine" AND "Human" |
| "Raloxifene" AND "Osteoporosis" AND “Arthritis” AND "Bone loss" AND "Human" AND “Clinical trial” |
| "Raloxifene" AND "Osteoporosis" AND “Arthritis” AND "carbonate to amide ratio" AND "Human" |
| "Raloxifene" AND "Osteoporosis" AND “Arthritis” AND "Crystallinity" AND "Human" AND “Clinical trial” |
| "Raloxifene" AND "osteoporosis" AND “Arthritis” AND "Enzymatic to non-enzymatic cross-linking ratio" AND "Human" |
| "Raloxifene" AND "Osteoporosis" AND “Arthritis” AND "carbonate to phosphate ratio" AND "Human" |
| "Raloxifene" AND "Osteoporosis" AND “CKD” AND "3 point bending" AND "Human" AND "Clinical trial" |
| "Raloxifene" AND "Osteoporosis" AND “CKD” AND "Degree of mineralization" AND "Human" |
| "Raloxifene" AND "Osteoporosis" AND “CKD” AND "Heterogeneity index" AND "Human" |
| "Raloxifene" AND "Osteoporosis" AND “CKD” AND "Microdamage accumulation" AND "Human" |
| "Raloxifene" AND "Osteoporosis" AND “CKD” AND "Mineral matrix ratio" AND "Human" |
| "Raloxifene" AND "Osteoporosis" AND “CKD” AND "Nanoindentation" AND "Human" AND “Clinical trial” |
| "Raloxifene" AND "Osteoporosis" AND “CKD” AND "Pentosidine" AND "Human" |
| "Raloxifene" AND "Osteoporosis" AND “CKD” AND "Bone loss" AND "Human" AND “Clinical trial” |
| "Raloxifene" AND "Osteoporosis" AND “CKD” AND "carbonate to amide ratio" AND "Human" |
| "Raloxifene" AND "Osteoporosis" AND “CKD” AND "Crystallinity" AND "Human" AND “Clinical trial” |
| "Raloxifene" AND "osteoporosis" AND “CKD” AND "Enzymatic to non-enzymatic cross-linking ratio" AND "Human" |
| "Raloxifene" AND "Osteoporosis" AND “CKD”AND "carbonate to phosphate ratio" AND "Human" |
| "Raloxifene" AND "Osteoporosis" AND “DIABETES” AND "3 point bending" AND "Human" AND "Clinical trial" |
| "Raloxifene" AND "Osteoporosis" AND “DIABETES” AND "Degree of mineralization" AND "Human" |
| "Raloxifene" AND "Osteoporosis" AND “DIABETES” AND "Heterogeneity index" AND "Human" |
| "Raloxifene" AND "Osteoporosis" AND “DIABETES” AND "Microdamage accumulation" AND "Human" |
| "Raloxifene" AND "Osteoporosis" AND “DIABETES” AND "Mineral matrix ratio" AND "Human" |
| "Raloxifene" AND "Osteoporosis" AND “DIABETES” AND "Nanoindentation" AND "Human" AND “Clinical trial” |
| "Raloxifene" AND "Osteoporosis" AND “DIABETES” AND "Pentosidine" AND "Human" |
| "Raloxifene" AND "Osteoporosis" AND “DIABETES” AND "Bone loss" AND "Human" AND “Clinical trial” |
| "Raloxifene" AND "Osteoporosis" AND “DIABETES” AND "carbonate to amide ratio" AND "Human" |
| "Raloxifene" AND "Osteoporosis" AND “DIABETES” AND "Crystallinity" AND "Human" AND “Clinical trial” |
| "Raloxifene" AND "osteoporosis" AND “DIABETES” AND "Enzymatic to non-enzymatic cross-linking ratio" AND "Human" |
| "Raloxifene" AND "Osteoporosis" AND “DIABETES”AND "carbonate to phosphate ratio" AND "Human" |
| "Raloxifene" AND "Osteoporosis" AND “GIO” AND "3 point bending" AND "Human" AND "Clinical trial" |
| "Raloxifene" AND "Osteoporosis" AND “GIO” AND "Degree of mineralization" AND "Human" |
| "Raloxifene" AND "Osteoporosis" AND “GIO” AND "Heterogeneity index" AND "Human" |
| "Raloxifene" AND "Osteoporosis" AND “GIO” AND "Microdamage accumulation" AND "Human" |
| "Raloxifene" AND "Osteoporosis" AND “GIO” AND "Mineral matrix ratio" AND "Human" |
| "Raloxifene" AND "Osteoporosis" AND “GIO” AND "Nanoindentation" AND "Human" AND “Clinical trial” |
| "Raloxifene" AND "Osteoporosis" AND “GIO” AND "Pentosidine" AND "Human" |
| "Raloxifene" AND "Osteoporosis" AND “GIO” AND "Bone loss" AND "Human" AND “Clinical trial” |
| "Raloxifene" AND "Osteoporosis" AND “GIO” AND "carbonate to amide ratio" AND "Human" |
| "Raloxifene" AND "Osteoporosis" AND “GIO” AND "Crystallinity" AND "Human" AND “Clinical trial” |
| "Raloxifene" AND "osteoporosis" AND “GIO” AND "Enzymatic to non-enzymatic cross-linking ratio" AND "Human" |
| "Raloxifene" AND "Osteoporosis" AND “GIO”AND "carbonate to phosphate ratio" AND "Human" |
| "Raloxifene" AND "Osteoporosis" AND “Hypertension” AND "3 point bending" AND "Human" AND "Clinical trial" |
| "Raloxifene" AND "Osteoporosis" AND “Hypertension” AND "Degree of mineralization" AND "Human" |
| "Raloxifene" AND "Osteoporosis" AND “Hypertension” AND "Heterogeneity index" AND "Human" |
| "Raloxifene" AND "Osteoporosis" AND “Hypertension” AND "Microdamage accumulation" AND "Human" |
| "Raloxifene" AND "Osteoporosis" AND “Hypertension” AND "Mineral matrix ratio" AND "Human" |
| "Raloxifene" AND "Osteoporosis" AND “Hypertension” AND "Nanoindentation" AND "Human" AND “Clinical trial” |
| "Raloxifene" AND "Osteoporosis" AND “Hypertension” AND "Pentosidine" AND "Human" |
| "Raloxifene" AND "Osteoporosis" AND “Hypertension” AND "Bone loss" AND "Human" AND “Clinical trial” |
| "Raloxifene" AND "Osteoporosis" AND “Hypertension” AND "carbonate to amide ratio" AND "Human" |
| "Raloxifene" AND "Osteoporosis" AND “Hypertension” AND "Crystallinity" AND "Human" AND “Clinical trial” |
| "Raloxifene" AND "osteoporosis" AND “Hypertension” AND "Enzymatic to non-enzymatic cross-linking ratio" AND "Human" |
| "Raloxifene" AND "Osteoporosis" AND “Hypertension” AND "carbonate to phosphate ratio" AND "Human" |
| "Raloxifene" AND "Osteoporosis" AND “IBD” AND "3 point bending" AND "Human" AND "Clinical trial" |
| "Raloxifene" AND "Osteoporosis" AND “IBD” AND "Degree of mineralization" AND "Human" |
| "Raloxifene" AND "Osteoporosis" AND “IBD” AND "Heterogeneity index" AND "Human" |
| "Raloxifene" AND "Osteoporosis" AND “IBD” AND "Microdamage accumulation" AND "Human" |
| "Raloxifene" AND "Osteoporosis" AND “IBD” AND "Mineral matrix ratio" AND "Human" |
| "Raloxifene" AND "Osteoporosis" AND “IBD” AND "Nanoindentation" AND "Human" AND “Clinical trial” |
| "Raloxifene" AND "Osteoporosis" AND “IBD” AND "Pentosidine" AND "Human" |
| "Raloxifene" AND "Osteoporosis" AND “IBD” AND "Bone loss" AND "Human" AND “Clinical trial” |
| "Raloxifene" AND "Osteoporosis" AND “IBD” AND "carbonate to amide ratio" AND "Human" |
| "Raloxifene" AND "Osteoporosis" AND “IBD” AND "Crystallinity" AND "Human" AND “Clinical trial” |
| "Raloxifene" AND "osteoporosis" AND “IBD” AND "Enzymatic to non-enzymatic cross-linking ratio" AND "Human" |
| "Raloxifene" AND "Osteoporosis" AND “IBD”AND "carbonate to phosphate ratio" AND "Human" |
| "Raloxifene"AND "Osteoporosis" AND “Arthritis” AND "Advanced glycation end products (AGEs)" AND "Human" |
| "Raloxifene"AND "Osteoporosis" AND “CKD” AND "Advanced glycation end products (AGEs)" AND "Human" |
| "Raloxifene"AND "Osteoporosis" AND “DIABETES” AND "Advanced glycation end products (AGEs)" AND "Human" |
| "Raloxifene"AND "Osteoporosis" AND “GIO” AND "Advanced glycation end products (AGEs)" AND "Human" |
| "Raloxifene"AND "Osteoporosis" AND “Hypertension” AND "Advanced glycation end products (AGEs)" AND "Human" |
| "Raloxifene"AND "Osteoporosis" AND “IBD” AND "Advanced glycation end products (AGEs)" AND "Human" |
| "Romosozumab" AND “Arthritis” AND "Osteoporosis" AND "Collagen maturity" AND "Human" |
| "Romosozumab" AND “CKD”AND "Osteoporosis" AND "Collagen maturity" AND "Human" |
| "Romosozumab" AND “DIABETES”AND "Osteoporosis" AND "Collagen maturity" AND "Human" |
| "Romosozumab" AND “GIO”AND "Osteoporosis" AND "Collagen maturity" AND "Human" |
| "Romosozumab" AND “Hypertension” AND "Osteoporosis" AND "Collagen maturity" AND "Human" |
| "Romosozumab" AND “IBD”AND "Osteoporosis" AND "Collagen maturity" AND "Human" |
| "Romosozumab" AND "Osteoporosis" AND “Arthritis” AND "FEA" AND "Human" AND "Clinical trial" |
| "Romosozumab" AND "Osteoporosis" AND “Arthritis” AND "Homocysteine" AND "Human" AND "Clinical trial" |
| "Romosozumab" AND "Osteoporosis" AND “CKD”AND "FEA" AND "Human" AND "Clinical trial" |
| "Romosozumab" AND "Osteoporosis" AND “CKD”AND "Homocysteine" AND "Human" AND "Clinical trial" |
| "Romosozumab" AND "Osteoporosis" AND “DIABETES”AND "FEA" AND "Human" AND "Clinical trial" |
| "Romosozumab" AND "Osteoporosis" AND “DIABETES”AND "Homocysteine" AND "Human" AND "Clinical trial" |
| "Romosozumab" AND "Osteoporosis" AND “GIO”AND "FEA" AND "Human" AND "Clinical trial" |
| "Romosozumab" AND "Osteoporosis" AND “GIO”AND "Homocysteine" AND "Human" AND "Clinical trial" |
| "Romosozumab" AND "Osteoporosis" AND “Hypertension” AND "FEA" AND "Human" AND "Clinical trial" |
| "Romosozumab" AND "Osteoporosis" AND “Hypertension” AND "Homo cysteine" AND "Human" AND "Clinical trial" |
| "Romosozumab" AND "Osteoporosis" AND “IBD”AND "FEA" AND "Human" AND "Clinical trial" |
| "Romosozumab" AND "Osteoporosis" AND “IBD”AND "Homo cysteine" AND "Human" AND "Clinical trial" |
| "Romosozumab" AND "Osteoporosis" AND “Arthritis” AND "3 point bending" AND "Human" AND "Clinical trial" |
| "Romosozumab" AND "Osteoporosis" AND “Arthritis” AND "Degree of mineralization" AND "Human" |
| "Romosozumab" AND "Osteoporosis" AND “Arthritis” AND "Heterogeneity index" AND "Human" |
| "Romosozumab" AND "Osteoporosis" AND “Arthritis” AND "Microdamage accumulation" AND "Human" |
| "Romosozumab" AND "Osteoporosis" AND “Arthritis” AND "Mineral matrix ratio" AND "Human" |
| "Romosozumab" AND "Osteoporosis" AND “Arthritis” AND "Nanoindentation" AND "Human" AND “Clinical trial” |
| "Romosozumab" AND "Osteoporosis" AND “Arthritis” AND "Pentosidine" AND "Human" |
| "Romosozumab" AND "Osteoporosis" AND “Arthritis” AND "Bone loss" AND "Human" AND “Clinical trial” |
| "Romosozumab" AND "Osteoporosis" AND “Arthritis” AND "carbonate to amide ratio" AND "Human" |
| "Romosozumab" AND "Osteoporosis" AND “Arthritis” AND "Crystallinity" AND "Human" AND “Clinical trial” |
| "Romosozumab" AND "osteoporosis" AND “Arthritis” AND "Enzymatic to non-enzymatic cross-linking ratio" AND "Human" |
| "Romosozumab" AND "Osteoporosis" AND “Arthritis” AND "carbonate to phosphate ratio" AND "Human" |
| "Romosozumab" AND "Osteoporosis" AND “CKD” AND "3 point bending" AND "Human" AND "Clinical trial" |
| "Romosozumab" AND "Osteoporosis" AND “CKD” AND "Degree of mineralization" AND "Human" |
| "Romosozumab" AND "Osteoporosis" AND “CKD” AND "Heterogeneity index" AND "Human" |
| "Romosozumab" AND "Osteoporosis" AND “CKD” AND "Microdamage accumulation" AND "Human" |
| "Romosozumab" AND "Osteoporosis" AND “CKD” AND "Mineral matrix ratio" AND "Human" |
| "Romosozumab" AND "Osteoporosis" AND “CKD” AND "Nanoindentation" AND "Human" AND “Clinical trial” |
| "Romosozumab" AND "Osteoporosis" AND “CKD” AND "Pentosidine" AND "Human" |
| "Romosozumab" AND "Osteoporosis" AND “CKD” AND "Bone loss" AND "Human" AND “Clinical trial” |
| "Romosozumab" AND "Osteoporosis" AND “CKD” AND "carbonate to amide ratio" AND "Human" |
| "Romosozumab" AND "Osteoporosis" AND “CKD” AND "Crystallinity" AND "Human" AND “Clinical trial” |
| "Romosozumab" AND "osteoporosis" AND “CKD” AND "Enzymatic to non-enzymatic cross-linking ratio" AND "Human" |
| "Romosozumab" AND "Osteoporosis" AND “CKD”AND "carbonate to phosphate ratio" AND "Human" |
| "Romosozumab" AND "Osteoporosis" AND “DIABETES” AND "3 point bending" AND "Human" AND "Clinical trial" |
| "Romosozumab" AND "Osteoporosis" AND “DIABETES” AND "Degree of mineralization" AND "Human" |
| "Romosozumab" AND "Osteoporosis" AND “DIABETES” AND "Heterogeneity index" AND "Human" |
| "Romosozumab" AND "Osteoporosis" AND “DIABETES” AND "Microdamage accumulation" AND "Human" |
| "Romosozumab" AND "Osteoporosis" AND “DIABETES” AND "Mineral matrix ratio" AND "Human" |
| "Romosozumab" AND "Osteoporosis" AND “DIABETES” AND "Nanoindentation" AND "Human" AND “Clinical trial” |
| "Romosozumab" AND "Osteoporosis" AND “DIABETES” AND "Pentosidine" AND "Human" |
| "Romosozumab" AND "Osteoporosis" AND “DIABETES” AND "Bone loss" AND "Human" AND “Clinical trial” |
| "Romosozumab" AND "Osteoporosis" AND “DIABETES” AND "carbonate to amide ratio" AND "Human" |
| "Romosozumab" AND "Osteoporosis" AND “DIABETES” AND "Crystallinity" AND "Human" AND “Clinical trial” |
| "Romosozumab" AND "osteoporosis" AND “DIABETES” AND "Enzymatic to non-enzymatic cross-linking ratio" AND "Human" |
| "Romosozumab" AND "Osteoporosis" AND “DIABETES”AND "carbonate to phosphate ratio" AND "Human" |
| "Romosozumab" AND "Osteoporosis" AND “GIO” AND "3 point bending" AND "Human" AND "Clinical trial" |
| "Romosozumab" AND "Osteoporosis" AND “GIO” AND "Degree of mineralization" AND "Human" |
| "Romosozumab" AND "Osteoporosis" AND “GIO” AND "Heterogeneity index" AND "Human" |
| "Romosozumab" AND "Osteoporosis" AND “GIO” AND "Microdamage accumulation" AND "Human" |
| "Romosozumab" AND "Osteoporosis" AND “GIO” AND "Mineral matrix ratio" AND "Human" |
| "Romosozumab" AND "Osteoporosis" AND “GIO” AND "Nanoindentation" AND "Human" AND “Clinical trial” |
| "Romosozumab" AND "Osteoporosis" AND “GIO” AND "Pentosidine" AND "Human" |
| "Romosozumab" AND "Osteoporosis" AND “GIO” AND "Bone loss" AND "Human" AND “Clinical trial” |
| "Romosozumab" AND "Osteoporosis" AND “GIO” AND "carbonate to amide ratio" AND "Human" |
| "Romosozumab" AND "Osteoporosis" AND “GIO” AND "Crystallinity" AND "Human" AND “Clinical trial” |
| "Romosozumab" AND "osteoporosis" AND “GIO” AND "Enzymatic to non-enzymatic cross-linking ratio" AND "Human" |
| "Romosozumab" AND "Osteoporosis" AND “GIO”AND "carbonate to phosphate ratio" AND "Human" |
| "Romosozumab" AND "Osteoporosis" AND “Hypertension” AND "3 point bending" AND "Human" AND "Clinical trial" |
| "Romosozumab" AND "Osteoporosis" AND “Hypertension” AND "Degree of mineralization" AND "Human" |
| "Romosozumab" AND "Osteoporosis" AND “Hypertension” AND "Heterogeneity index" AND "Human" |
| "Romosozumab" AND "Osteoporosis" AND “Hypertension” AND "Microdamage accumulation" AND "Human" |
| "Romosozumab" AND "Osteoporosis" AND “Hypertension” AND "Mineral matrix ratio" AND "Human" |
| "Romosozumab" AND "Osteoporosis" AND “Hypertension” AND "Nanoindentation" AND "Human" AND “Clinical trial” |
| "Romosozumab" AND "Osteoporosis" AND “Hypertension” AND "Pentosidine" AND "Human" |
| "Romosozumab" AND "Osteoporosis" AND “Hypertension” AND "Bone loss" AND "Human" AND “Clinical trial” |
| "Romosozumab" AND "Osteoporosis" AND “Hypertension” AND "carbonate to amide ratio" AND "Human" |
| "Romosozumab" AND "Osteoporosis" AND “Hypertension” AND "Crystallinity" AND "Human" AND “Clinical trial” |
| "Romosozumab" AND "osteoporosis" AND “Hypertension” AND "Enzymatic to non-enzymatic cross-linking ratio" AND "Human" |
| "Romosozumab" AND "Osteoporosis" AND “Hypertension” AND "carbonate to phosphate ratio" AND "Human" |
| "Romosozumab" AND "Osteoporosis" AND “IBD” AND "3 point bending" AND "Human" AND "Clinical trial" |
| "Romosozumab" AND "Osteoporosis" AND “IBD” AND "Degree of mineralization" AND "Human" |
| "Romosozumab" AND "Osteoporosis" AND “IBD” AND "Heterogeneity index" AND "Human" |
| "Romosozumab" AND "Osteoporosis" AND “IBD” AND "Microdamage accumulation" AND "Human" |
| "Romosozumab" AND "Osteoporosis" AND “IBD” AND "Mineral matrix ratio" AND "Human" |
| "Romosozumab" AND "Osteoporosis" AND “IBD” AND "Nanoindentation" AND "Human" AND “Clinical trial” |
| "Romosozumab" AND "Osteoporosis" AND “IBD” AND "Pentosidine" AND "Human" |
| "Romosozumab" AND "Osteoporosis" AND “IBD” AND "Bone loss" AND "Human" AND “Clinical trial” |
| "Romosozumab" AND "Osteoporosis" AND “IBD” AND "carbonate to amide ratio" AND "Human" |
| "Romosozumab" AND "Osteoporosis" AND “IBD” AND "Crystallinity" AND "Human" AND “Clinical trial” |
| "Romosozumab" AND "osteoporosis" AND “IBD” AND "Enzymatic to non-enzymatic cross-linking ratio" AND "Human" |
| "Romosozumab" AND "Osteoporosis" AND “IBD”AND "carbonate to phosphate ratio" AND "Human" |
| "Romosozumab" AND "Osteoporosis" AND “Arthritis” AND "Advanced glycation end products (AGEs)" AND "Human" |
| "Romosozumab" AND "Osteoporosis" AND “CKD” AND "Advanced glycation end products (AGEs)" AND "Human" |
| "Romosozumab" AND "Osteoporosis" AND “DIABETES” AND "Advanced glycation end products (AGEs)" AND "Human" |
| "Romosozumab" AND "Osteoporosis" AND “GIO” AND "Advanced glycation end products (AGEs)" AND "Human" |
| "Romosozumab" AND "Osteoporosis" AND “Hypertension” AND "Advanced glycation end products (AGEs)" AND "Human" |
| "Romosozumab" AND "Osteoporosis" AND “IBD” AND "Advanced glycation end products (AGEs)" AND "Human" |
| "Strontium ranelate" AND “Arthritis” AND "Osteoporosis" AND "Collagen maturity" AND "Human" |
| "Strontium ranelate" AND “CKD”AND "Osteoporosis" AND "Collagen maturity" AND "Human" |
| "Strontium ranelate" AND “DIABETES”AND "Osteoporosis" AND "Collagen maturity" AND "Human" |
| "Strontium ranelate" AND “GIO”AND "Osteoporosis" AND "Collagen maturity" AND "Human" |
| "Strontium ranelate" AND “Hypertension” AND "Osteoporosis" AND "Collagen maturity" AND "Human" |
| "Strontium ranelate" AND “IBD”AND "Osteoporosis" AND "Collagen maturity" AND "Human" |
| "Strontium ranelate" AND "Osteoporosis" AND “Arthritis” AND "FEA" AND "Human" AND "Clinical trial" |
| "Strontium ranelate" AND "Osteoporosis" AND “Arthritis” AND "Homocysteine" AND "Human" AND "Clinical trial" |
| "Strontium ranelate" AND "Osteoporosis" AND “CKD”AND "FEA" AND "Human" AND "Clinical trial" |
| "Strontium ranelate" AND "Osteoporosis" AND “CKD”AND "Homocysteine" AND "Human" AND "Clinical trial" |
| "Strontium ranelate" AND "Osteoporosis" AND “DIABETES”AND "FEA" AND "Human" AND "Clinical trial" |
| "Strontium ranelate" AND "Osteoporosis" AND “DIABETES”AND "Homocysteine" AND "Human" AND "Clinical trial" |
| "Strontium ranelate" AND "Osteoporosis" AND “GIO”AND "FEA" AND "Human" AND "Clinical trial" |
| "Strontium ranelate" AND "Osteoporosis" AND “GIO”AND "Homocysteine" AND "Human" AND "Clinical trial" |
| "Strontium ranelate" AND "Osteoporosis" AND “Hypertension” AND "Homocysteine" AND "Human" AND "Clinical trial" |
| "Strontium ranelate" AND "Osteoporosis" AND “Hypertension” AND "FEA" AND "Human" AND "Clinical trial" |
| "Strontium ranelate" AND "Osteoporosis" AND “IBD”AND "FEA" AND "Human" AND "Clinical trial" |
| "Strontium ranelate" AND "Osteoporosis" AND “IBD”AND "Homocysteine" AND "Human" AND "Clinical trial" |
| "Strontium ranelate" AND "Osteoporosis" AND “Arthritis” AND "3 point bending" AND "Human" AND "Clinical trial" |
| "Strontium ranelate" AND "Osteoporosis" AND “Arthritis” AND "Degree of mineralization" AND "Human" |
| "Strontium ranelate" AND "Osteoporosis" AND “Arthritis” AND "Heterogeneity index" AND "Human" |
| "Strontium ranelate" AND "Osteoporosis" AND “Arthritis” AND "Microdamage accumulation" AND "Human" |
| "Strontium ranelate" AND "Osteoporosis" AND “Arthritis” AND "Mineral matrix ratio" AND "Human" |
| "Strontium ranelate" AND "Osteoporosis" AND “Arthritis” AND "Nanoindentation" AND "Human" AND “Clinical trial” |
| "Strontium ranelate" AND "Osteoporosis" AND “Arthritis” AND "Pentosidine" AND "Human" |
| "Strontium ranelate" AND "Osteoporosis" AND “Arthritis” AND "Bone loss" AND "Human" AND “Clinical trial” |
| "Strontium ranelate" AND "Osteoporosis" AND “Arthritis” AND "carbonate to amide ratio" AND "Human" |
| "Strontium ranelate" AND "Osteoporosis" AND “Arthritis” AND "Crystallinity" AND "Human" AND “Clinical trial” |
| "Strontium ranelate" AND "osteoporosis" AND “Arthritis” AND "Enzymatic to non-enzymatic cross-linking ratio" AND "Human" |
| "Strontium ranelate" AND "Osteoporosis" AND “Arthritis” AND "carbonate to phosphate ratio" AND "Human" |
| "Strontium ranelate" AND "Osteoporosis" AND “CKD” AND "3 point bending" AND "Human" AND "Clinical trial" |
| "Strontium ranelate" AND "Osteoporosis" AND “CKD” AND "Degree of mineralization" AND "Human" |
| "Strontium ranelate" AND "Osteoporosis" AND “CKD” AND "Heterogeneity index" AND "Human" |
| "Strontium ranelate" AND "Osteoporosis" AND “CKD” AND "Microdamage accumulation" AND "Human" |
| "Strontium ranelate" AND "Osteoporosis" AND “CKD” AND "Mineral matrix ratio" AND "Human" |
| "Strontium ranelate" AND "Osteoporosis" AND “CKD” AND "Nanoindentation" AND "Human" AND “Clinical trial” |
| "Strontium ranelate" AND "Osteoporosis" AND “CKD” AND "Pentosidine" AND "Human" |
| "Strontium ranelate" AND "Osteoporosis" AND “CKD” AND "Bone loss" AND "Human" AND “Clinical trial” |
| "Strontium ranelate" AND "Osteoporosis" AND “CKD” AND "carbonate to amide ratio" AND "Human" |
| "Strontium ranelate" AND "Osteoporosis" AND “CKD” AND "Crystallinity" AND "Human" AND “Clinical trial” |
| "Strontium ranelate" AND "osteoporosis" AND “CKD” AND "Enzymatic to non-enzymatic cross-linking ratio" AND "Human" |
| "Strontium ranelate" AND "Osteoporosis" AND “CKD”AND "carbonate to phosphate ratio" AND "Human" |
| "Strontium ranelate" AND "Osteoporosis" AND “DIABETES” AND "3 point bending" AND "Human" AND "Clinical trial" |
| "Strontium ranelate" AND "Osteoporosis" AND “DIABETES” AND "Degree of mineralization" AND "Human" |
| "Strontium ranelate" AND "Osteoporosis" AND “DIABETES” AND "Heterogeneity index" AND "Human" |
| "Strontium ranelate" AND "Osteoporosis" AND “DIABETES” AND "Microdamage accumulation" AND "Human" |
| "Strontium ranelate" AND "Osteoporosis" AND “DIABETES” AND "Mineral matrix ratio" AND "Human" |
| "Strontium ranelate" AND "Osteoporosis" AND “DIABETES” AND "Nanoindentation" AND "Human" AND “Clinical trial” |
| "Strontium ranelate" AND "Osteoporosis" AND “DIABETES” AND "Pentosidine" AND "Human" |
| "Strontium ranelate" AND "Osteoporosis" AND “DIABETES” AND "Bone loss" AND "Human" AND “Clinical trial” |
| "Strontium ranelate" AND "Osteoporosis" AND “DIABETES” AND "carbonate to amide ratio" AND "Human" |
| "Strontium ranelate" AND "Osteoporosis" AND “DIABETES” AND "Crystallinity" AND "Human" AND “Clinical trial” |
| "Strontium ranelate" AND "osteoporosis" AND “DIABETES” AND "Enzymatic to non-enzymatic cross-linking ratio" AND "Human" |
| "Strontium ranelate" AND "Osteoporosis" AND “DIABETES”AND "carbonate to phosphate ratio" AND "Human" |
| "Strontium ranelate" AND "Osteoporosis" AND “GIO” AND "3 point bending" AND "Human" AND "Clinical trial" |
| "Strontium ranelate" AND "Osteoporosis" AND “GIO” AND "Degree of mineralization" AND "Human" |
| "Strontium ranelate" AND "Osteoporosis" AND “GIO” AND "Heterogeneity index" AND "Human" |
| "Strontium ranelate" AND "Osteoporosis" AND “GIO” AND "Microdamage accumulation" AND "Human" |
| "Strontium ranelate" AND "Osteoporosis" AND “GIO” AND "Mineral matrix ratio" AND "Human" |
| "Strontium ranelate" AND "Osteoporosis" AND “GIO” AND "Nanoindentation" AND "Human" AND “Clinical trial” |
| "Strontium ranelate" AND "Osteoporosis" AND “GIO” AND "Pentosidine" AND "Human" |
| "Strontium ranelate" AND "Osteoporosis" AND “GIO” AND "Bone loss" AND "Human" AND “Clinical trial” |
| "Strontium ranelate" AND "Osteoporosis" AND “GIO” AND "carbonate to amide ratio" AND "Human" |
| "Strontium ranelate" AND "Osteoporosis" AND “GIO” AND "Crystallinity" AND "Human" AND “Clinical trial” |
| "Strontium ranelate" AND "osteoporosis" AND “GIO” AND "Enzymatic to non-enzymatic cross-linking ratio" AND "Human" |
| "Strontium ranelate" AND "Osteoporosis" AND “GIO”AND "carbonate to phosphate ratio" AND "Human" |
| "Strontium ranelate" AND "Osteoporosis" AND “Hypertension” AND "Mineral matrix ratio" AND "Human" |
| "Strontium ranelate" AND "Osteoporosis" AND “Hypertension” AND "Microdamage accumulation" AND "Human" |
| "Strontium ranelate" AND "Osteoporosis" AND “Hypertension” AND "Heterogeneity index" AND "Human" |
| "Strontium ranelate" AND "Osteoporosis" AND “Hypertension” AND "Pentosidine" AND "Human" |
| "Strontium ranelate" AND "Osteoporosis" AND “Hypertension” AND "Degree of mineralization" AND "Human" |
| "Strontium ranelate" AND "Osteoporosis" AND “Hypertension” AND "3 point bending" AND "Human" AND "Clinical trial" |
| "Strontium ranelate" AND "Osteoporosis" AND “Hypertension” AND "Nanoindentation" AND "Human" AND “Clinical trial” |
| "Strontium ranelate" AND "Osteoporosis" AND “Hypertension” AND "Bone loss" AND "Human" AND “Clinical trial” |
| "Strontium ranelate" AND "Osteoporosis" AND “Hypertension” AND "carbonate to amide ratio" AND "Human" |
| "Strontium ranelate" AND "osteoporosis" AND “Hypertension” AND "Enzymatic to non-enzymatic cross-linking ratio" AND "Human" |
| "Strontium ranelate" AND "Osteoporosis" AND “Hypertension” AND "carbonate to phosphate ratio" AND "Human" |
| "Strontium ranelate" AND "Osteoporosis" AND “IBD” AND "3 point bending" AND "Human" AND "Clinical trial" |
| "Strontium ranelate" AND "Osteoporosis" AND “IBD” AND "Degree of mineralization" AND "Human" |
| "Strontium ranelate" AND "Osteoporosis" AND “IBD” AND "Heterogeneity index" AND "Human" |
| "Strontium ranelate" AND "Osteoporosis" AND “IBD” AND "Microdamage accumulation" AND "Human" |
| "Strontium ranelate" AND "Osteoporosis" AND “IBD” AND "Mineral matrix ratio" AND "Human" |
| "Strontium ranelate" AND "Osteoporosis" AND “IBD” AND "Nanoindentation" AND "Human" AND “Clinical trial” |
| "Strontium ranelate" AND "Osteoporosis" AND “IBD” AND "Nanoindentation" AND "Human" AND “Clinical trial” |
| "Strontium ranelate" AND "Osteoporosis" AND “IBD” AND "Pentosidine" AND "Human" |
| "Strontium ranelate" AND "Osteoporosis" AND “IBD” AND "Bone loss" AND "Human" AND “Clinical trial” |
| "Strontium ranelate" AND "Osteoporosis" AND “IBD” AND "carbonate to amide ratio" AND "Human" |
| "Strontium ranelate" AND "osteoporosis" AND “IBD” AND "Enzymatic to non-enzymatic cross-linking ratio" AND "Human" |
| "Strontium ranelate" AND "Osteoporosis" AND “IBD”AND "carbonate to phosphate ratio" AND "Human" |
| "Strontium ranelate" AND "Osteoporosis" AND “Arthritis” AND "Advanced glycation end products (AGEs)" AND "Human" |
| "Strontium ranelate" AND "Osteoporosis" AND “CKD” AND "Advanced glycation end products (AGEs)" AND "Human" |
| "Strontium ranelate" AND "Osteoporosis" AND “DIABETES” AND "Advanced glycation end products (AGEs)" AND "Human" |
| "Strontium ranelate" AND "Osteoporosis" AND “GIO” AND "Advanced glycation end products (AGEs)" AND "Human" |
| "Teriparatide" AND “Arthritis” AND "Osteoporosis" AND "Collagen maturity" AND "Human" |
| "Teriparatide" AND “Arthritis” AND "Osteoporosis" AND "Collagen maturity" AND "Human" |
| "Teriparatide" AND “CKD”AND "Osteoporosis" AND "Collagen maturity" AND "Human" |
| "Teriparatide" AND “DIABETES”AND "Osteoporosis" AND "Collagen maturity" AND "Human" |
| "Teriparatide" AND “GIO”AND "Osteoporosis" AND "Collagen maturity" AND "Human" |
| "Teriparatide" AND “Hypertension” AND "Osteoporosis" AND "Collagen maturity" AND "Human" |
| "Teriparatide" AND “IBD”AND "Osteoporosis" AND "Collagen maturity" AND "Human" |
| "Teriparatide" AND "Osteoporosis" AND “Arthritis” AND "FEA" AND "Human" AND "Clinical trial" |
| "Teriparatide" AND "Osteoporosis" AND “Arthritis” AND "FEA" AND "Human" AND "Clinical trial" |
| "Teriparatide" AND "Osteoporosis" AND “Arthritis” AND "Homocysteine" AND "Human" AND "Clinical trial" |
| "Teriparatide" AND "Osteoporosis" AND “Arthritis” AND "Homocysteine" AND "Human" AND "Clinical trial" |
| "Teriparatide" AND "Osteoporosis" AND “CKD”AND "FEA" AND "Human" AND "Clinical trial" |
| "Teriparatide" AND "Osteoporosis" AND “CKD”AND "Homocysteine" AND "Human" AND "Clinical trial" |
| "Teriparatide" AND "Osteoporosis" AND “DIABETES”AND "FEA" AND "Human" AND "Clinical trial" |
| "Teriparatide" AND "Osteoporosis" AND “DIABETES”AND "Homocysteine" AND "Human" AND "Clinical trial" |
| "Teriparatide" AND "Osteoporosis" AND “GIO”AND "FEA" AND "Human" AND "Clinical trial" |
| "Teriparatide" AND "Osteoporosis" AND “GIO”AND "Homocysteine" AND "Human" AND "Clinical trial" |
| "Teriparatide" AND "Osteoporosis" AND “Hypertension” AND "FEA" AND "Human" AND "Clinical trial" |
| "Teriparatide" AND "Osteoporosis" AND “Hypertension” AND "Homocysteine" AND "Human" AND "Clinical trial" |
| "Teriparatide" AND "Osteoporosis" AND “IBD”AND "FEA" AND "Human" AND "Clinical trial" |
| "Teriparatide" AND "Osteoporosis" AND “IBD”AND "Homocysteine" AND "Human" AND "Clinical trial" |
| "Teriparatide" AND "Osteoporosis" AND “Arthritis” AND "3 point bending" AND "Human" AND "Clinical trial" |
| "Teriparatide" AND "Osteoporosis" AND “Arthritis” AND "3 point bending" AND "Human" AND "Clinical trial" |
| "Teriparatide" AND "Osteoporosis" AND “Arthritis” AND "Degree of mineralization" AND "Human" |
| "Teriparatide" AND "Osteoporosis" AND “Arthritis” AND "Degree of mineralization" AND "Human" |
| "Teriparatide" AND "Osteoporosis" AND “Arthritis” AND "Heterogeneity index" AND "Human" |
| "Teriparatide" AND "Osteoporosis" AND “Arthritis” AND "Heterogeneity index" AND "Human" |
| "Teriparatide" AND "Osteoporosis" AND “Arthritis” AND "Microdamage accumulation" AND "Human" |
| "Teriparatide" AND "Osteoporosis" AND “Arthritis” AND "Microdamage accumulation" AND "Human" |
| "Teriparatide" AND "Osteoporosis" AND “Arthritis” AND "Mineral matrix ratio" AND "Human" |
| "Teriparatide" AND "Osteoporosis" AND “Arthritis” AND "Mineral matrix ratio" AND "Human" |
| "Teriparatide" AND "Osteoporosis" AND “Arthritis” AND "Nanoindentation" AND "Human" AND “Clinical trial” |
| "Teriparatide" AND "Osteoporosis" AND “Arthritis” AND "Nanoindentation" AND "Human" AND “Clinical trial” |
| "Teriparatide" AND "Osteoporosis" AND “Arthritis” AND "Pentosidine" AND "Human" |
| "Teriparatide" AND "Osteoporosis" AND “Arthritis” AND "Pentosidine" AND "Human" |
| "Teriparatide" AND "Osteoporosis" AND “Arthritis” AND "Bone loss" AND "Human" AND “Clinical trial” |
| "Teriparatide" AND "Osteoporosis" AND “Arthritis” AND "Bone loss" AND "Human" AND “Clinical trial” |
| "Teriparatide" AND "Osteoporosis" AND “Arthritis” AND "carbonate to amide ratio" AND "Human" |
| "Teriparatide" AND "Osteoporosis" AND “Arthritis” AND "carbonate to amide ratio" AND "Human" |
| "Teriparatide" AND "Osteoporosis" AND “Arthritis” AND "Crystallinity" AND "Human" AND “Clinical trial” |
| "Teriparatide" AND "Osteoporosis" AND “Arthritis” AND "Crystallinity" AND "Human" AND “Clinical trial” |
| "Teriparatide" AND "osteoporosis" AND “Arthritis” AND "Enzymatic to non-enzymatic cross-linking ratio" AND "Human" |
| "Teriparatide" AND "osteoporosis" AND “Arthritis” AND "Enzymatic to non-enzymatic cross-linking ratio" AND "Human" |
| "Teriparatide" AND "Osteoporosis" AND “Arthritis” AND "carbonate to phosphate ratio" AND "Human" |
| "Teriparatide" AND "Osteoporosis" AND “Arthritis” AND "carbonate to phosphate ratio" AND "Human" |
| "Teriparatide" AND "Osteoporosis" AND “CKD” AND "3 point bending" AND "Human" AND "Clinical trial" |
| "Teriparatide" AND "Osteoporosis" AND “CKD” AND "Degree of mineralization" AND "Human" |
| "Teriparatide" AND "Osteoporosis" AND “CKD” AND "Heterogeneity index" AND "Human" |
| "Teriparatide" AND "Osteoporosis" AND “CKD” AND "Microdamage accumulation" AND "Human" |
| "Teriparatide" AND "Osteoporosis" AND “CKD” AND "Mineral matrix ratio" AND "Human" |
| "Teriparatide" AND "Osteoporosis" AND “CKD” AND "Nanoindentation" AND "Human" AND “Clinical trial” |
| "Teriparatide" AND "Osteoporosis" AND “CKD” AND "Pentosidine" AND "Human" |
| "Teriparatide" AND "Osteoporosis" AND “CKD” AND "Bone loss" AND "Human" AND “Clinical trial”- |
| "Teriparatide" AND "Osteoporosis" AND “CKD” AND "carbonate to amide ratio" AND "Human" |
| "Teriparatide" AND "Osteoporosis" AND “CKD” AND "Crystallinity" AND "Human" AND “Clinical trial” |
| "Teriparatide" AND "osteoporosis" AND “CKD” AND "Enzymatic to non-enzymatic cross-linking ratio" AND "Human" |
| "Teriparatide" AND "Osteoporosis" AND “CKD”AND "carbonate to phosphate ratio" AND "Human" |
| "Teriparatide" AND "Osteoporosis" AND “DIABETES” AND "3 point bending" AND "Human" AND "Clinical trial" |
| "Teriparatide" AND "Osteoporosis" AND “DIABETES” AND "Degree of mineralization" AND "Human" |
| "Teriparatide" AND "Osteoporosis" AND “DIABETES” AND "Heterogeneity index" AND "Human" |
| "Teriparatide" AND "Osteoporosis" AND “DIABETES” AND "Microdamage accumulation" AND "Human" |
| "Teriparatide" AND "Osteoporosis" AND “DIABETES” AND "Mineral matrix ratio" AND "Human" |
| "Teriparatide" AND "Osteoporosis" AND “DIABETES” AND "Nanoindentation" AND "Human" AND “Clinical trial” |
| "Teriparatide" AND "Osteoporosis" AND “DIABETES” AND "Pentosidine" AND "Human" |
| "Teriparatide" AND "Osteoporosis" AND “DIABETES” AND "Bone loss" AND "Human" AND “Clinical trial” |
| "Teriparatide" AND "Osteoporosis" AND “DIABETES” AND "carbonate to amide ratio" AND "Human" |
| "Teriparatide" AND "Osteoporosis" AND “DIABETES” AND "Crystallinity" AND "Human" AND “Clinical trial” |
| "Teriparatide" AND "osteoporosis" AND “DIABETES” AND "Enzymatic to non-enzymatic cross-linking ratio" AND "Human" |
| "Teriparatide" AND "Osteoporosis" AND “DIABETES”AND "carbonate to phosphate ratio" AND "Human" |
| "Teriparatide" AND "Osteoporosis" AND “GIO” AND "3 point bending" AND "Human" AND "Clinical trial" |
| "Teriparatide" AND "Osteoporosis" AND “GIO” AND "Degree of mineralization" AND "Human" |
| "Teriparatide" AND "Osteoporosis" AND “GIO” AND "Heterogeneity index" AND "Human" |
| "Teriparatide" AND "Osteoporosis" AND “GIO” AND "Microdamage accumulation" AND "Human" |
| "Teriparatide" AND "Osteoporosis" AND “GIO” AND "Mineral matrix ratio" AND "Human" |
| "Teriparatide" AND "Osteoporosis" AND “GIO” AND "Nanoindentation" AND "Human" AND “Clinical trial” |
| "Teriparatide" AND "Osteoporosis" AND “GIO” AND "Pentosidine" AND "Human" |
| "Teriparatide" AND "Osteoporosis" AND “GIO” AND "Bone loss" AND "Human" AND “Clinical trial” |
| "Teriparatide" AND "Osteoporosis" AND “GIO” AND "carbonate to amide ratio" AND "Human" |
| "Teriparatide" AND "Osteoporosis" AND “GIO” AND "Crystallinity" AND "Human" AND “Clinical trial” |
| "Teriparatide" AND "osteoporosis" AND “GIO” AND "Enzymatic to non-enzymatic cross-linking ratio" AND "Human" |
| "Teriparatide" AND "Osteoporosis" AND “GIO”AND "carbonate to phosphate ratio" AND "Human" |
| "Teriparatide" AND "Osteoporosis" AND “Hypertension” AND "3 point bending" AND "Human" AND "Clinical trial" |
| "Teriparatide" AND "Osteoporosis" AND “Hypertension” AND "Degree of mineralization" AND "Human" |
| "Teriparatide" AND "Osteoporosis" AND “Hypertension” AND "Heterogeneity index" AND "Human" |
| "Teriparatide" AND "Osteoporosis" AND “Hypertension” AND "Microdamage accumulation" AND "Human" |
| "Teriparatide" AND "Osteoporosis" AND “Hypertension” AND "Mineral matrix ratio" AND "Human" |
| "Teriparatide" AND "Osteoporosis" AND “Hypertension” AND "Nanoindentation" AND "Human" AND “Clinical trial” |
| "Teriparatide" AND "Osteoporosis" AND “Hypertension” AND "Pentosidine" AND "Human" |
| "Teriparatide" AND "Osteoporosis" AND “Hypertension” AND "Bone loss" AND "Human" AND “Clinical trial” |
| "Teriparatide" AND "Osteoporosis" AND “Hypertension” AND "carbonate to amide ratio" AND "Human" |
| "Teriparatide" AND "Osteoporosis" AND “Hypertension” AND "Crystallinity" AND "Human" AND “Clinical trial” |
| "Teriparatide" AND "osteoporosis" AND “Hypertension” AND "Enzymatic to non-enzymatic cross-linking ratio" AND "Human" |
| "Teriparatide" AND "Osteoporosis" AND “Hypertension” AND "carbonate to phosphate ratio" AND "Human" |
| "Teriparatide" AND "Osteoporosis" AND “IBD” AND "3 point bending" AND "Human" AND "Clinical trial" |
| "Teriparatide" AND "Osteoporosis" AND “IBD” AND "3 point bending" AND "Human" AND "Clinical trial" |
| "Teriparatide" AND "Osteoporosis" AND “IBD” AND "Degree of mineralization" AND "Human" |
| "Teriparatide" AND "Osteoporosis" AND “IBD” AND "Heterogeneity index" AND "Human" |
| "Teriparatide" AND "Osteoporosis" AND “IBD” AND "Microdamage accumulation" AND "Human" |
| "Teriparatide" AND "Osteoporosis" AND “IBD” AND "Mineral matrix ratio" AND "Human" |
| "Teriparatide" AND "Osteoporosis" AND “IBD” AND "Pentosidine" AND "Human" |
| "Teriparatide" AND "Osteoporosis" AND “IBD” AND "Bone loss" AND "Human" AND “Clinical trial” |
| "Teriparatide" AND "Osteoporosis" AND “IBD” AND "carbonate to amide ratio" AND "Human" |
| "Teriparatide" AND "Osteoporosis" AND “IBD” AND "Crystallinity" AND "Human" AND “Clinical trial” |
| "Teriparatide" AND "osteoporosis" AND “IBD” AND "Enzymatic to non-enzymatic cross-linking ratio" AND "Human" |
| "Teriparatide" AND "Osteoporosis" AND “IBD”AND "carbonate to phosphate ratio" AND "Human" |
| "Teriparatide" AND "Osteoporosis" AND “Arthritis” AND "Advanced glycation end products (AGEs)" AND "Human" |
| "Teriparatide" AND "Osteoporosis" AND “Arthritis” AND "Advanced glycation end products (AGEs)" AND "Human" |
| "Teriparatide" AND "Osteoporosis" AND “CKD” AND "Advanced glycation end products (AGEs)" AND "Human" |
| "Teriparatide" AND "Osteoporosis" AND “DIABETES” AND "Advanced glycation end products (AGEs)" AND "Human" |
| "Teriparatide" AND "Osteoporosis" AND “GIO” AND "Advanced glycation end products (AGEs)" AND "Human" |
| "Teriparatide" AND "Osteoporosis" AND “Hypertension” AND "Advanced glycation end products (AGEs)" AND "Human" |
| "Teriparatide" AND "Osteoporosis" AND “IBD” AND "Advanced glycation end products (AGEs)" AND "Human" |

**Keywords in PubMed**

| "Bisphosphonate" AND "Osteoporosis" AND "carbonate to phosphate ratio" AND "Human" |
| --- |
| "Bisphosphonate" AND "Osteoporosis" AND "carbonate to amide ratio" AND "Human" |
| "Bisphosphonate" AND "Osteoporosis" AND "Mineral matrix ratio" AND "Human" |
| "Bisphosphonate" AND "Osteoporosis" AND "Collagen maturity" AND "Human" |
| "Bisphosphonate" AND "Osteoporosis" AND "Microdamage accumulation" AND "Human" |
| "Bisphosphonate" AND "Osteoporosis" AND "Heterogeneity index" AND "Human" |
| "Bisphosphonate" AND "Osteoporosis" AND "Pentosidine" AND "Human" |
| "Bisphosphonate" AND "Osteoporosis" AND "Degree of mineralization" AND "Human" |
| "Bisphosphonate" AND "Osteoporosis" AND "Homocysteine" AND "Human" AND "Clinical trial" |
| "Bisphosphonate" AND "Osteoporosis" AND "FEA" AND "Human" AND "Clinical trial" |
| "Bisphosphonate" AND "Osteoporosis" AND "3 point bending" AND "Human" AND "Clinical trial" |
| "Bisphosphonate" AND "Osteoporosis" AND "Nanoindentation" AND "Human" AND “Clinical trial” |
| "Bisphosphonate" AND "Osteoporosis" AND "Crystallinity" AND "Human" AND “Clinical trial” |
| "Bisphosphonate"AND "Osteoporosis" AND "Advanced glycation end products (AGEs)" AND "Human" |
| "Bisphosphonate" AND "Osteoporosis" AND "Bone loss" AND "Human" AND “Clinical trial” |
| "Bisphosphonate" AND "osteoporosis" AND "Enzymatic to non-enzymatic cross-linking ratio" AND "Human" |
| "Denosumab" AND "Osteoporosis" AND "carbonate to phosphate ratio" AND "Human" |
| "Denosumab" AND "Osteoporosis" AND "carbonate to amide ratio" AND "Human" |
| "Denosumab" AND "Osteoporosis" AND" Mineral matrix ratio" AND "Human" |
| "Denosumab" AND "Osteoporosis" AND "Collagen maturity" AND "Human" |
| "Denosumab" AND "Osteoporosis" AND "Microdamage accumulation" AND "Human" |
| AND "Osteoporosis" AND "Heterogeneity index" AND "Human" |
| "Denosumab" AND "Osteoporosis" AND "Pentosidine" AND "Human" |
| "Denosumab" AND "Osteoporosis" AND "Degree of mineralization" AND "Human" |
| "Denosumab" AND "Osteoporosis" AND "Homocysteine" AND "Human" AND "Clinical trial" |
| "Denosumab" AND "Osteoporosis" AND "FEA" AND "Human" AND "Clinical trial" |
| "Denosumab'' AND "Osteoporosis" AND "3 point bending" AND "Human" AND "Clinical trial" |
| "Denosumab'' AND "Osteoporosis" AND "Nanoindentation" AND "Human" AND “Clinical trial” |
| "Denosumab'' AND "Osteoporosis" AND "Crystallinity" AND "Human" AND “Clinical trial” |
| "Denosumab'' AND "Osteoporosis" AND "Advanced glycation end products (AGEs)" AND "Human" |
| "Denosumab'' AND "Osteoporosis" AND "Bone loss" |
| "Denosumab" AND "osteoporosis" AND "Enzymatic to non-enzymatic cross-linking ratio" AND "Human" |
| "Teriparatide" AND "Osteoporosis" AND "carbonate to phosphate ratio" AND "Human" |
| "Teriparatide" AND "Osteoporosis" AND "carbonate to amide ratio" |
| "Teriparatide" AND "Osteoporosis" AND "Mineral matrix ratio" AND "Human" |
| "Teriparatide" AND "Osteoporosis" AND "Collagen maturity" AND "Human" |
| "Teriparatide" AND "Osteoporosis" AND "Microdamage accumulation" AND "Human" |
| "Teriparatide" AND "Osteoporosis" AND "Heterogeneity index" AND "Human" |
| "Teriparatide" AND "Osteoporosis" AND "Pentosidine" AND "Human" |
| "Teriparatide" AND "Osteoporosis" AND "Degree of mineralization" AND "Human" |
| "Teriparatide" AND "Osteoporosis" AND "Homocysteine" AND "Human" |
| "Teriparatide" AND "Osteoporosis" AND "FEA" AND "Human" |
| "Teriparatide" AND "Osteoporosis" AND "3 point bending" AND "Human" |
| "Teriparatide" AND "Osteoporosis" AND "Nanoindentation" AND "Human" |
| "Teriparatide" AND "Osteoporosis" AND "Crystallinity" AND "Human" |
| "Teriparatide" AND "Osteoporosis" AND "Advanced glycation end products (AGEs)" AND "Human" |
| "Teriparatide" AND "Osteoporosis" AND "Bone loss" AND "Human" |
| "Teriparatide" AND "osteoporosis" AND "Enzymatic to non-enzymatic cross-linking ratio" AND "Human" |
| "Abaloparatide" AND "Osteoporosis" AND "carbonate to phosphate ratio" AND "Human" |
| "Abaloparatide" AND "Osteoporosis" AND "carbonate to amide ratio" |
| "Abaloparatide" AND "Osteoporosis" AND "Mineral matrix ratio" AND "Human" |
| "Abaloparatide" AND "Osteoporosis" AND "Collagen maturity" AND "Human" |
| "Abaloparatide" AND "Osteoporosis" AND "Microdamage accumulation" AND "Human" |
| "Abaloparatide" AND "Osteoporosis" AND "Heterogeneity index" AND "Human" |
| "Abaloparatide" AND "Osteoporosis" AND "Pentosidine" AND "Human" |
| "Abaloparatide" AND "Osteoporosis" AND "Degree of mineralization" AND "Human" |
| "Abaloparatide" AND "Osteoporosis" AND "Homocysteine" AND "Human" |
| "Abaloparatide" AND "Osteoporosis" AND "FEA" AND "Human" |
| "Abaloparatide" AND "Osteoporosis" AND "3 point bending" AND "Human" |
| "Abaloparatide" AND "Osteoporosis" AND "Nanoindentation" AND "Human" |
| "Abaloparatide" AND "Osteoporosis" AND "Crystallinity" AND "Human" |
| "Abaloparatide" AND "Osteoporosis" AND "Advanced glycation end products (AGEs)" AND "Human" |
| "Abaloparatide" AND "Osteoporosis" AND "Bone loss" AND "Human" |
| "Abaloparatide" AND "osteoporosis" AND "Enzymatic to non-enzymatic cross-linking ratio" AND "Human" |
| "Raloxifene" AND "Osteoporosis" AND "carbonate to phosphate ratio" AND "Human" |
| "Raloxifene" AND "Osteoporosis" AND "carbonate to amide ratio" |
| "Raloxifene" AND "Osteoporosis" AND "Mineral matrix ratio" AND "Human" |
| "Raloxifene" AND "Osteoporosis" AND "Collagen maturity" AND "Human" |
| "Raloxifene" AND "Osteoporosis" AND "Microdamage accumulation" AND "Human" |
| "Raloxifene" AND "Osteoporosis" AND "Heterogeneity index" AND "Human" |
| "Raloxifene" AND "Osteoporosis" AND "Pentosidine" AND "Human" |
| "Raloxifene" AND "Osteoporosis" AND "Degree of mineralization" AND "Human" |
| "Raloxifene" AND "Osteoporosis" AND "Homocysteine" AND "Human" |
| "Raloxifene" AND "Osteoporosis" AND "FEA" AND "Human" |
| "Raloxifene" AND "Osteoporosis" AND "3 point bending" AND "Human" |
| "Raloxifene" AND "Osteoporosis" AND "Nanoindentation" AND "Human" |
| "Raloxifene" AND "Osteoporosis" AND "Crystallinity" AND "Human" |
| "Raloxifene" AND "Osteoporosis" AND "Advanced glycation end products (AGEs)" AND "Human" |
| "Raloxifene" AND "Osteoporosis" AND "Bone loss" AND "Human" |
| "Raloxifene" AND "osteoporosis" AND "Enzymatic to non-enzymatic cross-linking ratio" AND "Human" |
| "Romosozumab" AND "Osteoporosis" AND "carbonate to phosphate ratio" AND "Human" |
| "Romosozumab" AND "Osteoporosis" AND "carbonate to amide ratio" |
| "Romosozumab" AND "Osteoporosis" AND "Mineral matrix ratio" AND "Human" |
| "Romosozumab" AND "Osteoporosis" AND "Collagen maturity" AND "Human" |
| "Romosozumab" AND "Osteoporosis" AND "Microdamage accumulation" AND "Human" |
| "Romosozumab" AND "Osteoporosis" AND "Heterogeneity index" AND "Human" |
| "Romosozumab" AND "Osteoporosis" AND "Pentosidine" AND "Human" |
| "Romosozumab" AND "Osteoporosis" AND "Degree of mineralization" AND "Human" |
| "Romosozumab" AND "Osteoporosis" AND "Homocysteine" AND "Human" |
| "Romosozumab" AND "Osteoporosis" AND "FEA" AND "Human" |
| "Romosozumab" AND "Osteoporosis" AND "3 point bending" AND "Human" |
| "Romosozumab" AND "Osteoporosis" AND "Nanoindentation" AND "Human" |
| "Romosozumab" AND "Osteoporosis" AND "Crystallinity" AND "Human" |
| "Romosozumab" AND "Osteoporosis" AND "Advanced glycation end products (AGEs)" AND "Human" |
| "Romosozumab" AND "Osteoporosis" AND "Bone loss" AND "Human" |
| "Romosozumab" AND "osteoporosis" AND "Enzymatic to non-enzymatic cross-linking ratio" AND "Human" |
| "Strontium ranelate" AND "Osteoporosis" AND "carbonate to phosphate ratio" AND "Human" |
| "Strontium ranelate" AND "Osteoporosis" AND "carbonate to amide ratio" |
| "Strontium ranelate" AND "Osteoporosis" AND "Mineral matrix ratio" AND "Human" |
| "Strontium ranelate" AND "Osteoporosis" AND "Collagen maturity" AND "Human" |
| "Strontium ranelate" AND "Osteoporosis" AND "Microdamage accumulation" AND "Human" |
| "Strontium ranelate" AND "Osteoporosis" AND "Heterogeneity index" AND "Human" |
| "Strontium ranelate" AND "Osteoporosis" AND "Pentosidine" AND "Human" |
| "Strontium ranelate" AND "Osteoporosis" AND "Degree of mineralization" AND "Human" |
| "Strontium ranelate" AND "Osteoporosis" AND "Homocysteine" AND "Human" |
| "Strontium ranelate" AND "Osteoporosis" AND "FEA" AND "Human" |
| "Strontium ranelate" AND "Osteoporosis" AND "3 point bending" AND "Human" |
| "Strontium ranelate" AND "Osteoporosis" AND "Nanoindentation" AND "Human" |
| "Strontium ranelate" AND "Osteoporosis" AND "Crystallinity" AND "Human" |
| "Strontium ranelate" AND "Osteoporosis" AND "Advanced glycation end products (AGEs)" AND "Human" |
| "Strontium ranelate" AND "Osteoporosis" AND "Bone loss" AND "Human" |
| "Strotium ranelate" AND "osteoporosis" AND "Enzymatic to non-enzymatic cross-linking ratio" AND "Human" |

| "Bisphosphonate" AND "Osteoporosis" AND “Arthritis” AND "carbonate to phosphate ratio" AND "Human" | |
| --- | --- |
|  | "Bisphosphonate" AND "Osteoporosis" AND “Arthritis” AND "carbonate to amide ratio" AND "Human" |
|  | "Bisphosphonate" AND "Osteoporosis" AND “Arthritis” AND "Mineral matrix ratio" AND "Human" |
|  | "Bisphosphonate" AND “Arthritis” AND "Osteoporosis" AND "Collagen maturity" AND "Human" |
|  | "Bisphosphonate" AND "Osteoporosis" AND “Arthritis” AND "Microdamage accumulation" AND "Human" |
|  | "Bisphosphonate" AND "Osteoporosis" AND “Arthritis” AND "Heterogeneity index" AND "Human" |
|  | "Bisphosphonate" AND "Osteoporosis" AND “Arthritis” AND "Pentosidine" AND "Human" |
|  | "Bisphosphonate" AND "Osteoporosis" AND “Arthritis” AND "Degree of mineralization" AND "Human" |
|  | "Bisphosphonate" AND "Osteoporosis" AND “Arthritis” AND "Homocysteine" AND "Human" AND "Clinical trial" |
|  | "Bisphosphonate" AND "Osteoporosis" AND “Arthritis” AND "FEA" AND "Human" AND "Clinical trial" |
|  | "Bisphosphonate" AND "Osteoporosis" AND “Arthritis” AND "3 point bending" AND "Human" AND "Clinical trial" |
|  | "Bisphosphonate" AND "Osteoporosis" AND “Arthritis” AND "Nanoindentation" AND "Human" AND “Clinical trial” |
|  | "Bisphosphonate" AND "Osteoporosis" AND “Arthritis” AND "Crystallinity" AND "Human" AND “Clinical trial” |
|  | "Bisphosphonate" AND "Osteoporosis" AND “Arthritis” AND "Advanced glycation end products (AGEs)" AND "Human" |
|  | "Bisphosphonate" AND "Osteoporosis" AND “Arthritis” AND "Bone loss" AND "Human" AND “Clinical trial” |
|  | "Bisphosphonate" AND "osteoporosis" AND “Arthritis” AND "Enzymatic to non-enzymatic cross-linking ratio" AND "Human" |
|  | "Denosumab" AND "Osteoporosis" AND “Arthritis” AND "carbonate to phosphate ratio" AND "Human" |
|  | "Denosumab" AND "Osteoporosis" AND “Arthritis” AND "carbonate to amide ratio" AND "Human" |
|  | "Denosumab" AND "Osteoporosis" AND “Arthritis” AND "Mineral matrix ratio" AND "Human" |
|  | "Denosumab" AND “Arthritis” AND "Osteoporosis" AND "Collagen maturity" AND "Human" |
|  | "Denosumab" AND "Osteoporosis" AND “Arthritis” AND "Microdamage accumulation" AND "Human" |
|  | "Denosumab" AND "Osteoporosis" AND “Arthritis” AND "Heterogeneity index" AND "Human" |
|  | "Denosumab" AND "Osteoporosis" AND “Arthritis” AND "Pentosidine" AND "Human" |
|  | "Denosumab" AND "Osteoporosis" AND “Arthritis” AND "Degree of mineralization" AND "Human" |
|  | "Denosumab" AND "Osteoporosis" AND “Arthritis” AND "Homocysteine" AND "Human" AND "Clinical trial" |
|  | "Denosumab" AND "Osteoporosis" AND “Arthritis” AND "FEA" AND "Human" AND "Clinical trial" |
|  | "Denosumab" AND "Osteoporosis" AND “Arthritis” AND "3 point bending" AND "Human" AND "Clinical trial" |
|  | "Denosumab" AND "Osteoporosis" AND “Arthritis” AND "Nanoindentation" AND "Human" AND “Clinical trial” |
|  | "Denosumab" AND "Osteoporosis" AND “Arthritis” AND "Crystallinity" AND "Human" AND “Clinical trial” |
|  | "Denosumab" AND "Osteoporosis" AND “Arthritis” AND "Advanced glycation end products (AGEs)" AND "Human" |
|  | "Denosumab" AND "Osteoporosis" AND “Arthritis” AND "Bone loss" AND "Human" AND “Clinical trial” |
|  | "Denosumab" AND "osteoporosis" AND “Arthritis” AND "Enzymatic to non-enzymatic cross-linking ratio" AND "Human" |
|  | "Teriparatide" AND "Osteoporosis" AND “Arthritis” AND "carbonate to phosphate ratio" AND "Human" |
|  | "Teriparatide" AND "Osteoporosis" AND “Arthritis” AND "carbonate to amide ratio" AND "Human" |
|  | "Teriparatide" AND "Osteoporosis" AND “Arthritis” AND "Mineral matrix ratio" AND "Human" |
|  | "Teriparatide" AND “Arthritis” AND "Osteoporosis" AND "Collagen maturity" AND "Human" |
|  | "Teriparatide" AND "Osteoporosis" AND “Arthritis” AND "Microdamage accumulation" AND "Human" |
|  | "Teriparatide" AND "Osteoporosis" AND “Arthritis” AND "Heterogeneity index" AND "Human" |
|  | "Teriparatide" AND "Osteoporosis" AND “Arthritis” AND "Pentosidine" AND "Human" |
|  | "Teriparatide" AND "Osteoporosis" AND “Arthritis” AND "Degree of mineralization" AND "Human" |
|  | "Teriparatide" AND "Osteoporosis" AND “Arthritis” AND "Homocysteine" AND "Human" AND "Clinical trial" |
|  | "Teriparatide" AND "Osteoporosis" AND “Arthritis” AND "FEA" AND "Human" AND "Clinical trial" |
|  | "Teriparatide" AND "Osteoporosis" AND “Arthritis” AND "3 point bending" AND "Human" AND "Clinical trial" |
|  | "Teriparatide" AND "Osteoporosis" AND “Arthritis” AND "Nanoindentation" AND "Human" AND “Clinical trial” |
|  | "Teriparatide" AND "Osteoporosis" AND “Arthritis” AND "Crystallinity" AND "Human" AND “Clinical trial” |
|  | "Teriparatide" AND "Osteoporosis" AND “Arthritis” AND "Advanced glycation end products (AGEs)" AND "Human" |
|  | "Teriparatide" AND "Osteoporosis" AND “Arthritis” AND "Bone loss" AND "Human" AND “Clinical trial” |
|  | "Teriparatide" AND "osteoporosis" AND “Arthritis” AND "Enzymatic to non-enzymatic cross-linking ratio" AND "Human" |
|  | "Teriparatide" AND "Osteoporosis" AND “Arthritis” AND "carbonate to phosphate ratio" AND "Human" |
|  | "Teriparatide" AND "Osteoporosis" AND “Arthritis” AND "carbonate to amide ratio" AND "Human" |
|  | "Teriparatide" AND "Osteoporosis" AND “Arthritis” AND "Mineral matrix ratio" AND "Human" |
|  | "Teriparatide" AND “Arthritis” AND "Osteoporosis" AND "Collagen maturity" AND "Human" |
|  | "Teriparatide" AND "Osteoporosis" AND “Arthritis” AND "Microdamage accumulation" AND "Human" |
|  | "Teriparatide" AND "Osteoporosis" AND “Arthritis” AND "Heterogeneity index" AND "Human" |
|  | "Teriparatide" AND "Osteoporosis" AND “Arthritis” AND "Pentosidine" AND "Human" |
|  | "Teriparatide" AND "Osteoporosis" AND “Arthritis” AND "Degree of mineralization" AND "Human" |
|  | "Teriparatide" AND "Osteoporosis" AND “Arthritis” AND "Homocysteine" AND "Human" AND "Clinical trial" |
|  | "Teriparatide" AND "Osteoporosis" AND “Arthritis” AND "FEA" AND "Human" AND "Clinical trial" |
|  | "Teriparatide" AND "Osteoporosis" AND “Arthritis” AND "3 point bending" AND "Human" AND "Clinical trial" |
|  | "Teriparatide" AND "Osteoporosis" AND “Arthritis” AND "Nanoindentation" AND "Human" AND “Clinical trial” |
|  | "Teriparatide" AND "Osteoporosis" AND “Arthritis” AND "Crystallinity" AND "Human" AND “Clinical trial” |
|  | "Teriparatide" AND "Osteoporosis" AND “Arthritis” AND "Advanced glycation end products (AGEs)" AND "Human" |
|  | "Teriparatide" AND "Osteoporosis" AND “Arthritis” AND "Bone loss" AND "Human" AND “Clinical trial” |
|  | "Teriparatide" AND "osteoporosis" AND “Arthritis” AND "Enzymatic to non-enzymatic cross-linking ratio" AND "Human" |
|  | "Abaloparatide" AND "Osteoporosis" AND “Arthritis” AND "carbonate to phosphate ratio" AND "Human" |
|  | "Abaloparatide" AND "Osteoporosis" AND “Arthritis” AND "carbonate to amide ratio" AND "Human" |
|  | "Abaloparatide" AND "Osteoporosis" AND “Arthritis” AND "Mineral matrix ratio" AND "Human" |
|  | "Abaloparatide" AND “Arthritis” AND "Osteoporosis" AND "Collagen maturity" AND "Human" |
|  | "Abaloparatide" AND "Osteoporosis" AND “Arthritis” AND "Microdamage accumulation" AND "Human" |
|  | "Abaloparatide" AND "Osteoporosis" AND “Arthritis” AND "Heterogeneity index" AND "Human" |
|  | "Abaloparatide" AND "Osteoporosis" AND “Arthritis” AND "Pentosidine" AND "Human" |
|  | "Abaloparatide" AND "Osteoporosis" AND “Arthritis” AND "Degree of mineralization" AND "Human" |
|  | "Abaloparatide" AND "Osteoporosis" AND “Arthritis” AND "Homocysteine" AND "Human" AND "Clinical trial" |
|  | "Abaloparatide" AND "Osteoporosis" AND “Arthritis” AND "FEA" AND "Human" AND "Clinical trial" |
|  | "Abaloparatide" AND "Osteoporosis" AND “Arthritis” AND "3 point bending" AND "Human" AND "Clinical trial" |
|  | "Abaloparatide" AND "Osteoporosis" AND “Arthritis” AND "Nanoindentation" AND "Human" AND “Clinical trial” |
|  | "Abaloparatide" AND "Osteoporosis" AND “Arthritis” AND "Crystallinity" AND "Human" AND “Clinical trial” |
|  | "Abaloparatide" AND "Osteoporosis" AND “Arthritis” AND "Advanced glycation end products (AGEs)" AND "Human" |
|  | "Abaloparatide" AND "Osteoporosis" AND “Arthritis” AND "Bone loss" AND "Human" AND “Clinical trial” |
|  | "Abaloparatide" AND "osteoporosis" AND “Arthritis” AND "Enzymatic to non-enzymatic cross-linking ratio" AND "Human" |
|  | "Raloxifene" AND "Osteoporosis" AND “Arthritis” AND "carbonate to phosphate ratio" AND "Human" |
|  | "Raloxifene" AND "Osteoporosis" AND “Arthritis” AND "carbonate to amide ratio" AND "Human" |
|  | "Raloxifene" AND "Osteoporosis" AND “Arthritis” AND "Mineral matrix ratio" AND "Human" |
|  | "Raloxifene" AND “Arthritis” AND "Osteoporosis" AND "Collagen maturity" AND "Human" |
|  | "Raloxifene" AND "Osteoporosis" AND “Arthritis” AND "Microdamage accumulation" AND "Human" |
|  | "Raloxifene" AND "Osteoporosis" AND “Arthritis” AND "Heterogeneity index" AND "Human" |
|  | "Raloxifene" AND "Osteoporosis" AND “Arthritis” AND "Pentosidine" AND "Human" |
|  | "Raloxifene" AND "Osteoporosis" AND “Arthritis” AND "Degree of mineralization" AND "Human" |
|  | "Raloxifene" AND "Osteoporosis" AND “Arthritis” AND "Homocysteine" AND "Human" AND "Clinical trial" |
|  | "Raloxifene" AND "Osteoporosis" AND “Arthritis” AND "FEA" AND "Human" AND "Clinical trial" |
|  | "Raloxifene" AND "Osteoporosis" AND “Arthritis” AND "3 point bending" AND "Human" AND "Clinical trial" |
|  | "Raloxifene" AND "Osteoporosis" AND “Arthritis” AND "Nanoindentation" AND "Human" AND “Clinical trial” |
|  | "Raloxifene" AND "Osteoporosis" AND “Arthritis” AND "Crystallinity" AND "Human" AND “Clinical trial” |
|  | "Raloxifene"AND "Osteoporosis" AND “Arthritis” AND "Advanced glycation end products (AGEs)" AND "Human" |
|  | "Raloxifene" AND "Osteoporosis" AND “Arthritis” AND "Bone loss" AND "Human" AND “Clinical trial” |
|  | "Raloxifene" AND "osteoporosis" AND “Arthritis” AND "Enzymatic to non-enzymatic cross-linking ratio" AND "Human" |
|  | "Romosozumab" AND "Osteoporosis" AND “Arthritis” AND "carbonate to phosphate ratio" AND "Human" |
|  | "Romosozumab" AND "Osteoporosis" AND “Arthritis” AND "carbonate to amide ratio" AND "Human" |
|  | "Romosozumab" AND "Osteoporosis" AND “Arthritis” AND "Mineral matrix ratio" AND "Human" |
|  | "Romosozumab" AND “Arthritis” AND "Osteoporosis" AND "Collagen maturity" AND "Human" |
|  | "Romosozumab" AND "Osteoporosis" AND “Arthritis” AND "Microdamage accumulation" AND "Human" |
|  | "Romosozumab" AND "Osteoporosis" AND “Arthritis” AND "Heterogeneity index" AND "Human" |
|  | "Romosozumab" AND "Osteoporosis" AND “Arthritis” AND "Pentosidine" AND "Human" |
|  | "Romosozumab" AND "Osteoporosis" AND “Arthritis” AND "Degree of mineralization" AND "Human" |
|  | "Romosozumab" AND "Osteoporosis" AND “Arthritis” AND "Homocysteine" AND "Human" AND "Clinical trial" |
|  | "Romosozumab" AND "Osteoporosis" AND “Arthritis” AND "FEA" AND "Human" AND "Clinical trial" |
|  | "Romosozumab" AND "Osteoporosis" AND “Arthritis” AND "3 point bending" AND "Human" AND "Clinical trial" |
|  | "Romosozumab" AND "Osteoporosis" AND “Arthritis” AND "Nanoindentation" AND "Human" AND “Clinical trial” |
|  | "Romosozumab" AND "Osteoporosis" AND “Arthritis” AND "Crystallinity" AND "Human" AND “Clinical trial” |
|  | "Romosozumab"AND "Osteoporosis" AND “Arthritis” AND "Advanced glycation end products (AGEs)" AND "Human" |
|  | "Romosozumab" AND "Osteoporosis" AND “Arthritis” AND "Bone loss" AND "Human" AND “Clinical trial” |
|  | "Romosozumab" AND "osteoporosis" AND “Arthritis” AND "Enzymatic to non-enzymatic cross-linking ratio" AND "Human" |
|  | "Strontium ranelate" AND "Osteoporosis" AND “Arthritis” AND "carbonate to phosphate ratio" AND "Human" |
|  | "Strontium ranelate" AND "Osteoporosis" AND “Arthritis” AND "carbonate to amide ratio" AND "Human" |
|  | "Strontium ranelate" AND "Osteoporosis" AND “Arthritis” AND "Mineral matrix ratio" AND "Human" |
|  | "Strontium ranelate" AND “Arthritis” AND "Osteoporosis" AND "Collagen maturity" AND "Human" |
|  | "Strontium ranelate" AND "Osteoporosis" AND “Arthritis” AND "Microdamage accumulation" AND "Human" |
|  | "Strontium ranelate" AND "Osteoporosis" AND “Arthritis” AND "Heterogeneity index" AND "Human" |
|  | "Strontium ranelate" AND "Osteoporosis" AND “Arthritis” AND "Pentosidine" AND "Human" |
|  | "Strontium ranelate" AND "Osteoporosis" AND “Arthritis” AND "Degree of mineralization" AND "Human" |
|  | "Strontium ranelate" AND "Osteoporosis" AND “Arthritis” AND "Homocysteine" AND "Human" AND "Clinical trial" |
|  | "Strontium ranelate" AND "Osteoporosis" AND “Arthritis” AND "FEA" AND "Human" AND "Clinical trial" |
|  | "Strontium ranelate" AND "Osteoporosis" AND “Arthritis” AND "3 point bending" AND "Human" AND "Clinical trial" |
|  | "Strontium ranelate" AND "Osteoporosis" AND “Arthritis” AND "Nanoindentation" AND "Human" AND “Clinical trial” |
|  | "Strontium ranelate" AND "Osteoporosis" AND “Arthritis” AND "Crystallinity" AND "Human" AND “Clinical trial” |
|  | "Strontium ranelate" AND "Osteoporosis" AND “Arthritis” AND "Advanced glycation end products (AGEs)" AND "Human" |
|  | "Strontium ranelate" AND "Osteoporosis" AND “Arthritis” AND "Bone loss" AND "Human" AND “Clinical trial” |
|  | "Strontium ranelate" AND "osteoporosis" AND “Arthritis” AND "Enzymatic to non-enzymatic cross-linking ratio" AND "Human" |
|  | "Bisphosphonate" AND "Osteoporosis" AND “CKD”AND "carbonate to phosphate ratio" AND "Human" |
|  | "Bisphosphonate" AND "Osteoporosis" AND “CKD” AND "carbonate to amide ratio" AND "Human" |
|  | "Bisphosphonate" AND "Osteoporosis" AND “CKD” AND "Mineral matrix ratio" AND "Human" |
|  | "Bisphosphonate" AND “CKD”AND "Osteoporosis" AND "Collagen maturity" AND "Human" |
|  | "Bisphosphonate" AND "Osteoporosis" AND “CKD” AND "Microdamage accumulation" AND "Human" |
|  | "Bisphosphonate" AND "Osteoporosis" AND “CKD” AND "Heterogeneity index" AND "Human" |
|  | "Bisphosphonate" AND "Osteoporosis" AND “CKD” AND "Pentosidine" AND "Human" |
|  | "Bisphosphonate" AND "Osteoporosis" AND “CKD” AND "Degree of mineralization" AND "Human" |
|  | "Bisphosphonate" AND "Osteoporosis" AND “CKD”AND "Homocysteine" AND "Human" AND "Clinical trial" |
|  | "Bisphosphonate" AND "Osteoporosis" AND “CKD”AND "FEA" AND "Human" AND "Clinical trial" |
|  | "Bisphosphonate" AND "Osteoporosis" AND “CKD” AND "3 point bending" AND "Human" AND "Clinical trial" |
|  | "Bisphosphonate" AND "Osteoporosis" AND “CKD” AND "Nanoindentation" AND "Human" AND “Clinical trial” |
|  | "Bisphosphonate" AND "Osteoporosis" AND “CKD” AND "Crystallinity" AND "Human" AND “Clinical trial” |
|  | "Bisphosphonate"AND "Osteoporosis" AND “CKD” AND "Advanced glycation end products (AGEs)" AND "Human" |
|  | "Bisphosphonate" AND "Osteoporosis" AND “CKD” AND "Bone loss" AND "Human" AND “Clinical trial” |
|  | "Bisphosphonate" AND "osteoporosis" AND “CKD” AND "Enzymatic to non-enzymatic cross-linking ratio" AND "Human" |
|  | "Denosumab" AND "Osteoporosis" AND “CKD”AND "carbonate to phosphate ratio" AND "Human" |
|  | "Denosumab" AND "Osteoporosis" AND “CKD” AND "carbonate to amide ratio" AND "Human" |
|  | "Denosumab" AND "Osteoporosis" AND “CKD” AND "Mineral matrix ratio" AND "Human" |
|  | "Denosumab" AND “CKD”AND "Osteoporosis" AND "Collagen maturity" AND "Human" |
|  | "Denosumab" AND "Osteoporosis" AND “CKD” AND "Microdamage accumulation" AND "Human" |
|  | "Denosumab" AND "Osteoporosis" AND “CKD” AND "Heterogeneity index" AND "Human" |
|  | "Denosumab" AND "Osteoporosis" AND “CKD” AND "Pentosidine" AND "Human" |
|  | "Denosumab" AND "Osteoporosis" AND “CKD” AND "Degree of mineralization" AND "Human" |
|  | "Denosumab" AND "Osteoporosis" AND “CKD”AND "Homocysteine" AND "Human" AND "Clinical trial" |
|  | "Denosumab" AND "Osteoporosis" AND “CKD”AND "FEA" AND "Human" AND "Clinical trial" |
|  | "Denosumab" AND "Osteoporosis" AND “CKD” AND "3 point bending" AND "Human" AND "Clinical trial" |
|  | "Denosumab" AND "Osteoporosis" AND “CKD” AND "Nanoindentation" AND "Human" AND “Clinical trial” |
|  | "Denosumab" AND "Osteoporosis" AND “CKD” AND "Crystallinity" AND "Human" AND “Clinical trial” |
|  | "Denosumab" AND "Osteoporosis" AND “CKD” AND "Advanced glycation end products (AGEs)" AND "Human" |
|  | "Denosumab" AND "Osteoporosis" AND “CKD” AND "Bone loss" AND "Human" AND “Clinical trial” |
|  | "Denosumab" AND "osteoporosis" AND “CKD” AND "Enzymatic to non-enzymatic cross-linking ratio" AND "Human" |
|  | "Teriparatide" AND "Osteoporosis" AND “CKD”AND "carbonate to phosphate ratio" AND "Human" |
|  | "Teriparatide" AND "Osteoporosis" AND “CKD” AND "carbonate to amide ratio" AND "Human" |
|  | "Teriparatide" AND "Osteoporosis" AND “CKD” AND "Mineral matrix ratio" AND "Human" |
|  | "Teriparatide" AND “CKD”AND "Osteoporosis" AND "Collagen maturity" AND "Human" |
|  | "Teriparatide" AND "Osteoporosis" AND “CKD” AND "Microdamage accumulation" AND "Human" |
|  | "Teriparatide" AND "Osteoporosis" AND “CKD” AND "Heterogeneity index" AND "Human" |
|  | "Teriparatide" AND "Osteoporosis" AND “CKD” AND "Pentosidine" AND "Human" |
|  | "Teriparatide" AND "Osteoporosis" AND “CKD” AND "Degree of mineralization" AND "Human" |
|  | "Teriparatide" AND "Osteoporosis" AND “CKD”AND "Homocysteine" AND "Human" AND "Clinical trial" |
|  | "Teriparatide" AND "Osteoporosis" AND “CKD”AND "FEA" AND "Human" AND "Clinical trial" |
|  | "Teriparatide" AND "Osteoporosis" AND “CKD” AND "3 point bending" AND "Human" AND "Clinical trial" |
|  | "Teriparatide" AND "Osteoporosis" AND “CKD” AND "Nanoindentation" AND "Human" AND “Clinical trial” |
|  | "Teriparatide" AND "Osteoporosis" AND “CKD” AND "Crystallinity" AND "Human" AND “Clinical trial” |
|  | "Teriparatide" AND "Osteoporosis" AND “CKD” AND "Advanced glycation end products (AGEs)" AND "Human" |
|  | "Teriparatide" AND "Osteoporosis" AND “CKD” AND "Bone loss" AND "Human" AND “Clinical trial” |
|  | "Teriparatide" AND "osteoporosis" AND “CKD” AND "Enzymatic to non-enzymatic cross-linking ratio" AND "Human" |
|  | "Abaloparatide" AND "Osteoporosis" AND “CKD”AND "carbonate to phosphate ratio" AND "Human" |
|  | "Abaloparatide" AND "Osteoporosis" AND “CKD” AND "carbonate to amide ratio" AND "Human" |
|  | "Abaloparatide" AND "Osteoporosis" AND “CKD” AND "Mineral matrix ratio" AND "Human" |
|  | "Abaloparatide" AND “CKD”AND "Osteoporosis" AND "Collagen maturity" AND "Human" |
|  | "Abaloparatide" AND "Osteoporosis" AND “CKD” AND "Microdamage accumulation" AND "Human" |
|  | "Abaloparatide" AND "Osteoporosis" AND “CKD” AND "Heterogeneity index" AND "Human" |
|  | "Abaloparatide" AND "Osteoporosis" AND “CKD” AND "Pentosidine" AND "Human" |
|  | "Abaloparatide" AND "Osteoporosis" AND “CKD” AND "Degree of mineralization" AND "Human" |
|  | "Abaloparatide" AND "Osteoporosis" AND “CKD”AND "Homocysteine" AND "Human" AND "Clinical trial" |
|  | "Abaloparatide" AND "Osteoporosis" AND “CKD”AND "FEA" AND "Human" AND "Clinical trial" |
|  | "Abaloparatide" AND "Osteoporosis" AND “CKD” AND "3 point bending" AND "Human" AND "Clinical trial" |
|  | "Abaloparatide" AND "Osteoporosis" AND “CKD” AND "Nanoindentation" AND "Human" AND “Clinical trial” |
|  | "Abaloparatide" AND "Osteoporosis" AND “CKD” AND "Crystallinity" AND "Human" AND “Clinical trial” |
|  | "Abaloparatide" AND "Osteoporosis" AND “CKD” AND "Advanced glycation end products (AGEs)" AND "Human" |
|  | "Abaloparatide" AND "Osteoporosis" AND “CKD” AND "Bone loss" AND "Human" AND “Clinical trial” |
|  | "Abaloparatide" AND "osteoporosis" AND “CKD” AND "Enzymatic to non-enzymatic cross-linking ratio" AND "Human" |
|  | "Raloxifene" AND "Osteoporosis" AND “CKD”AND "carbonate to phosphate ratio" AND "Human" |
|  | "Raloxifene" AND "Osteoporosis" AND “CKD” AND "carbonate to amide ratio" AND "Human" |
|  | "Raloxifene" AND "Osteoporosis" AND “CKD” AND "Mineral matrix ratio" AND "Human" |
|  | "Raloxifene" AND “CKD”AND "Osteoporosis" AND "Collagen maturity" AND "Human" |
|  | "Raloxifene" AND "Osteoporosis" AND “CKD” AND "Microdamage accumulation" AND "Human" |
|  | "Raloxifene" AND "Osteoporosis" AND “CKD” AND "Heterogeneity index" AND "Human" |
|  | "Raloxifene" AND "Osteoporosis" AND “CKD” AND "Pentosidine" AND "Human" |
|  | "Raloxifene" AND "Osteoporosis" AND “CKD” AND "Degree of mineralization" AND "Human" |
|  | "Raloxifene" AND "Osteoporosis" AND “CKD”AND "Homocysteine" AND "Human" AND "Clinical trial" |
|  | "Raloxifene" AND "Osteoporosis" AND “CKD”AND "FEA" AND "Human" AND "Clinical trial" |
|  | "Raloxifene" AND "Osteoporosis" AND “CKD” AND "3 point bending" AND "Human" AND "Clinical trial" |
|  | "Raloxifene" AND "Osteoporosis" AND “CKD” AND "Nanoindentation" AND "Human" AND “Clinical trial” |
|  | "Raloxifene" AND "Osteoporosis" AND “CKD” AND "Crystallinity" AND "Human" AND “Clinical trial” |
|  | "Raloxifene"AND "Osteoporosis" AND “CKD” AND "Advanced glycation end products (AGEs)" AND "Human" |
|  | "Raloxifene" AND "Osteoporosis" AND “CKD” AND "Bone loss" AND "Human" AND “Clinical trial” |
|  | "Raloxifene" AND "osteoporosis" AND “CKD” AND "Enzymatic to non-enzymatic cross-linking ratio" AND "Human" |
|  | "Romosozumab" AND "Osteoporosis" AND “CKD”AND "carbonate to phosphate ratio" AND "Human" |
|  | "Romosozumab" AND "Osteoporosis" AND “CKD” AND "carbonate to amide ratio" AND "Human" |
|  | "Romosozumab" AND "Osteoporosis" AND “CKD” AND "Mineral matrix ratio" AND "Human" |
|  | "Romosozumab" AND “CKD”AND "Osteoporosis" AND "Collagen maturity" AND "Human" |
|  | "Romosozumab" AND "Osteoporosis" AND “CKD” AND "Microdamage accumulation" AND "Human" |
|  | "Romosozumab" AND "Osteoporosis" AND “CKD” AND "Heterogeneity index" AND "Human" |
|  | "Romosozumab" AND "Osteoporosis" AND “CKD” AND "Pentosidine" AND "Human" |
|  | "Romosozumab" AND "Osteoporosis" AND “CKD” AND "Degree of mineralization" AND "Human" |
|  | "Romosozumab" AND "Osteoporosis" AND “CKD”AND "Homocysteine" AND "Human" AND "Clinical trial" |
|  | "Romosozumab" AND "Osteoporosis" AND “CKD”AND "FEA" AND "Human" AND "Clinical trial" |
|  | "Romosozumab" AND "Osteoporosis" AND “CKD” AND "3 point bending" AND "Human" AND "Clinical trial" |
|  | "Romosozumab" AND "Osteoporosis" AND “CKD” AND "Nanoindentation" AND "Human" AND “Clinical trial” |
|  | "Romosozumab" AND "Osteoporosis" AND “CKD” AND "Crystallinity" AND "Human" AND “Clinical trial” |
|  | "Romosozumab"AND "Osteoporosis" AND “CKD” AND "Advanced glycation end products (AGEs)" AND "Human" |
|  | "Romosozumab" AND "Osteoporosis" AND “CKD” AND "Bone loss" AND "Human" AND “Clinical trial” |
|  | "Romosozumab" AND "osteoporosis" AND “CKD” AND "Enzymatic to non-enzymatic cross-linking ratio" AND "Human" |
|  | "Strontium ranelate" AND "Osteoporosis" AND “CKD”AND "carbonate to phosphate ratio" AND "Human" |
|  | "Strontium ranelate" AND "Osteoporosis" AND “CKD” AND "carbonate to amide ratio" AND "Human" |
|  | "Strontium ranelate" AND "Osteoporosis" AND “CKD” AND "Mineral matrix ratio" AND "Human" |
|  | "Strontium ranelate" AND “CKD”AND "Osteoporosis" AND "Collagen maturity" AND "Human" |
|  | "Strontium ranelate" AND "Osteoporosis" AND “CKD” AND "Microdamage accumulation" AND "Human" |
|  | "Strontium ranelate" AND "Osteoporosis" AND “CKD” AND "Heterogeneity index" AND "Human" |
|  | "Strontium ranelate" AND "Osteoporosis" AND “CKD” AND "Pentosidine" AND "Human" |
|  | "Strontium ranelate" AND "Osteoporosis" AND “CKD” AND "Degree of mineralization" AND "Human" |
|  | "Strontium ranelate" AND "Osteoporosis" AND “CKD”AND "Homocysteine" AND "Human" AND "Clinical trial" |
|  | "Strontium ranelate" AND "Osteoporosis" AND “CKD”AND "FEA" AND "Human" AND "Clinical trial" |
|  | "Strontium ranelate" AND "Osteoporosis" AND “CKD” AND "3 point bending" AND "Human" AND "Clinical trial" |
|  | "Strontium ranelate" AND "Osteoporosis" AND “CKD” AND "Nanoindentation" AND "Human" AND “Clinical trial” |
|  | "Strontium ranelate" AND "Osteoporosis" AND “CKD” AND "Crystallinity" AND "Human" AND “Clinical trial” |
|  | "Strontium ranelate" AND "Osteoporosis" AND “CKD” AND "Advanced glycation end products (AGEs)" AND "Human" |
|  | "Strontium ranelate" AND "Osteoporosis" AND “CKD” AND "Bone loss" AND "Human" AND “Clinical trial” |
|  | "Strontium ranelate" AND "osteoporosis" AND “CKD” AND "Enzymatic to non-enzymatic cross-linking ratio" AND "Human" |
|  | "Bisphosphonate" AND "Osteoporosis" AND “GIO”AND "carbonate to phosphate ratio" AND "Human" |
|  | "Bisphosphonate" AND "Osteoporosis" AND “GIO” AND "carbonate to amide ratio" AND "Human" |
|  | "Bisphosphonate" AND "Osteoporosis" AND “GIO” AND "Mineral matrix ratio" AND "Human" |
|  | "Bisphosphonate" AND “GIO”AND "Osteoporosis" AND "Collagen maturity" AND "Human" |
|  | "Bisphosphonate" AND "Osteoporosis" AND “GIO” AND "Microdamage accumulation" AND "Human" |
|  | "Bisphosphonate" AND "Osteoporosis" AND “GIO” AND "Heterogeneity index" AND "Human" |
|  | "Bisphosphonate" AND "Osteoporosis" AND “GIO” AND "Pentosidine" AND "Human" |
|  | "Bisphosphonate" AND "Osteoporosis" AND “GIO” AND "Degree of mineralization" AND "Human" |
|  | "Bisphosphonate" AND "Osteoporosis" AND “GIO”AND "Homocysteine" AND "Human" AND "Clinical trial" |
|  | "Bisphosphonate" AND "Osteoporosis" AND “GIO”AND "FEA" AND "Human" AND "Clinical trial" |
|  | "Bisphosphonate" AND "Osteoporosis" AND “GIO” AND "3 point bending" AND "Human" AND "Clinical trial" |
|  | "Bisphosphonate" AND "Osteoporosis" AND “GIO” AND "Nanoindentation" AND "Human" AND “Clinical trial” |
|  | "Bisphosphonate" AND "Osteoporosis" AND “GIO” AND "Crystallinity" AND "Human" AND “Clinical trial” |
|  | "Bisphosphonate"AND "Osteoporosis" AND “GIO” AND "Advanced glycation end products (AGEs)" AND "Human" |
|  | "Bisphosphonate" AND "Osteoporosis" AND “GIO” AND "Bone loss" AND "Human" AND “Clinical trial” |
|  | "Bisphosphonate" AND "osteoporosis" AND “GIO” AND "Enzymatic to non-enzymatic cross-linking ratio" AND "Human" |
|  | "Denosumab" AND "Osteoporosis" AND “GIO”AND "carbonate to phosphate ratio" AND "Human" |
|  | "Denosumab" AND "Osteoporosis" AND “GIO” AND "carbonate to amide ratio" AND "Human" |
|  | "Denosumab" AND "Osteoporosis" AND “GIO” AND "Mineral matrix ratio" AND "Human" |
|  | "Denosumab" AND “GIO”AND "Osteoporosis" AND "Collagen maturity" AND "Human" |
|  | "Denosumab" AND "Osteoporosis" AND “GIO” AND "Microdamage accumulation" AND "Human" |
|  | "Denosumab" AND "Osteoporosis" AND “GIO” AND "Heterogeneity index" AND "Human" |
|  | "Denosumab" AND "Osteoporosis" AND “GIO” AND "Pentosidine" AND "Human" |
|  | "Denosumab" AND "Osteoporosis" AND “GIO” AND "Degree of mineralization" AND "Human" |
|  | "Denosumab" AND "Osteoporosis" AND “GIO”AND "Homocysteine" AND "Human" AND "Clinical trial" |
|  | "Denosumab" AND "Osteoporosis" AND “GIO”AND "FEA" AND "Human" AND "Clinical trial" |
|  | "Denosumab" AND "Osteoporosis" AND “GIO” AND "3 point bending" AND "Human" AND "Clinical trial" |
|  | "Denosumab" AND "Osteoporosis" AND “GIO” AND "Nanoindentation" AND "Human" AND “Clinical trial” |
|  | "Denosumab" AND "Osteoporosis" AND “GIO” AND "Crystallinity" AND "Human" AND “Clinical trial” |
|  | "Denosumab" AND "Osteoporosis" AND “GIO” AND "Advanced glycation end products (AGEs)" AND "Human" |
|  | "Denosumab" AND "Osteoporosis" AND “GIO” AND "Bone loss" AND "Human" AND “Clinical trial” |
|  | "Denosumab" AND "osteoporosis" AND “GIO” AND "Enzymatic to non-enzymatic cross-linking ratio" AND "Human" |
|  | "Teriparatide" AND "Osteoporosis" AND “GIO”AND "carbonate to phosphate ratio" AND "Human" |
|  | "Teriparatide" AND "Osteoporosis" AND “GIO” AND "carbonate to amide ratio" AND "Human" |
|  | "Teriparatide" AND "Osteoporosis" AND “GIO” AND "Mineral matrix ratio" AND "Human" |
|  | "Teriparatide" AND “GIO”AND "Osteoporosis" AND "Collagen maturity" AND "Human" |
|  | "Teriparatide" AND "Osteoporosis" AND “GIO” AND "Microdamage accumulation" AND "Human" |
|  | "Teriparatide" AND "Osteoporosis" AND “GIO” AND "Heterogeneity index" AND "Human" |
|  | "Teriparatide" AND "Osteoporosis" AND “GIO” AND "Pentosidine" AND "Human" |
|  | "Teriparatide" AND "Osteoporosis" AND “GIO” AND "Degree of mineralization" AND "Human" |
|  | "Teriparatide" AND "Osteoporosis" AND “GIO”AND "Homocysteine" AND "Human" AND "Clinical trial" |
|  | "Teriparatide" AND "Osteoporosis" AND “GIO”AND "FEA" AND "Human" AND "Clinical trial" |
|  | "Teriparatide" AND "Osteoporosis" AND “GIO” AND "3 point bending" AND "Human" AND "Clinical trial" |
|  | "Teriparatide" AND "Osteoporosis" AND “GIO” AND "Nanoindentation" AND "Human" AND “Clinical trial” |
|  | "Teriparatide" AND "Osteoporosis" AND “GIO” AND "Crystallinity" AND "Human" AND “Clinical trial” |
|  | "Teriparatide" AND "Osteoporosis" AND “GIO” AND "Advanced glycation end products (AGEs)" AND "Human" |
|  | "Teriparatide" AND "Osteoporosis" AND “GIO” AND "Bone loss" AND "Human" AND “Clinical trial” |
|  | "Teriparatide" AND "osteoporosis" AND “GIO” AND "Enzymatic to non-enzymatic cross-linking ratio" AND "Human" |
|  | "Abaloparatide" AND "Osteoporosis" AND “GIO”AND "carbonate to phosphate ratio" AND "Human" |
|  | "Abaloparatide" AND "Osteoporosis" AND “GIO” AND "carbonate to amide ratio" AND "Human" |
|  | "Abaloparatide" AND "Osteoporosis" AND “GIO” AND "Mineral matrix ratio" AND "Human" |
|  | "Abaloparatide" AND “GIO”AND "Osteoporosis" AND "Collagen maturity" AND "Human" |
|  | "Abaloparatide" AND "Osteoporosis" AND “GIO” AND "Microdamage accumulation" AND "Human" |
|  | "Abaloparatide" AND "Osteoporosis" AND “GIO” AND "Heterogeneity index" AND "Human" |
|  | "Abaloparatide" AND "Osteoporosis" AND “GIO” AND "Pentosidine" AND "Human" |
|  | "Abaloparatide" AND "Osteoporosis" AND “GIO” AND "Degree of mineralization" AND "Human" |
|  | "Abaloparatide" AND "Osteoporosis" AND “GIO”AND "Homocysteine" AND "Human" AND "Clinical trial" |
|  | "Abaloparatide" AND "Osteoporosis" AND “GIO”AND "FEA" AND "Human" AND "Clinical trial" |
|  | "Abaloparatide" AND "Osteoporosis" AND “GIO” AND "3 point bending" AND "Human" AND "Clinical trial" |
|  | "Abaloparatide" AND "Osteoporosis" AND “GIO” AND "Nanoindentation" AND "Human" AND “Clinical trial” |
|  | "Abaloparatide" AND "Osteoporosis" AND “GIO” AND "Crystallinity" AND "Human" AND “Clinical trial” |
|  | "Abaloparatide" AND "Osteoporosis" AND “GIO” AND "Advanced glycation end products (AGEs)" AND "Human" |
|  | "Abaloparatide" AND "Osteoporosis" AND “GIO” AND "Bone loss" AND "Human" AND “Clinical trial” |
|  | "Abaloparatide" AND "osteoporosis" AND “GIO” AND "Enzymatic to non-enzymatic cross-linking ratio" AND "Human" |
|  | "Raloxifene" AND "Osteoporosis" AND “GIO”AND "carbonate to phosphate ratio" AND "Human" |
|  | "Raloxifene" AND "Osteoporosis" AND “GIO” AND "carbonate to amide ratio" AND "Human" |
|  | "Raloxifene" AND "Osteoporosis" AND “GIO” AND "Mineral matrix ratio" AND "Human" |
|  | "Raloxifene" AND “GIO”AND "Osteoporosis" AND "Collagen maturity" AND "Human" |
|  | "Raloxifene" AND "Osteoporosis" AND “GIO” AND "Microdamage accumulation" AND "Human" |
|  | "Raloxifene" AND "Osteoporosis" AND “GIO” AND "Heterogeneity index" AND "Human" |
|  | "Raloxifene" AND "Osteoporosis" AND “GIO” AND "Pentosidine" AND "Human" |
|  | "Raloxifene" AND "Osteoporosis" AND “GIO” AND "Degree of mineralization" AND "Human" |
|  | "Raloxifene" AND "Osteoporosis" AND “GIO”AND "Homocysteine" AND "Human" AND "Clinical trial" |
|  | "Raloxifene" AND "Osteoporosis" AND “GIO”AND "FEA" AND "Human" AND "Clinical trial" |
|  | "Raloxifene" AND "Osteoporosis" AND “GIO” AND "3 point bending" AND "Human" AND "Clinical trial" |
|  | "Raloxifene" AND "Osteoporosis" AND “GIO” AND "Nanoindentation" AND "Human" AND “Clinical trial” |
|  | "Raloxifene" AND "Osteoporosis" AND “GIO” AND "Crystallinity" AND "Human" AND “Clinical trial” |
|  | "Raloxifene"AND "Osteoporosis" AND “GIO” AND "Advanced glycation end products (AGEs)" AND "Human" |
|  | "Raloxifene" AND "Osteoporosis" AND “GIO” AND "Bone loss" AND "Human" AND “Clinical trial” |
|  | "Raloxifene" AND "osteoporosis" AND “GIO” AND "Enzymatic to non-enzymatic cross-linking ratio" AND "Human" |
|  | "Romosozumab" AND "Osteoporosis" AND “GIO”AND "carbonate to phosphate ratio" AND "Human" |
|  | "Romosozumab" AND "Osteoporosis" AND “GIO” AND "carbonate to amide ratio" AND "Human" |
|  | "Romosozumab" AND "Osteoporosis" AND “GIO” AND “Mineral matrix ratio" AND "Human" |
|  | "Romosozumab" AND “GIO”AND "Osteoporosis" AND "Collagen maturity" AND "Human" |
|  | "Romosozumab" AND "Osteoporosis" AND “GIO” AND "Microdamage accumulation" AND "Human" |
|  | "Romosozumab" AND "Osteoporosis" AND “GIO” AND "Heterogeneity index" AND "Human" |
|  | "Romosozumab" AND "Osteoporosis" AND “GIO” AND "Pentosidine" AND "Human" |
|  | "Romosozumab" AND "Osteoporosis" AND “GIO” AND "Degree of mineralization" AND "Human" |
|  | "Romosozumab" AND "Osteoporosis" AND “GIO”AND "Homocysteine" AND "Human" AND "Clinical trial" |
|  | "Romosozumab" AND "Osteoporosis" AND “GIO”AND "FEA" AND "Human" AND "Clinical trial" |
|  | "Romosozumab" AND "Osteoporosis" AND “GIO” AND "3 point bending" AND "Human" AND "Clinical trial" |
|  | "Romosozumab" AND "Osteoporosis" AND “GIO” AND "Nanoindentation" AND "Human" AND “Clinical trial” |
|  | "Romosozumab" AND "Osteoporosis" AND “GIO” AND "Crystallinity" AND "Human" AND “Clinical trial” |
|  | "Romosozumab"AND "Osteoporosis" AND “GIO” AND "Advanced glycation end products (AGEs)" AND "Human" |
|  | "Romosozumab" AND "Osteoporosis" AND “GIO” AND "Bone loss" AND "Human" AND “Clinical trial” |
|  | "Romosozumab" AND "osteoporosis" AND “GIO” AND "Enzymatic to non-enzymatic cross-linking ratio" AND "Human" |
|  | "Strontium ranelate" AND "Osteoporosis" AND “GIO”AND "carbonate to phosphate ratio" AND "Human" |
|  | "Strontium ranelate" AND "Osteoporosis" AND “GIO” AND "carbonate to amide ratio" AND "Human" |
|  | "Strontium ranelate" AND "Osteoporosis" AND “GIO” AND "Mineral matrix ratio" AND "Human" |
|  | "Strontium ranelate" AND “GIO”AND "Osteoporosis" AND "Collagen maturity" AND "Human" |
|  | "Strontium ranelate" AND "Osteoporosis" AND “GIO” AND "Microdamage accumulation" AND "Human" |
|  | "Strontium ranelate" AND "Osteoporosis" AND “GIO” AND "Heterogeneity index" AND "Human" |
|  | "Strontium ranelate" AND "Osteoporosis" AND “GIO” AND "Pentosidine" AND "Human" |
|  | "Strontium ranelate" AND "Osteoporosis" AND “GIO” AND "Degree of mineralization" AND "Human" |
|  | "Strontium ranelate" AND "Osteoporosis" AND “GIO”AND "Homocysteine" AND "Human" AND "Clinical trial" |
|  | "Strontium ranelate" AND "Osteoporosis" AND “GIO”AND "FEA" AND "Human" AND "Clinical trial" |
|  | "Strontium ranelate" AND "Osteoporosis" AND “GIO” AND "3 point bending" AND "Human" AND "Clinical trial" |
|  | "Strontium ranelate" AND "Osteoporosis" AND “GIO” AND "Nanoindentation" AND "Human" AND “Clinical trial” |
|  | "Strontium ranelate" AND "Osteoporosis" AND “GIO” AND "Crystallinity" AND "Human" AND “Clinical trial” |
|  | "Strontium ranelate"AND "Osteoporosis" AND “GIO” AND "Advanced glycation end products (AGEs)" AND "Human" |
|  | "Strontium ranelate" AND "Osteoporosis" AND “GIO” AND "Bone loss" AND "Human" AND “Clinical trial” |
|  | "Strontium ranelate" AND "osteoporosis" AND “GIO” AND "Enzymatic to non-enzymatic cross-linking ratio" AND "Human" |
|  | "Bisphosphonate" AND "Osteoporosis" AND “Hypertension”AND "carbonate to phosphate ratio" AND "Human" |
|  | "Bisphosphonate" AND "Osteoporosis" AND “Hypertension” AND "carbonate to amide ratio" AND "Human" |
|  | "Bisphosphonate" AND "Osteoporosis" AND “Hypertension” AND "Mineral matrix ratio" AND "Human" |
|  | "Bisphosphonate" AND “Hypertension”AND "Osteoporosis" AND "Collagen maturity" AND "Human" |
|  | "Bisphosphonate" AND "Osteoporosis" AND “Hypertension” AND "Microdamage accumulation" AND "Human" |
|  | "Bisphosphonate" AND "Osteoporosis" AND “Hypertension” AND "Heterogeneity index" AND "Human" |
|  | "Bisphosphonate" AND "Osteoporosis" AND “Hypertension” AND "Pentosidine" AND "Human" |
|  | "Bisphosphonate" AND "Osteoporosis" AND “Hypertension” AND "Degree of mineralization" AND "Human" |
|  | "Bisphosphonate" AND "Osteoporosis" AND “Hypertension”AND "Homocysteine" AND "Human" AND "Clinical trial" |
|  | "Bisphosphonate" AND "Osteoporosis" AND “Hypertension”AND "FEA" AND "Human" AND "Clinical trial" |
|  | "Bisphosphonate" AND "Osteoporosis" AND “Hypertension” AND "3 point bending" AND "Human" AND "Clinical trial" |
|  | "Bisphosphonate" AND "Osteoporosis" AND “Hypertension” AND "Nanoindentation" AND "Human" AND “Clinical trial” |
|  | "Bisphosphonate" AND "Osteoporosis" AND “Hypertension” AND "Crystallinity" AND "Human" AND “Clinical trial” |
|  | "Bisphosphonate"AND "Osteoporosis" AND “Hypertension” AND "Advanced glycation end products (AGEs)" AND "Human" |
|  | "Bisphosphonate" AND "Osteoporosis" AND “Hypertension” AND "Bone loss" AND "Human" AND “Clinical trial” |
|  | "Bisphosphonate" AND "osteoporosis" AND “Hypertension” AND "Enzymatic to non-enzymatic cross-linking ratio" AND "Human" |
|  | "Denosumab" AND "Osteoporosis" AND “Hypertension”AND "carbonate to phosphate ratio" AND "Human" |
|  | "Denosumab" AND "Osteoporosis" AND “Hypertension” AND "carbonate to amide ratio" AND "Human" |
|  | "Denosumab" AND "Osteoporosis" AND “Hypertension” AND "Mineral matrix ratio" AND "Human" |
|  | "Denosumab" AND “Hypertension”AND "Osteoporosis" AND "Collagen maturity" AND "Human" |
|  | "Denosumab" AND "Osteoporosis" AND “Hypertension” AND "Microdamage accumulation" AND "Human" |
|  | "Denosumab" AND "Osteoporosis" AND “Hypertension” AND "Heterogeneity index" AND "Human" |
|  | "Denosumab" AND "Osteoporosis" AND “Hypertension” AND "Pentosidine" AND "Human" |
|  | "Denosumab" AND "Osteoporosis" AND “Hypertension” AND "Degree of mineralization" AND "Human" |
|  | "Denosumab" AND "Osteoporosis" AND “Hypertension”AND "Homocysteine" AND "Human" AND "Clinical trial" |
|  | "Denosumab" AND "Osteoporosis" AND “Hypertension”AND "FEA" AND "Human" AND "Clinical trial" |
|  | "Denosumab" AND "Osteoporosis" AND “Hypertension” AND "3 point bending" AND "Human" AND "Clinical trial" |
|  | "Denosumab" AND "Osteoporosis" AND “Hypertension” AND "Nanoindentation" AND "Human" AND “Clinical trial” |
|  | "Denosumab" AND "Osteoporosis" AND “Hypertension” AND "Crystallinity" AND "Human" AND “Clinical trial” |
|  | "Denosumab"AND "Osteoporosis" AND “Hypertension” AND "Advanced glycation end products (AGEs)" AND "Human" |
|  | "Denosumab" AND "Osteoporosis" AND “Hypertension” AND "Bone loss" AND "Human" AND “Clinical trial” |
|  | "Denosumab" AND "osteoporosis" AND “Hypertension” AND "Enzymatic to non-enzymatic cross-linking ratio" AND "Human" |
|  | "Teriparatide" AND "Osteoporosis" AND “Hypertension”AND "carbonate to phosphate ratio" AND "Human" |
|  | "Teriparatide" AND "Osteoporosis" AND “Hypertension” AND "carbonate to amide ratio" AND "Human" |
|  | "Teriparatide" AND "Osteoporosis" AND “Hypertension” AND "Mineral matrix ratio" AND "Human" |
|  | "Teriparatide" AND “Hypertension”AND "Osteoporosis" AND "Collagen maturity" AND "Human" |
|  | "Teriparatide" AND "Osteoporosis" AND “Hypertension” AND "Microdamage accumulation" AND "Human" |
|  | "Teriparatide" AND "Osteoporosis" AND “Hypertension” AND "Heterogeneity index" AND "Human" |
|  | "Teriparatide" AND "Osteoporosis" AND “Hypertension” AND "Pentosidine" AND "Human" |
|  | "Teriparatide" AND "Osteoporosis" AND “Hypertension” AND "Degree of mineralization" AND "Human" |
|  | "Teriparatide" AND "Osteoporosis" AND “Hypertension”AND "Homocysteine" AND "Human" AND "Clinical trial" |
|  | "Teriparatide" AND "Osteoporosis" AND “Hypertension”AND "FEA" AND "Human" AND "Clinical trial" |
|  | "Teriparatide" AND "Osteoporosis" AND “Hypertension” AND "3 point bending" AND "Human" AND "Clinical trial" |
|  | "Teriparatide" AND "Osteoporosis" AND “Hypertension” AND "Nanoindentation" AND "Human" AND “Clinical trial” |
|  | "Teriparatide" AND "Osteoporosis" AND “Hypertension” AND "Crystallinity" AND "Human" AND “Clinical trial” |
|  | "Teriparatide"AND "Osteoporosis" AND “Hypertension” AND "Advanced glycation end products (AGEs)" AND "Human" |
|  | "Teriparatide" AND "Osteoporosis" AND “Hypertension” AND "Bone loss" AND "Human" AND “Clinical trial” |
|  | "Teriparatide" AND "osteoporosis" AND “Hypertension” AND "Enzymatic to non-enzymatic cross-linking ratio" AND "Human" |
|  | "Abaloparatide" AND "Osteoporosis" AND “Hypertension”AND "carbonate to phosphate ratio" AND "Human" |
|  | "Abaloparatide" AND "Osteoporosis" AND “Hypertension” AND "carbonate to amide ratio" AND "Human" |
|  | "Abaloparatide" AND "Osteoporosis" AND “Hypertension” AND "Mineral matrix ratio" AND "Human" |
|  | "Abaloparatide" AND “Hypertension”AND "Osteoporosis" AND "Collagen maturity" AND "Human" |
|  | "Abaloparatide" AND "Osteoporosis" AND “Hypertension” AND "Microdamage accumulation" AND "Human" |
|  | "Abaloparatide" AND "Osteoporosis" AND “Hypertension” AND "Heterogeneity index" AND "Human" |
|  | "Abaloparatide" AND "Osteoporosis" AND “Hypertension” AND "Pentosidine" AND "Human" |
|  | "Abaloparatide" AND "Osteoporosis" AND “Hypertension” AND "Degree of mineralization" AND "Human" |
|  | "Abaloparatide" AND "Osteoporosis" AND “Hypertension”AND "Homocysteine" AND "Human" AND "Clinical trial" |
|  | "Abaloparatide" AND "Osteoporosis" AND “Hypertension”AND "FEA" AND "Human" AND "Clinical trial" |
|  | "Abaloparatide" AND "Osteoporosis" AND “Hypertension” AND "3 point bending" AND "Human" AND "Clinical trial" |
|  | "Abaloparatide" AND "Osteoporosis" AND “Hypertension” AND "Nanoindentation" AND "Human" AND “Clinical trial” |
|  | "Abaloparatide" AND "Osteoporosis" AND “Hypertension” AND "Crystallinity" AND "Human" AND “Clinical trial” |
|  | "Abaloparatide"AND "Osteoporosis" AND “Hypertension” AND "Advanced glycation end products (AGEs)" AND "Human" |
|  | "Abaloparatide" AND "Osteoporosis" AND “Hypertension” AND "Bone loss" AND "Human" AND “Clinical trial” |
|  | "Abaloparatide" AND "osteoporosis" AND “Hypertension” AND "Enzymatic to non-enzymatic cross-linking ratio" AND "Human" |
|  | "Raloxifene" AND "Osteoporosis" AND “Hypertension”AND "carbonate to phosphate ratio" AND "Human" |
|  | "Raloxifene" AND "Osteoporosis" AND “Hypertension” AND "carbonate to amide ratio" AND "Human" |
|  | "Raloxifene" AND "Osteoporosis" AND “Hypertension” AND "Mineral matrix ratio" AND "Human" |
|  | "Raloxifene" AND “Hypertension”AND "Osteoporosis" AND "Collagen maturity" AND "Human" |
|  | "Raloxifene" AND "Osteoporosis" AND “Hypertension” AND "Microdamage accumulation" AND "Human" |
|  | "Raloxifene" AND "Osteoporosis" AND “Hypertension” AND "Heterogeneity index" AND "Human" |
|  | "Raloxifene" AND "Osteoporosis" AND “Hypertension” AND "Pentosidine" AND "Human" |
|  | "Raloxifene" AND "Osteoporosis" AND “Hypertension” AND "Degree of mineralization" AND "Human" |
|  | "Raloxifene" AND "Osteoporosis" AND “Hypertension”AND "Homocysteine" AND "Human" AND "Clinical trial" |
|  | "Raloxifene" AND "Osteoporosis" AND “Hypertension”AND "FEA" AND "Human" AND "Clinical trial" |
|  | "Raloxifene" AND "Osteoporosis" AND “Hypertension” AND "3 point bending" AND "Human" AND "Clinical trial" |
|  | "Raloxifene" AND "Osteoporosis" AND “Hypertension” AND "Nanoindentation" AND "Human" AND “Clinical trial” |
|  | "Raloxifene" AND "Osteoporosis" AND “Hypertension” AND "Crystallinity" AND "Human" AND “Clinical trial” |
|  | "Raloxifene"AND "Osteoporosis" AND “Hypertension” AND "Advanced glycation end products (AGEs)" AND "Human" |
|  | "Raloxifene" AND "Osteoporosis" AND “Hypertension” AND "Bone loss" AND "Human" AND “Clinical trial” |
|  | "Raloxifene" AND "osteoporosis" AND “Hypertension” AND "Enzymatic to non-enzymatic cross-linking ratio" AND "Human" |
|  | "Romosozumab" AND "Osteoporosis" AND “Hypertension”AND "carbonate to phosphate ratio" AND "Human" |
|  | "Romosozumab" AND "Osteoporosis" AND “Hypertension” AND "carbonate to amide ratio" AND "Human" |
|  | "Romosozumab" AND "Osteoporosis" AND “Hypertension” AND "Mineral matrix ratio" AND "Human" |
|  | "Romosozumab" AND “Hypertension”AND "Osteoporosis" AND "Collagen maturity" AND "Human" |
|  | "Romosozumab" AND "Osteoporosis" AND “Hypertension” AND "Microdamage accumulation" AND "Human" |
|  | "Romosozumab" AND "Osteoporosis" AND “Hypertension” AND "Heterogeneity index" AND "Human" |
|  | "Romosozumab" AND "Osteoporosis" AND “Hypertension” AND "Pentosidine" AND "Human" |
|  | "Romosozumab" AND "Osteoporosis" AND “Hypertension” AND "Degree of mineralization" AND "Human" |
|  | "Romosozumab" AND "Osteoporosis" AND “Hypertension”AND "Homo cysteine" AND "Human" AND "Clinical trial" |
|  | "Romosozumab" AND "Osteoporosis" AND “Hypertension”AND "FEA" AND "Human" AND "Clinical trial" |
|  | "Romosozumab" AND "Osteoporosis" AND “Hypertension” AND "3 point bending" AND "Human" AND "Clinical trial" |
|  | "Romosozumab" AND "Osteoporosis" AND “Hypertension” AND "Nanoindentation" AND "Human" AND “Clinical trial” |
|  | "Romosozumab" AND "Osteoporosis" AND “Hypertension” AND "Crystallinity" AND "Human" AND “Clinical trial” |
|  | "Romosozumab"AND "Osteoporosis" AND “Hypertension” AND "Advanced glycation end products (AGEs)" AND "Human" |
|  | "Romosozumab" AND "Osteoporosis" AND “Hypertension” AND "Bone loss" AND "Human" AND “Clinical trial” |
|  | "Romosozumab" AND "osteoporosis" AND “Hypertension” AND "Enzymatic to non-enzymatic cross-linking ratio" AND "Human" |
|  | "Strontium ranelate" AND "Osteoporosis" AND “Hypertension”AND "carbonate to phosphate ratio" AND "Human" |
|  | "Strontium ranelate" AND "Osteoporosis" AND “Hypertension” AND "carbonate to amide ratio" AND "Human" |
|  | "Strontium ranelate" AND "Osteoporosis" AND “Hypertension” AND "Mineral matrix ratio" AND "Human" |
|  | "Strontium ranelate" AND “Hypertension”AND "Osteoporosis" AND "Collagen maturity" AND "Human" |
|  | "Strontium ranelate" AND "Osteoporosis" AND “Hypertension” AND "Microdamage accumulation" AND "Human" |
|  | "Strontium ranelate" AND "Osteoporosis" AND “Hypertension” AND "Heterogeneity index" AND "Human" |
|  | "Strontium ranelate" AND "Osteoporosis" AND “Hypertension” AND "Pentosidine" AND "Human" |
|  | "Strontium ranelate" AND "Osteoporosis" AND “Hypertension” AND "Degree of mineralization" AND "Human" |
|  | "Strontium ranelate" AND "Osteoporosis" AND “Hypertension”AND "Homocysteine" AND "Human" AND "Clinical trial" |
|  | "Strontium ranelate" AND "Osteoporosis" AND “Hypertension”AND "FEA" AND "Human" AND "Clinical trial" |
|  | "Strontium ranelate" AND "Osteoporosis" AND “Hypertension” AND "3 point bending" AND "Human" AND "Clinical trial" |
|  | "Strontium ranelate" AND "Osteoporosis" AND “Hypertension” AND "Nanoindentation" AND "Human" AND “Clinical trial” |
|  | "Strontium ranelate" AND "Osteoporosis" AND “Hypertension” AND "Bone loss" AND "Human" AND “Clinical trial” |
|  | "Strontium ranelate" AND "osteoporosis" AND “Hypertension” AND "Enzymatic to non-enzymatic cross-linking ratio" AND "Human" |
|  | "Bisphosphonate" AND "Osteoporosis" AND “IBD”AND "carbonate to phosphate ratio" AND "Human" |
|  | "Bisphosphonate" AND "Osteoporosis" AND “IBD” AND "carbonate to amide ratio" AND "Human" |
|  | "Bisphosphonate" AND "Osteoporosis" AND “IBD” AND "Mineral matrix ratio" AND "Human" |
|  | "Bisphosphonate" AND “IBD”AND "Osteoporosis" AND "Collagen maturity" AND "Human" |
|  | "Bisphosphonate" AND "Osteoporosis" AND “IBD” AND "Microdamage accumulation" AND "Human" |
|  | "Bisphosphonate" AND "Osteoporosis" AND “IBD” AND "Heterogeneity index" AND "Human" |
|  | "Bisphosphonate" AND "Osteoporosis" AND “IBD” AND "Pentosidine" AND "Human" |
|  | "Bisphosphonate" AND "Osteoporosis" AND “IBD” AND "Degree of mineralization" AND "Human" |
|  | "Bisphosphonate" AND "Osteoporosis" AND “IBD”AND "Homocysteine" AND "Human" AND "Clinical trial" |
|  | "Bisphosphonate" AND "Osteoporosis" AND “IBD”AND "FEA" AND "Human" AND "Clinical trial" |
|  | "Bisphosphonate" AND "Osteoporosis" AND “IBD” AND "3 point bending" AND "Human" AND "Clinical trial" |
|  | "Bisphosphonate" AND "Osteoporosis" AND “IBD” AND "Nanoindentation" AND "Human" AND “Clinical trial” |
|  | "Bisphosphonate" AND "Osteoporosis" AND “IBD” AND "Crystallinity" AND "Human" AND “Clinical trial” |
|  | "Bisphosphonate"AND "Osteoporosis" AND “IBD” AND "Advanced glycation end products (AGEs)" AND "Human" |
|  | "Bisphosphonate" AND "Osteoporosis" AND “IBD” AND "Bone loss" AND "Human" AND “Clinical trial” |
|  | "Bisphosphonate" AND "osteoporosis" AND “IBD” AND "Enzymatic to non-enzymatic cross-linking ratio" AND "Human" |
|  | "Denosumab" AND "Osteoporosis" AND “IBD”AND "carbonate to phosphate ratio" AND "Human" |
|  | "Denosumab" AND "Osteoporosis" AND “IBD” AND "carbonate to amide ratio" AND "Human" |
|  | "Denosumab" AND "Osteoporosis" AND “IBD” AND "Mineral matrix ratio" AND "Human" |
|  | "Denosumab" AND “IBD”AND "Osteoporosis" AND "Collagen maturity" AND "Human" |
|  | "Denosumab" AND "Osteoporosis" AND “IBD” AND "Microdamage accumulation" AND "Human" |
|  | "Denosumab" AND "Osteoporosis" AND “IBD” AND "Heterogeneity index" AND "Human" |
|  | "Denosumab" AND "Osteoporosis" AND “IBD” AND "Pentosidine" AND "Human" |
|  | "Denosumab" AND "Osteoporosis" AND “IBD” AND "Degree of mineralization" AND "Human" |
|  | "Denosumab" AND "Osteoporosis" AND “IBD”AND "Homocysteine" AND "Human" AND "Clinical trial" |
|  | "Denosumab" AND "Osteoporosis" AND “IBD”AND "FEA" AND "Human" AND "Clinical trial" |
|  | "Denosumab" AND "Osteoporosis" AND “IBD” AND "3 point bending" AND "Human" AND "Clinical trial" |
|  | "Denosumab" AND "Osteoporosis" AND “IBD” AND "Nanoindentation" AND "Human" AND “Clinical trial” |
|  | "Denosumab" AND "Osteoporosis" AND “IBD” AND "Crystallinity" AND "Human" AND “Clinical trial” |
|  | "Denosumab" AND "Osteoporosis" AND “IBD” AND "Advanced glycation end products (AGEs)" AND "Human" |
|  | "Denosumab" AND "Osteoporosis" AND “IBD” AND "Bone loss" AND "Human" AND “Clinical trial” |
|  | "Denosumab" AND "osteoporosis" AND “IBD” AND "Enzymatic to non-enzymatic cross-linking ratio" AND "Human" |
|  | "Teriparatide" AND "Osteoporosis" AND “IBD”AND "carbonate to phosphate ratio" AND "Human" |
|  | "Teriparatide" AND "Osteoporosis" AND “IBD” AND "carbonate to amide ratio" AND "Human" |
|  | "Teriparatide" AND "Osteoporosis" AND “IBD” AND "Mineral matrix ratio" AND "Human" |
|  | "Teriparatide" AND “IBD”AND "Osteoporosis" AND "Collagen maturity" AND "Human" |
|  | "Teriparatide" AND "Osteoporosis" AND “IBD” AND "Microdamage accumulation" AND "Human" |
|  | "Teriparatide" AND "Osteoporosis" AND “IBD” AND "Heterogeneity index" AND "Human" |
|  | "Teriparatide" AND "Osteoporosis" AND “IBD” AND "Pentosidine" AND "Human" |
|  | "Teriparatide" AND "Osteoporosis" AND “IBD” AND "Degree of mineralization" AND "Human" |
|  | "Teriparatide" AND "Osteoporosis" AND “IBD”AND "Homocysteine" AND "Human" AND "Clinical trial" |
|  | "Teriparatide" AND "Osteoporosis" AND “IBD”AND "FEA" AND "Human" AND "Clinical trial" |
|  | "Teriparatide" AND "Osteoporosis" AND “IBD” AND "3 point bending" AND "Human" AND "Clinical trial" |
|  | "Teriparatide" AND "Osteoporosis" AND “IBD” AND "3 point bending" AND "Human" AND "Clinical trial" |
|  | "Teriparatide" AND "Osteoporosis" AND “IBD” AND "Crystallinity" AND "Human" AND “Clinical trial” |
|  | "Teriparatide"AND "Osteoporosis" AND “IBD” AND "Advanced glycation end products (AGEs)" AND "Human" |
|  | "Teriparatide" AND "Osteoporosis" AND “IBD” AND "Bone loss" AND "Human" AND “Clinical trial” |
|  | "Teriparatide" AND "osteoporosis" AND “IBD” AND "Enzymatic to non-enzymatic cross-linking ratio" AND "Human" |
|  | "Abaloparatide" AND "Osteoporosis" AND “IBD”AND "carbonate to phosphate ratio" AND "Human" |
|  | "Abaloparatide" AND "Osteoporosis" AND “IBD” AND "carbonate to amide ratio" AND "Human" |
|  | "Abaloparatide" AND "Osteoporosis" AND “IBD” AND "Mineral matrix ratio" AND "Human" |
|  | "Abaloparatide" AND “IBD”AND "Osteoporosis" AND "Collagen maturity" AND "Human" |
|  | "Abaloparatide" AND "Osteoporosis" AND “IBD” AND "Microdamage accumulation" AND "Human" |
|  | "Abaloparatide" AND "Osteoporosis" AND “IBD” AND "Heterogeneity index" AND "Human" |
|  | "Abaloparatide" AND "Osteoporosis" AND “IBD” AND "Pentosidine" AND "Human" |
|  | "Abaloparatide" AND "Osteoporosis" AND “IBD” AND "Degree of mineralization" AND "Human" |
|  | "Abaloparatide" AND "Osteoporosis" AND “IBD”AND "Homocysteine" AND "Human" AND "Clinical trial" |
|  | "Abaloparatide" AND "Osteoporosis" AND “IBD”AND "FEA" AND "Human" AND "Clinical trial" |
|  | "Abaloparatide" AND "Osteoporosis" AND “IBD” AND "3 point bending" AND "Human" AND "Clinical trial" |
|  | "Abaloparatide" AND "Osteoporosis" AND “IBD” AND "Nanoindentation" AND "Human" AND “Clinical trial” |
|  | "Abaloparatide" AND "Osteoporosis" AND “IBD” AND "Crystallinity" AND "Human" AND “Clinical trial” |
|  | "Abaloparatide"AND "Osteoporosis" AND “IBD” AND "Advanced glycation end products (AGEs)" AND "Human" |
|  | "Abaloparatide" AND "Osteoporosis" AND “IBD” AND "Bone loss" AND "Human" AND “Clinical trial” |
|  | "Abaloparatide" AND "osteoporosis" AND “IBD” AND "Enzymatic to non-enzymatic cross-linking ratio" AND "Human" |
|  | "Raloxifene" AND "Osteoporosis" AND “IBD”AND "carbonate to phosphate ratio" AND "Human" |
|  | "Raloxifene" AND "Osteoporosis" AND “IBD” AND "carbonate to amide ratio" AND "Human" |
|  | "Raloxifene" AND "Osteoporosis" AND “IBD” AND "Mineral matrix ratio" AND "Human" |
|  | "Raloxifene" AND “IBD”AND "Osteoporosis" AND "Collagen maturity" AND "Human" |
|  | "Raloxifene" AND "Osteoporosis" AND “IBD” AND "Microdamage accumulation" AND "Human" |
|  | "Raloxifene" AND "Osteoporosis" AND “IBD” AND "Heterogeneity index" AND "Human" |
|  | "Raloxifene" AND "Osteoporosis" AND “IBD” AND "Pentosidine" AND "Human" |
|  | "Raloxifene" AND "Osteoporosis" AND “IBD” AND "Degree of mineralization" AND "Human" |
|  | "Raloxifene" AND "Osteoporosis" AND “IBD”AND "Homocysteine" AND "Human" AND "Clinical trial" |
|  | "Raloxifene" AND "Osteoporosis" AND “IBD”AND "FEA" AND "Human" AND "Clinical trial" |
|  | "Raloxifene" AND "Osteoporosis" AND “IBD” AND "3 point bending" AND "Human" AND "Clinical trial" |
|  | "Raloxifene" AND "Osteoporosis" AND “IBD” AND "Nanoindentation" AND "Human" AND “Clinical trial” |
|  | "Raloxifene" AND "Osteoporosis" AND “IBD” AND "Crystallinity" AND "Human" AND “Clinical trial” |
|  | "Raloxifene"AND "Osteoporosis" AND “IBD” AND "Advanced glycation end products (AGEs)" AND "Human" |
|  | "Raloxifene" AND "Osteoporosis" AND “IBD” AND "Bone loss" AND "Human" AND “Clinical trial” |
|  | "Raloxifene" AND "osteoporosis" AND “IBD” AND "Enzymatic to non-enzymatic cross-linking ratio" AND "Human" |
|  | "Romosozumab" AND "Osteoporosis" AND “IBD”AND "carbonate to phosphate ratio" AND "Human" |
|  | "Romosozumab" AND "Osteoporosis" AND “IBD” AND "carbonate to amide ratio" AND "Human" |
|  | "Romosozumab" AND "Osteoporosis" AND “IBD” AND "Mineral matrix ratio" AND "Human" |
|  | "Romosozumab" AND “IBD”AND "Osteoporosis" AND "Collagen maturity" AND "Human" |
|  | "Romosozumab" AND "Osteoporosis" AND “IBD” AND "Microdamage accumulation" AND "Human" |
|  | "Romosozumab" AND "Osteoporosis" AND “IBD” AND "Heterogeneity index" AND "Human" |
|  | "Romosozumab" AND "Osteoporosis" AND “IBD” AND "Pentosidine" AND "Human" |
|  | "Romosozumab" AND "Osteoporosis" AND “IBD” AND "Degree of mineralization" AND "Human" |
|  | "Romosozumab" AND "Osteoporosis" AND “IBD” AND "Homo cysteine" AND "Human" AND "Clinical trial" |
|  | "Romosozumab" AND "Osteoporosis" AND “IBD” AND "FEA" AND "Human" AND "Clinical trial" |
|  | "Romosozumab" AND "Osteoporosis" AND “IBD” AND "3 point bending" AND "Human" AND "Clinical trial" |
|  | "Romosozumab" AND "Osteoporosis" AND “IBD” AND "Nanoindentation" AND "Human" AND “Clinical trial” |
|  | "Romosozumab" AND "Osteoporosis" AND “IBD” AND "Crystallinity" AND "Human" AND “Clinical trial” |
|  | "Romosozumab"AND "Osteoporosis" AND “IBD” AND "Advanced glycation end products (AGEs)" AND "Human" |
|  | "Romosozumab" AND "Osteoporosis" AND “IBD” AND "Bone loss" AND "Human" AND “Clinical trial” |
|  | "Romosozumab" AND "osteoporosis" AND “IBD” AND "Enzymatic to non-enzymatic cross-linking ratio" AND "Human" |
|  | "Strontium ranelate" AND "Osteoporosis" AND “IBD” AND "carbonate to phosphate ratio" AND "Human" |
|  | "Strontium ranelate" AND "Osteoporosis" AND “IBD” AND "carbonate to amide ratio" AND "Human" |
|  | "Strontium ranelate" AND "Osteoporosis" AND “IBD” AND "Mineral matrix ratio" AND "Human" |
|  | "Strontium ranelate" AND “IBD”AND "Osteoporosis" AND "Collagen maturity" AND "Human" |
|  | "Strontium ranelate" AND "Osteoporosis" AND “IBD” AND "Microdamage accumulation" AND "Human" |
|  | "Strontium ranelate" AND "Osteoporosis" AND “IBD” AND "Heterogeneity index" AND "Human" |
|  | "Strontium ranelate" AND "Osteoporosis" AND “IBD” AND "Pentosidine" AND "Human" |
|  | "Strontium ranelate" AND "Osteoporosis" AND “IBD” AND "Degree of mineralization" AND "Human" |
|  | "Strontium ranelate" AND "Osteoporosis" AND “IBD” AND "Homocysteine" AND "Human" AND "Clinical trial" |
|  | "Strontium ranelate" AND "Osteoporosis" AND “IBD” AND "FEA" AND "Human" AND "Clinical trial" |
|  | "Strontium ranelate" AND "Osteoporosis" AND “IBD” AND "3 point bending" AND "Human" AND "Clinical trial" |
|  | "Strontium ranelate" AND "Osteoporosis" AND “IBD” AND "Nanoindentation" AND "Human" AND “Clinical trial” |
|  | "Strontium ranelate" AND "Osteoporosis" AND “IBD” AND "Bone loss" AND "Human" AND “Clinical trial” |
|  | "Strontium ranelate" AND "osteoporosis" AND “IBD” AND "Enzymatic to non-enzymatic cross-linking ratio" AND "Human" |
|  | "Strontium ranelate" AND "Osteoporosis" AND “IBD” AND "Nanoindentation" AND "Human" AND “Clinical trial” |
|  | " Strontium ranelate " AND "Osteoporosis" AND “IBD” AND "Crystallinity" AND "Human" AND “Clinical trial” |
|  | "Bisphosphonate" AND "Osteoporosis" AND “DIABETES”AND "carbonate to phosphate ratio" AND "Human" |
|  | "Bisphosphonate" AND "Osteoporosis" AND “DIABETES” AND "carbonate to amide ratio" AND "Human" |
|  | "Bisphosphonate" AND "Osteoporosis" AND “DIABETES” AND "Mineral matrix ratio" AND "Human" |
|  | "Bisphosphonate" AND “DIABETES”AND "Osteoporosis" AND "Collagen maturity" AND "Human" |
|  | "Bisphosphonate" AND "Osteoporosis" AND “DIABETES” AND "Microdamage accumulation" AND "Human" |
|  | "Bisphosphonate" AND "Osteoporosis" AND “DIABETES” AND "Heterogeneity index" AND "Human" |
|  | "Bisphosphonate" AND "Osteoporosis" AND “DIABETES” AND "Pentosidine" AND "Human" |
|  | "Bisphosphonate" AND "Osteoporosis" AND “DIABETES” AND "Degree of mineralization" AND "Human" |
|  | "Bisphosphonate" AND "Osteoporosis" AND “DIABETES” AND "Homocysteine" AND "Human" AND "Clinical trial" |
|  | "Bisphosphonate" AND "Osteoporosis" AND “DIABETES”AND "FEA" AND "Human" AND "Clinical trial" |
|  | "Bisphosphonate" AND "Osteoporosis" AND “DIABETES” AND "3 point bending" AND "Human" AND "Clinical trial" |
|  | "Bisphosphonate" AND "Osteoporosis" AND “DIABETES” AND "Nanoindentation" AND "Human" AND “Clinical trial” |
|  | "Bisphosphonate" AND "Osteoporosis" AND “DIABETES” AND "Crystallinity" AND "Human" AND “Clinical trial” |
|  | "Bisphosphonate" AND "Osteoporosis" AND “DIABETES” AND "Advanced glycation end products (AGEs)" AND "Human" |
|  | "Bisphosphonate" AND "Osteoporosis" AND “DIABETES” AND "Bone loss" AND "Human" AND “Clinical trial” |
|  | "Bisphosphonate" AND "osteoporosis" AND “DIABETES” AND "Enzymatic to non-enzymatic cross-linking ratio" AND "Human" |
|  | "Denosumab" AND "Osteoporosis" AND “DIABETES”AND "carbonate to phosphate ratio" AND "Human" |
|  | "Denosumab" AND "Osteoporosis" AND “DIABETES” AND "carbonate to amide ratio" AND "Human" |
|  | "Denosumab" AND "Osteoporosis" AND “DIABETES” AND "Mineral matrix ratio" AND "Human" |
|  | "Denosumab" AND “DIABETES”AND "Osteoporosis" AND "Collagen maturity" AND "Human" |
|  | "Denosumab" AND "Osteoporosis" AND “DIABETES” AND "Microdamage accumulation" AND "Human" |
|  | "Denosumab" AND "Osteoporosis" AND “DIABETES” AND "Heterogeneity index" AND "Human" |
|  | "Denosumab" AND "Osteoporosis" AND “DIABETES” AND "Pentosidine" AND "Human" |
|  | "Denosumab" AND "Osteoporosis" AND “DIABETES” AND "Degree of mineralization" AND "Human" |
|  | "Denosumab" AND "Osteoporosis" AND “DIABETES”AND "Homocysteine" AND "Human" AND "Clinical trial" |
|  | "Denosumab" AND "Osteoporosis" AND “DIABETES”AND "FEA" AND "Human" AND "Clinical trial" |
|  | "Denosumab" AND "Osteoporosis" AND “DIABETES” AND "3 point bending" AND "Human" AND "Clinical trial" |
|  | "Denosumab" AND "Osteoporosis" AND “DIABETES” AND "Nanoindentation" AND "Human" AND “Clinical trial” |
|  | "Denosumab" AND "Osteoporosis" AND “DIABETES” AND "Crystallinity" AND "Human" AND “Clinical trial” |
|  | "Denosumab"AND "Osteoporosis" AND “DIABETES” AND "Advanced glycation end products (AGEs)" AND "Human" |
|  | "Denosumab" AND "Osteoporosis" AND “DIABETES” AND "Bone loss" AND "Human" AND “Clinical trial” |
|  | "Denosumab" AND "osteoporosis" AND “DIABETES” AND "Enzymatic to non-enzymatic cross-linking ratio" AND "Human" |
|  | "Teriparatide" AND "Osteoporosis" AND “DIABETES”AND "carbonate to phosphate ratio" AND "Human" |
|  | "Teriparatide" AND "Osteoporosis" AND “DIABETES” AND "carbonate to amide ratio" AND "Human" |
|  | "Teriparatide" AND "Osteoporosis" AND “DIABETES” AND "Mineral matrix ratio" AND "Human" |
|  | "Teriparatide" AND “DIABETES”AND "Osteoporosis" AND "Collagen maturity" AND "Human" |
|  | "Teriparatide" AND "Osteoporosis" AND “DIABETES” AND "Microdamage accumulation" AND "Human" |
|  | "Teriparatide" AND "Osteoporosis" AND “DIABETES” AND "Heterogeneity index" AND "Human" |
|  | "Teriparatide" AND "Osteoporosis" AND “DIABETES” AND "Pentosidine" AND "Human" |
|  | "Teriparatide" AND "Osteoporosis" AND “DIABETES” AND "Degree of mineralization" AND "Human" |
|  | "Teriparatide" AND "Osteoporosis" AND “DIABETES”AND "Homocysteine" AND "Human" AND "Clinical trial" |
|  | "Teriparatide" AND "Osteoporosis" AND “DIABETES”AND "FEA" AND "Human" AND "Clinical trial" |
|  | "Teriparatide" AND "Osteoporosis" AND “DIABETES” AND "3 point bending" AND "Human" AND "Clinical trial" |
|  | "Teriparatide" AND "Osteoporosis" AND “DIABETES” AND "Nanoindentation" AND "Human" AND “Clinical trial” |
|  | "Teriparatide" AND "Osteoporosis" AND “DIABETES” AND "Crystallinity" AND "Human" AND “Clinical trial” |
|  | "Teriparatide" AND "Osteoporosis" AND “DIABETES” AND "Advanced glycation end products (AGEs)" AND "Human" |
|  | "Teriparatide" AND "Osteoporosis" AND “DIABETES” AND "Bone loss" AND "Human" AND “Clinical trial” |
|  | "Teriparatide" AND "osteoporosis" AND “DIABETES” AND "Enzymatic to non-enzymatic cross-linking ratio" AND "Human" |
|  | "Abaloparatide" AND "Osteoporosis" AND “DIABETES”AND "carbonate to phosphate ratio" AND "Human" |
|  | "Abaloparatide" AND "Osteoporosis" AND “DIABETES” AND "carbonate to amide ratio" AND "Human" |
|  | "Abaloparatide" AND "Osteoporosis" AND “DIABETES” AND "Mineral matrix ratio" AND "Human" |
|  | "Abaloparatide" AND “DIABETES”AND "Osteoporosis" AND "Collagen maturity" AND "Human" |
|  | "Abaloparatide" AND "Osteoporosis" AND “DIABETES” AND "Microdamage accumulation" AND "Human" |
|  | "Abaloparatide" AND "Osteoporosis" AND “DIABETES” AND "Heterogeneity index" AND "Human" |
|  | "Abaloparatide" AND "Osteoporosis" AND “DIABETES” AND "Pentosidine" AND "Human" |
|  | "Abaloparatide" AND "Osteoporosis" AND “DIABETES” AND "Degree of mineralization" AND "Human" |
|  | "Abaloparatide" AND "Osteoporosis" AND “DIABETES”AND "Homocysteine" AND "Human" AND "Clinical trial" |
|  | "Abaloparatide" AND "Osteoporosis" AND “DIABETES”AND "FEA" AND "Human" AND "Clinical trial" |
|  | "Abaloparatide" AND "Osteoporosis" AND “DIABETES” AND "3 point bending" AND "Human" AND "Clinical trial" |
|  | "Abaloparatide" AND "Osteoporosis" AND “DIABETES” AND "Nanoindentation" AND "Human" AND “Clinical trial” |
|  | "Abaloparatide" AND "Osteoporosis" AND “DIABETES” AND "Crystallinity" AND "Human" AND “Clinical trial” |
|  | "Abaloparatide" AND "Osteoporosis" AND “DIABETES” AND "Advanced glycation end products (AGEs)" AND "Human" |
|  | "Abaloparatide" AND "Osteoporosis" AND “DIABETES” AND "Bone loss" AND "Human" AND “Clinical trial” |
|  | "Abaloparatide" AND "osteoporosis" AND “DIABETES” AND "Enzymatic to non-enzymatic cross-linking ratio" AND "Human" |
|  | "Raloxifene" AND "Osteoporosis" AND “DIABETES”AND "carbonate to phosphate ratio" AND "Human" |
|  | "Raloxifene" AND "Osteoporosis" AND “DIABETES” AND "carbonate to amide ratio" AND "Human" |
|  | "Raloxifene" AND "Osteoporosis" AND “DIABETES” AND "Mineral matrix ratio" AND "Human" |
|  | "Raloxifene" AND “DIABETES”AND "Osteoporosis" AND "Collagen maturity" AND "Human" |
|  | "Raloxifene" AND "Osteoporosis" AND “DIABETES” AND "Microdamage accumulation" AND "Human" |
|  | "Raloxifene" AND "Osteoporosis" AND “DIABETES” AND "Heterogeneity index" AND "Human" |
|  | "Raloxifene" AND "Osteoporosis" AND “DIABETES” AND "Pentosidine" AND "Human" |
|  | "Raloxifene" AND "Osteoporosis" AND “DIABETES” AND "Degree of mineralization" AND "Human" |
|  | "Raloxifene" AND "Osteoporosis" AND “DIABETES”AND "Homocysteine" AND "Human" AND "Clinical trial" |
|  | "Raloxifene" AND "Osteoporosis" AND “DIABETES”AND "FEA" AND "Human" AND "Clinical trial" |
|  | "Raloxifene" AND "Osteoporosis" AND “DIABETES” AND "3 point bending" AND "Human" AND "Clinical trial" |
|  | "Raloxifene" AND "Osteoporosis" AND “DIABETES” AND "Nanoindentation" AND "Human" AND “Clinical trial” |
|  | "Raloxifene" AND "Osteoporosis" AND “DIABETES” AND "Crystallinity" AND "Human" AND “Clinical trial” |
|  | "Raloxifene"AND "Osteoporosis" AND “DIABETES” AND "Advanced glycation end products (AGEs)" AND "Human" |
|  | "Raloxifene" AND "Osteoporosis" AND “DIABETES” AND "Bone loss" AND "Human" AND “Clinical trial” |
|  | "Raloxifene" AND "osteoporosis" AND “DIABETES” AND "Enzymatic to non-enzymatic cross-linking ratio" AND "Human" |
|  | "Romosozumab" AND "Osteoporosis" AND “DIABETES”AND "carbonate to phosphate ratio" AND "Human" |
|  | "Romosozumab" AND "Osteoporosis" AND “DIABETES” AND "carbonate to amide ratio" AND "Human" |
|  | "Romosozumab" AND "Osteoporosis" AND “DIABETES” AND "Mineral matrix ratio" AND "Human" |
|  | "Romosozumab" AND “DIABETES”AND "Osteoporosis" AND "Collagen maturity" AND "Human" |
|  | "Romosozumab" AND "Osteoporosis" AND “DIABETES” AND "Microdamage accumulation" AND "Human" |
|  | "Romosozumab" AND "Osteoporosis" AND “DIABETES” AND "Heterogeneity index" AND "Human" |
|  | "Romosozumab" AND "Osteoporosis" AND “DIABETES” AND "Pentosidine" AND "Human" |
|  | "Romosozumab" AND "Osteoporosis" AND “DIABETES” AND "Degree of mineralization" AND "Human" |
|  | "Romosozumab" AND "Osteoporosis" AND “DIABETES”AND "Homocysteine" AND "Human" AND "Clinical trial" |
|  | "Romosozumab" AND "Osteoporosis" AND “DIABETES”AND "FEA" AND "Human" AND "Clinical trial" |
|  | "Romosozumab" AND "Osteoporosis" AND “DIABETES” AND "3 point bending" AND "Human" AND "Clinical trial" |
|  | "Romosozumab" AND "Osteoporosis" AND “DIABETES” AND "Nanoindentation" AND "Human" AND “Clinical trial” |
|  | "Romosozumab" AND "Osteoporosis" AND “DIABETES” AND "Crystallinity" AND "Human" AND “Clinical trial” |
|  | "Romosozumab"AND "Osteoporosis" AND “DIABETES” AND "Advanced glycation end products (AGEs)" AND "Human" |
|  | "Romosozumab" AND "Osteoporosis" AND “DIABETES” AND "Bone loss" AND "Human" AND “Clinical trial” |
|  | "Romosozumab" AND "osteoporosis" AND “DIABETES” AND "Enzymatic to non-enzymatic cross-linking ratio" AND "Human" |
|  | "Strontium ranelate" AND "Osteoporosis" AND “DIABETES”AND "carbonate to phosphate ratio" AND "Human" |
|  | "Strontium ranelate" AND "Osteoporosis" AND “DIABETES” AND "carbonate to amide ratio" AND "Human" |
|  | "Strontium ranelate" AND "Osteoporosis" AND “DIABETES” AND "Mineral matrix ratio" AND "Human" |
|  | "Strontium ranelate" AND “DIABETES”AND "Osteoporosis" AND "Collagen maturity" AND "Human" |
|  | "Strontium ranelate" AND "Osteoporosis" AND “DIABETES” AND "Microdamage accumulation" AND "Human" |
|  | "Strontium ranelate" AND "Osteoporosis" AND “DIABETES” AND "Heterogeneity index" AND "Human" |
|  | "Strontium ranelate" AND "Osteoporosis" AND “DIABETES” AND "Pentosidine" AND "Human" |
|  | "Strontium ranelate" AND "Osteoporosis" AND “DIABETES” AND "Degree of mineralization" AND "Human" |
|  | "Strontium ranelate" AND "Osteoporosis" AND “DIABETES”AND "Homocysteine" AND "Human" AND "Clinical trial" |
|  | "Strontium ranelate" AND "Osteoporosis" AND “DIABETES”AND "FEA" AND "Human" AND "Clinical trial" |
|  | "Strontium ranelate" AND "Osteoporosis" AND “DIABETES” AND "3 point bending" AND "Human" AND "Clinical trial" |
|  | "Strontium ranelate" AND "Osteoporosis" AND “DIABETES” AND "Nanoindentation" AND "Human" AND “Clinical trial” |
|  | "Strontium ranelate" AND "Osteoporosis" AND “DIABETES” AND "Crystallinity" AND "Human" AND “Clinical trial” |
|  | "Strontium ranelate" AND "Osteoporosis" AND “DIABETES” AND "Advanced glycation end products (AGEs)" AND "Human" |
|  | "Strontium ranelate" AND "Osteoporosis" AND “DIABETES” AND "Bone loss" AND "Human" AND “Clinical trial” |
|  | "Strontium ranelate" AND "osteoporosis" AND “DIABETES” AND "Enzymatic to non-enzymatic cross-linking ratio" AND "Human" |

**Web of Science**

| Bisphosphonate and osteoporosis and CKD and Mineral matrix ratio |
| --- |
| Bisphosphonate and osteoporosis and CKD and carbonate to phosphate ratio |
| Bisphosphonate and osteoporosis and CKD and carbonate to amide ratio |
| Bisphosphonate and osteoporosis and CKD and Collagen maturity |
| Bisphosphonate and osteoporosis and CKD and enzymatic to non-enzymatic cross-linking |
| Bisphosphonate and osteoporosis and CKD and Pentosidine |
| Bisphosphonate and osteoporosis and CKD and Degree of mineralization |
| Bisphosphonate and osteoporosis and CKD and FEA |
| Bisphosphonate and osteoporosis and CKD and Nanoindentation |
| Bisphosphonate and osteoporosis and CKD and 3-point bending |
| Bisphosphonate and osteoporosis and CKD and Advance glycation end products |
| Bisphosphonate and osteoporosis and CKD and Microdamage accumulation |
| Bisphosphonate and osteoporosis and CKD and Bone loss. |
| Bisphosphonate and osteoporosis and CKD and homocysteine |
| Denosumab and osteoporosis and CKD and Heterogeneity index |
| Denosumab and osteoporosis and CKD and Mineral matrix ratio |
| Denosumab and osteoporosis and CKD and carbonate to phosphate ratio |
| Denosumab and osteoporosis and CKD and carbonate to amide ratio |
| Denosumab and osteoporosis and CKD and Collagen maturity |
| Denosumab and osteoporosis and CKD and enzymatic to non-enzymatic cross-linking |
| Denosumab and osteoporosis and CKD and Pentosidine |
| Denosumab and osteoporosis and CKD and Degree of mineralization |
| Denosumab and osteoporosis and CKD and FEA |
| Denosumab and osteoporosis and CKD and Nanoindentation |
| Denosumab and osteoporosis and CKD and 3-point bending |
| Denosumab and osteoporosis and CKD and Advance glycation end products |
| Denosumab and osteoporosis and CKD and Microdamage accumulation |
| Denosumab and osteoporosis and CKD and Bone loss. |
| Denosumab and osteoporosis and CKD and homocysteine |
| Denosumab and osteoporosis and CKD and Heterogeneity index |
| Teriparatide and osteoporosis and CKD and Mineral matrix ratio |
| Teriparatide and osteoporosis and CKD and carbonate to phosphate ratio |
| Teriparatide and osteoporosis and CKD and carbonate to amide ratio |
| Teriparatide and osteoporosis and CKD and Collagen maturity |
| Teriparatide and osteoporosis and CKD and enzymatic to non-enzymatic cross-linking |
| Teriparatide and osteoporosis and CKD and Pentosidine |
| Teriparatide and osteoporosis and CKD and Degree of mineralization |
| Teriparatide and osteoporosis and CKD and FEA |
| Teriparatide and osteoporosis and CKD and Nanoindentation |
| Teriparatide and osteoporosis and CKD and 3-point bending |
| Teriparatide and osteoporosis and CKD and Advance glycation end products |
| Teriparatide and osteoporosis and CKD and Microdamage accumulation |
| Teriparatide and osteoporosis and CKD and Bone loss. |
| Teriparatide and osteoporosis and CKD and homocysteine |
| Teriparatide and osteoporosis and CKD and Heterogeneity index |
| Abaloparatide and osteoporosis and CKD and Mineral matrix ratio |
| Abaloparatide and osteoporosis and CKD and carbonate to phosphate ratio |
| Abaloparatide and osteoporosis and CKD and carbonate to amide ratio |
| Abaloparatide and osteoporosis and CKD and Collagen maturity |
| Abaloparatide and osteoporosis and CKD and enzymatic to non-enzymatic cross-linking |
| Abaloparatide and osteoporosis and CKD and Pentosidine |
| Abaloparatide and osteoporosis and CKD and Degree of mineralization |
| Abaloparatide and osteoporosis and CKD and FEA |
| Abaloparatide and osteoporosis and CKD and Nanoindentation |
| Abaloparatide and osteoporosis and CKD and 3-point bending |
| Abaloparatide and osteoporosis and CKD and Advance glycation end products |
| Abaloparatide and osteoporosis and CKD and Microdamage accumulation |
| Abaloparatide and osteoporosis and CKD and Bone loss. |
| Abaloparatide and osteoporosis and CKD and homocysteine |
| Abaloparatide and osteoporosis and CKD and Heterogeneity index |
| Raloxifene and osteoporosis and CKD and Mineral matrix ratio |
| Raloxifene and osteoporosis and CKD and carbonate to phosphate ratio |
| Raloxifene and osteoporosis and CKD and carbonate to amide ratio |
| Raloxifene and osteoporosis and CKD and Collagen maturity |
| Raloxifene and osteoporosis and CKD and enzymatic to non-enzymatic cross-linking |
| Raloxifene and osteoporosis and CKD and Pentosidine |
| Raloxifene and osteoporosis and CKD and Degree of mineralization |
| Raloxifene and osteoporosis and CKD and FEA |
| Raloxifene and osteoporosis and CKD and Nanoindentation |
| Raloxifene and osteoporosis and CKD and 3-point bending |
| Raloxifene and osteoporosis and CKD and Advance glycation end products |
| Raloxifene and osteoporosis and CKD and Microdamage accumulation |
| Raloxifene and osteoporosis and CKD and Bone loss. |
| Raloxifene and osteoporosis and CKD and homocysteine |
| Raloxifene and osteoporosis and CKD and Heterogeneity index |
| Romosozumab and osteoporosis and CKD and Mineral matrix ratio |
| Romosozumab and osteoporosis and CKD and carbonate to phosphate ratio |
| Romosozumab and osteoporosis and CKD and carbonate to amide ratio |
| Romosozumab and osteoporosis and CKD and Collagen maturity |
| Romosozumab and osteoporosis and CKD and enzymatic to non-enzymatic cross-linking |
| Romosozumab and osteoporosis and CKD and Pentosidine |
| Romosozumab and osteoporosis and CKD and Degree of mineralization |
| Romosozumab and osteoporosis and CKD and FEA |
| Romosozumab and osteoporosis and CKD and Nanoindentation |
| Romosozumab and osteoporosis and CKD and 3-point bending |
| Romosozumab and osteoporosis and CKD and Advance glycation end products |
| Romosozumab and osteoporosis and CKD and Microdamage accumulation |
| Romosozumab and osteoporosis and CKD and Bone loss. |
| Romosozumab and osteoporosis and CKD and homocysteine |
| Romosozumab and osteoporosis and CKD and Heterogeneity index |
| Strontium ranelate and osteoporosis and CKD and Mineral matrix ratio |
| Strontium ranelate and osteoporosis and CKD and carbonate to phosphate ratio |
| Strontium ranelate and osteoporosis and CKD and carbonate to amide ratio |
| Strontium ranelate and osteoporosis and CKD and Collagen maturity |
| Strontium ranelate and osteoporosis and CKD and enzymatic to non-enzymatic cross-linking |
| Strontium ranelate and osteoporosis and CKD and Pentosidine |
| Strontium ranelate and osteoporosis and CKD and Degree of mineralization |
| Strontium ranelate and osteoporosis and CKD and FEA |
| Strontium ranelate and osteoporosis and CKD and Nanoindentation |
| Strontium ranelate and osteoporosis and CKD and 3-point bending |
| Strontium ranelate and osteoporosis and CKD and Advance glycation end products |
| Strontium ranelate and osteoporosis and CKD and Microdamage accumulation |
| Strontium ranelate and osteoporosis and CKD and Bone loss. |
| Strontium ranelate and osteoporosis and CKD and homocysteine |
| Strontium ranelate and osteoporosis and CKD and Heterogeneity index |
| Bisphosphonate and osteoporosis and IBD and Mineral matrix ratio |
| Bisphosphonate and osteoporosis and IBD and carbonate to phosphate ratio |
| Bisphosphonate and osteoporosis and IBD and carbonate to amide ratio |
| Bisphosphonate and osteoporosis and IBD and Collagen maturity |
| Bisphosphonate and osteoporosis and IBD and enzymatic to non-enzymatic cross-linking |
| Bisphosphonate and osteoporosis and IBD and Pentosidine |
| Bisphosphonate and osteoporosis and IBD and Degree of mineralization |
| Bisphosphonate and osteoporosis and IBD and FEA |
| Bisphosphonate and osteoporosis and IBD and Nanoindentation |
| Bisphosphonate and osteoporosis and IBD and 3-point bending |
| Bisphosphonate and osteoporosis and IBD and Advance glycation end products |
| Bisphosphonate and osteoporosis and IBD and Microdamage accumulation |
| Bisphosphonate and osteoporosis and IBD and Bone loss. |
| Bisphosphonate and osteoporosis and IBD and homocysteine |
| Denosumab and osteoporosis and IBD and Heterogeneity index |
| Denosumab and osteoporosis and IBD and Mineral matrix ratio |
| Denosumab and osteoporosis and IBD and carbonate to phosphate ratio |
| Denosumab and osteoporosis and IBD and carbonate to amide ratio |
| Denosumab and osteoporosis and IBD and Collagen maturity |
| Denosumab and osteoporosis and IBD and enzymatic to non-enzymatic cross-linking |
| Denosumab and osteoporosis and IBD and Pentosidine |
| Denosumab and osteoporosis and IBD and Degree of mineralization |
| Denosumab and osteoporosis and IBD and FEA |
| Denosumab and osteoporosis and IBD and Nanoindentation |
| Denosumab and osteoporosis and IBD and 3-point bending |
| Denosumab and osteoporosis and IBD and Advance glycation end products |
| Denosumab and osteoporosis and IBD and Microdamage accumulation |
| Denosumab and osteoporosis and IBD and Bone loss. |
| Denosumab and osteoporosis and IBD and homocysteine |
| Denosumab and osteoporosis and IBD and Heterogeneity index |
| Teriparatide and osteoporosis and IBD and Mineral matrix ratio |
| Teriparatide and osteoporosis and IBD and carbonate to phosphate ratio |
| Teriparatide and osteoporosis and IBD and carbonate to amide ratio |
| Teriparatide and osteoporosis and IBD and Collagen maturity |
| Teriparatide and osteoporosis and IBD and enzymatic to non-enzymatic cross-linking |
| Teriparatide and osteoporosis and IBD and Pentosidine |
| Teriparatide and osteoporosis and IBD and Degree of mineralization |
| Teriparatide and osteoporosis and IBD and FEA |
| Teriparatide and osteoporosis and IBD and Nanoindentation |
| Teriparatide and osteoporosis and IBD and 3-point bending |
| Teriparatide and osteoporosis and IBD and Advance glycation end products |
| Teriparatide and osteoporosis and IBD and Microdamage accumulation |
| Teriparatide and osteoporosis and IBD and Bone loss. |
| Teriparatide and osteoporosis and IBD and homocysteine |
| Teriparatide and osteoporosis and IBD and Heterogeneity index |
| Abaloparatide and osteoporosis and IBD and Mineral matrix ratio |
| Abaloparatide and osteoporosis and IBD and carbonate to phosphate ratio |
| Abaloparatide and osteoporosis and IBD and carbonate to amide ratio |
| Abaloparatide and osteoporosis and IBD and Collagen maturity |
| Abaloparatide and osteoporosis and IBD and enzymatic to non-enzymatic cross-linking |
| Abaloparatide and osteoporosis and IBD and Pentosidine |
| Abaloparatide and osteoporosis and IBD and Degree of mineralization |
| Abaloparatide and osteoporosis and IBD and FEA |
| Abaloparatide and osteoporosis and IBD and Nanoindentation |
| Abaloparatide and osteoporosis and IBD and 3-point bending |
| Abaloparatide and osteoporosis and IBD and Advance glycation end products |
| Abaloparatide and osteoporosis and IBD and Microdamage accumulation |
| Abaloparatide and osteoporosis and IBD and Bone loss. |
| Abaloparatide and osteoporosis and IBD and homocysteine |
| Abaloparatide and osteoporosis and IBD and Heterogeneity index |
| Raloxifene and osteoporosis and IBD and Mineral matrix ratio |
| Raloxifene and osteoporosis and IBD and carbonate to phosphate ratio |
| Raloxifene and osteoporosis and IBD and carbonate to amide ratio |
| Raloxifene and osteoporosis and IBD and Collagen maturity |
| Raloxifene and osteoporosis and IBD and enzymatic to non-enzymatic cross-linking |
| Raloxifene and osteoporosis and IBD and Pentosidine |
| Raloxifene and osteoporosis and IBD and Degree of mineralization |
| Raloxifene and osteoporosis and IBD and FEA |
| Raloxifene and osteoporosis and IBD and Nanoindentation |
| Raloxifene and osteoporosis and IBD and 3-point bending |
| Raloxifene and osteoporosis and IBD and Advance glycation end products |
| Raloxifene and osteoporosis and IBD and Microdamage accumulation |
| Raloxifene and osteoporosis and IBD and Bone loss. |
| Raloxifene and osteoporosis and IBD and homocysteine |
| Raloxifene and osteoporosis and IBD and Heterogeneity index |
| Romosozumab and osteoporosis and IBD and Mineral matrix ratio |
| Romosozumab and osteoporosis and IBD and carbonate to phosphate ratio |
| Romosozumab and osteoporosis and IBD and carbonate to amide ratio |
| Romosozumab and osteoporosis and IBD and Collagen maturity |
| Romosozumab and osteoporosis and IBD and enzymatic to non-enzymatic cross-linking |
| Romosozumab and osteoporosis and IBD and Pentosidine |
| Romosozumab and osteoporosis and IBD and Degree of mineralization |
| Romosozumab and osteoporosis and IBD and FEA |
| Romosozumab and osteoporosis and IBD and Nanoindentation |
| Romosozumab and osteoporosis and IBD and 3-point bending |
| Romosozumab and osteoporosis and IBD and Advance glycation end products |
| Romosozumab and osteoporosis and IBD and Microdamage accumulation |
| Romosozumab and osteoporosis and IBD and Bone loss. |
| Romosozumab and osteoporosis and IBD and homocysteine |
| Romosozumab and osteoporosis and IBD and Heterogeneity index |
| Strontium ranelate and osteoporosis and IBD and Mineral matrix ratio |
| Strontium ranelate and osteoporosis and IBD and carbonate to phosphate ratio |
| Strontium ranelate and osteoporosis and IBD and carbonate to amide ratio |
| Strontium ranelate and osteoporosis and IBD and Collagen maturity |
| Strontium ranelate and osteoporosis and IBD and enzymatic to non-enzymatic cross-linking |
| Strontium ranelate and osteoporosis and IBD and Pentosidine |
| Strontium ranelate and osteoporosis and IBD and Degree of mineralization |
| Strontium ranelate and osteoporosis and IBD and FEA |
| Strontium ranelate and osteoporosis and IBD and Nanoindentation |
| Strontium ranelate and osteoporosis and IBD and 3-point bending |
| Strontium ranelate and osteoporosis and IBD and Advance glycation end products |
| Strontium ranelate and osteoporosis and IBD and Microdamage accumulation |
| Strontium ranelate and osteoporosis and IBD and Bone loss. |
| Strontium ranelate and osteoporosis and IBD and homocysteine |
| Strontium ranelate and osteoporosis and IBD and Heterogeneity index |
| Bisphosphonate and osteoporosis and Hypertension and Mineral matrix ratio |
| Bisphosphonate and osteoporosis and Hypertension and carbonate to phosphate ratio |
| Bisphosphonate and osteoporosis and Hypertension and carbonate to amide ratio |
| Bisphosphonate and osteoporosis and Hypertension and Collagen maturity |
| Bisphosphonate and osteoporosis and Hypertension and enzymatic to non-enzymatic cross-linking |
| Bisphosphonate and osteoporosis and Hypertension and Pentosidine |
| Bisphosphonate and osteoporosis and Hypertension and Degree of mineralization |
| Bisphosphonate and osteoporosis and Hypertension and FEA |
| Bisphosphonate and osteoporosis and Hypertension and Nanoindentation |
| Bisphosphonate and osteoporosis and Hypertension and 3-point bending |
| Bisphosphonate and osteoporosis and Hypertension and Advance glycation end products |
| Bisphosphonate and osteoporosis and Hypertension and Microdamage accumulation |
| Bisphosphonate and osteoporosis and Hypertension and Bone loss. |
| Bisphosphonate and osteoporosis and Hypertension and homocysteine |
| Denosumab and osteoporosis and Hypertension and Heterogeneity index |
| Denosumab and osteoporosis and Hypertension and Mineral matrix ratio |
| Denosumab and osteoporosis and Hypertension and carbonate to phosphate ratio |
| Denosumab and osteoporosis and Hypertension and carbonate to amide ratio |
| Denosumab and osteoporosis and Hypertension and Collagen maturity |
| Denosumab and osteoporosis and Hypertension and enzymatic to non-enzymatic cross-linking |
| Denosumab and osteoporosis and Hypertension and Pentosidine |
| Denosumab and osteoporosis and Hypertension and Degree of mineralization |
| Denosumab and osteoporosis and Hypertension and FEA |
| Denosumab and osteoporosis and Hypertension and Nanoindentation |
| Denosumab and osteoporosis and Hypertension and 3-point bending |
| Denosumab and osteoporosis and Hypertension and Advance glycation end products |
| Denosumab and osteoporosis and Hypertension and Microdamage accumulation |
| Denosumab and osteoporosis and Hypertension and Bone loss. |
| Denosumab and osteoporosis and Hypertension and homocysteine |
| Teriparatide and osteoporosis and Hypertension and Mineral matrix ratio |
| Teriparatide and osteoporosis and Hypertension and carbonate to phosphate ratio |
| Teriparatide and osteoporosis and Hypertension and carbonate to amide ratio |
| Teriparatide and osteoporosis and Hypertension and Collagen maturity |
| Teriparatide and osteoporosis and Hypertension and enzymatic to non-enzymatic cross-linking |
| Teriparatide and osteoporosis and Hypertension and Pentosidine |
| Teriparatide and osteoporosis and Hypertension and Degree of mineralization |
| Teriparatide and osteoporosis and Hypertension and FEA |
| Teriparatide and osteoporosis and Hypertension and Nanoindentation |
| Teriparatide and osteoporosis and Hypertension and 3-point bending |
| Teriparatide and osteoporosis and Hypertension and Advance glycation end products |
| Teriparatide and osteoporosis and Hypertension and Microdamage accumulation |
| Teriparatide and osteoporosis and Hypertension and Bone loss. |
| Teriparatide and osteoporosis and Hypertension and homocysteine |
| Teriparatide and osteoporosis and Hypertension and Heterogeneity index |
| Abaloparatide and osteoporosis and Hypertension and Mineral matrix ratio |
| Abaloparatide and osteoporosis and Hypertension and carbonate to phosphate ratio |
| Abaloparatide and osteoporosis and Hypertension and carbonate to amide ratio |
| Abaloparatide and osteoporosis and Hypertension and Collagen maturity |
| Abaloparatide and osteoporosis and Hypertension and enzymatic to non-enzymatic cross-linking |
| Abaloparatide and osteoporosis and Hypertension and Pentosidine |
| Abaloparatide and osteoporosis and Hypertension and Degree of mineralization |
| Abaloparatide and osteoporosis and Hypertension and FEA |
| Abaloparatide and osteoporosis and Hypertension and Nanoindentation |
| Abaloparatide and osteoporosis and Hypertension and 3-point bending |
| Abaloparatide and osteoporosis and Hypertension and Advance glycation end products |
| Abaloparatide and osteoporosis and Hypertension and Microdamage accumulation |
| Abaloparatide and osteoporosis and Hypertension and Bone loss. |
| Abaloparatide and osteoporosis and Hypertension and homocysteine |
| Abaloparatide and osteoporosis and Hypertension and Heterogeneity index |
| Raloxifene and osteoporosis and Hypertension and Mineral matrix ratio |
| Raloxifene and osteoporosis and Hypertension and carbonate to phosphate ratio |
| Raloxifene and osteoporosis and Hypertension and carbonate to amide ratio |
| Raloxifene and osteoporosis and Hypertension and Collagen maturity |
| Raloxifene and osteoporosis and Hypertension and enzymatic to non-enzymatic cross-linking |
| Raloxifene and osteoporosis and Hypertension and Pentosidine |
| Raloxifene and osteoporosis and Hypertension and Degree of mineralization |
| Raloxifene and osteoporosis and Hypertension and FEA |
| Raloxifene and osteoporosis and Hypertension and Nanoindentation |
| Raloxifene and osteoporosis and Hypertension and 3-point bending |
| Raloxifene and osteoporosis and Hypertension and Advance glycation end products |
| Raloxifene and osteoporosis and Hypertension and Microdamage accumulation |
| Raloxifene and osteoporosis and Hypertension and Bone loss. |
| Raloxifene and osteoporosis and Hypertension and homocysteine |
| Raloxifene and osteoporosis and Hypertension and Heterogeneity index |
| Romosozumab and osteoporosis and Hypertension and Mineral matrix ratio |
| Romosozumab and osteoporosis and Hypertension and carbonate to phosphate ratio |
| Romosozumab and osteoporosis and Hypertension and carbonate to amide ratio |
| Romosozumab and osteoporosis and Hypertension and Collagen maturity |
| Romosozumab and osteoporosis and Hypertension and enzymatic to non-enzymatic cross-linking |
| Romosozumab and osteoporosis and Hypertension and Pentosidine |
| Romosozumab and osteoporosis and Hypertension and Degree of mineralization |
| Romosozumab and osteoporosis and Hypertension and FEA |
| Romosozumab and osteoporosis and Hypertension and Nanoindentation |
| Romosozumab and osteoporosis and Hypertension and 3-point bending |
| Romosozumab and osteoporosis and Hypertension and Advance glycation end products |
| Romosozumab and osteoporosis and Hypertension and Microdamage accumulation |
| Romosozumaband osteoporosis and Hypertension and Bone loss. |
| Romosozumab and osteoporosis and Hypertension and homocysteine |
| Romosozumab and osteoporosis and Hypertension and Heterogeneity index |
| Strontium ranelate and osteoporosis and Hypertension and Mineral matrix ratio |
| Strontium ranelate and osteoporosis and Hypertension and carbonate to phosphate ratio |
| Strontium ranelate and osteoporosis and Hypertension and carbonate to amide ratio |
| Strontium ranelate and osteoporosis and Hypertension and Collagen maturity |
| Strontium ranelate and osteoporosis and Hypertension and enzymatic to non-enzymatic cross-linking |
| Strontium ranelate and osteoporosis and Hypertension and Pentosidine |
| Strontium ranelate and osteoporosis and Hypertension and Degree of mineralization |
| Strontium ranelate and osteoporosis and Hypertension and FEA |
| Strontium ranelate and osteoporosis and Hypertension and Nanoindentation |
| Strontium ranelate and osteoporosis and Hypertension and 3-point bending |
| Strontium ranelate and osteoporosis and Hypertension and Advance glycation end products |
| Strontium ranelate and osteoporosis and Hypertension and Microdamage accumulation |
| Strontium ranelate and osteoporosis and Hypertension and Bone loss. |
| Strontium ranelate and osteoporosis and Hypertension and homocysteine |
| Strontium ranelate and osteoporosis and Hypertension and Heterogeneity index |
| Bisphosphonate and osteoporosis and GIO and Mineral matrix ratio |
| Bisphosphonate and osteoporosis and GIO and carbonate to phosphate ratio |
| Bisphosphonate and osteoporosis and GIO and carbonate to amide ratio |
| Bisphosphonate and osteoporosis and GIO and Collagen maturity |
| Bisphosphonate and osteoporosis and GIO and enzymatic to non-enzymatic cross-linking |
| Bisphosphonate and osteoporosis and GIO and Pentosidine |
| Bisphosphonate and osteoporosis and GIO and Degree of mineralization |
| Bisphosphonate and osteoporosis and GIO and FEA |
| Bisphosphonate and osteoporosis and GIO and Nanoindentation |
| Bisphosphonate and osteoporosis and GIO and 3-point bending |
| Bisphosphonate and osteoporosis and GIO and Advance glycation end products |
| Bisphosphonate and osteoporosis and GIO and Microdamage accumulation |
| Bisphosphonate and osteoporosis and GIO and Bone loss. |
| Bisphosphonate and osteoporosis and GIO and homocysteine |
| Denosumab and osteoporosis and GIO and Heterogeneity index |
| Denosumab and osteoporosis and GIO and Mineral matrix ratio |
| Denosumab and osteoporosis and GIO and carbonate to phosphate ratio |
| Denosumab and osteoporosis and GIO and carbonate to amide ratio |
| Denosumab and osteoporosis and GIO and Collagen maturity |
| Denosumab and osteoporosis and GIO and enzymatic to non-enzymatic cross-linking |
| Denosumab and osteoporosis and GIO and Pentosidine |
| Denosumab and osteoporosis and GIO and Degree of mineralization |
| Denosumab and osteoporosis and GIO and FEA |
| Denosumab and osteoporosis and GIO and Nanoindentation |
| Denosumab and osteoporosis and GIO and 3-point bending |
| Denosumab and osteoporosis and GIO and Advance glycation end products |
| Denosumab and osteoporosis and GIO and Microdamage accumulation |
| Denosumab and osteoporosis and GIO and Bone loss. |
| Denosumab and osteoporosis and GIO and homocysteine |
| Denosumab and osteoporosis and GIO and Heterogeneity index |
| Teriparatide and osteoporosis and GIO and Mineral matrix ratio |
| Teriparatide and osteoporosis and GIO and carbonate to phosphate ratio |
| Teriparatide and osteoporosis and GIO and carbonate to amide ratio |
| Teriparatide and osteoporosis and GIO and Collagen maturity |
| Teriparatide and osteoporosis and GIO and enzymatic to non-enzymatic cross-linking |
| Teriparatide and osteoporosis and GIO and Pentosidine |
| Teriparatide and osteoporosis and GIO and Degree of mineralization |
| Teriparatide and osteoporosis and GIO and FEA |
| Teriparatide and osteoporosis and GIO and Nanoindentation |
| Teriparatide and osteoporosis and GIO and 3-point bending |
| Teriparatide and osteoporosis and GIO and Advance glycation end products |
| Teriparatide and osteoporosis and GIO and Microdamage accumulation |
| Teriparatide and osteoporosis and GIO and Bone loss. |
| Teriparatide and osteoporosis and GIO and homocysteine |
| Teriparatide and osteoporosis and GIO and Heterogeneity index |
| Abaloparatide and osteoporosis and GIO and Mineral matrix ratio |
| Abaloparatide and osteoporosis and GIO and carbonate to phosphate ratio |
| Abaloparatide and osteoporosis and GIO and carbonate to amide ratio |
| Abaloparatide and osteoporosis and GIO and Collagen maturity |
| Abaloparatide and osteoporosis and GIO and enzymatic to non-enzymatic cross-linking |
| Abaloparatide and osteoporosis and GIO and Pentosidine |
| Abaloparatide and osteoporosis and GIO and Degree of mineralization |
| Abaloparatide and osteoporosis and GIO and FEA |
| Abaloparatide and osteoporosis and GIO and Nanoindentation |
| Abaloparatide and osteoporosis and GIO and 3-point bending |
| Abaloparatide and osteoporosis and GIO and Advance glycation end products |
| Abaloparatide and osteoporosis and GIO and Microdamage accumulation |
| Abaloparatide and osteoporosis and GIO and Bone loss. |
| Abaloparatide and osteoporosis and GIO and homocysteine |
| Abaloparatide and osteoporosis and GIO and Heterogeneity index |
| Raloxifene and osteoporosis and GIO and Mineral matrix ratio |
| Raloxifene and osteoporosis and GIO and carbonate to phosphate ratio |
| Raloxifene and osteoporosis and GIO and carbonate to amide ratio |
| Raloxifene and osteoporosis and GIO and Collagen maturity |
| Raloxifene and osteoporosis and GIO and enzymatic to non-enzymatic cross-linking |
| Raloxifene and osteoporosis and GIO and Pentosidine |
| Raloxifene and osteoporosis and GIO and Degree of mineralization |
| Raloxifene and osteoporosis and GIO and FEA |
| Raloxifene and osteoporosis and GIO and Nanoindentation |
| Raloxifene and osteoporosis and GIO and 3-point bending |
| Raloxifene and osteoporosis and GIO and Advance glycation end products |
| Raloxifene and osteoporosis and GIO and Microdamage accumulation |
| Raloxifene and osteoporosis and GIO and Bone loss. |
| Raloxifene and osteoporosis and GIO and homocysteine |
| Raloxifene and osteoporosis and GIO and Heterogeneity index |
| Romosozumab and osteoporosis and GIO and Mineral matrix ratio |
| Romosozumab and osteoporosis and GIO and carbonate to phosphate ratio |
| Romosozumab and osteoporosis and GIO and carbonate to amide ratio |
| Romosozumab and osteoporosis and GIO and Collagen maturity |
| Romosozumab and osteoporosis and GIO and enzymatic to non-enzymatic cross-linking |
| Romosozumab and osteoporosis and GIO and Pentosidine |
| Romosozumab and osteoporosis and GIO and Degree of mineralization |
| Romosozumab and osteoporosis and GIO and FEA |
| Romosozumab and osteoporosis and GIO and Nanoindentation |
| Romosozumab and osteoporosis and GIO and 3-point bending |
| Romosozumab and osteoporosis and GIO and Advance glycation end products |
| Romosozumab and osteoporosis and GIO and Microdamage accumulation |
| Romosozumab and osteoporosis and GIO and Bone loss. |
| Romosozumab and osteoporosis and GIO and homocysteine |
| Romosozumab and osteoporosis and GIO and Heterogeneity index |
| Strontium ranelate and osteoporosis and GIO and Mineral matrix ratio |
| Strontium ranelate and osteoporosis and GIO and carbonate to phosphate ratio |
| Strontium ranelate and osteoporosis and GIO and carbonate to amide ratio |
| Strontium ranelate and osteoporosis and GIO and Collagen maturity |
| Strontium ranelate and osteoporosis and GIO and enzymatic to non-enzymatic cross-linking |
| Strontium ranelate and osteoporosis and GIO and Pentosidine |
| Strontium ranelate and osteoporosis and GIO and Degree of mineralization |
| Strontium ranelate and osteoporosis and GIO and FEA |
| Strontium ranelate and osteoporosis and GIO and Nanoindentation |
| Strontium ranelate and osteoporosis and GIO and 3-point bending |
| Strontium ranelate and osteoporosis and GIO and Advance glycation end products |
| Strontium ranelate and osteoporosis and GIO and Microdamage accumulation |
| Strontium ranelate and osteoporosis and GIO and Bone loss. |
| Strontium ranelate and osteoporosis and GIO and homocysteine |
| Strontium ranelate and osteoporosis and GIO and Heterogeneity index |
| Bisphosphonate and osteoporosis and PMO and Mineral matrix ratio |
| Bisphosphonate and osteoporosis and PMO and carbonate to phosphate ratio |
| Bisphosphonate and osteoporosis and PMO and carbonate to amide ratio |
| Bisphosphonate and osteoporosis and PMO and Collagen maturity |
| Bisphosphonate and osteoporosis and PMO and enzymatic to non-enzymatic cross-linking |
| Bisphosphonate and osteoporosis and PMO and Pentosidine |
| Bisphosphonate and osteoporosis and PMO and Degree of mineralization |
| Bisphosphonate and osteoporosis and PMO and FEA |
| Bisphosphonate and osteoporosis and PMO and Nanoindentation |
| Bisphosphonate and osteoporosis and PMO and 3-point bending |
| Bisphosphonate and osteoporosis and PMO and Advance glycation end products |
| Bisphosphonate and osteoporosis and PMO and Microdamage accumulation |
| Bisphosphonate and osteoporosis and PMO and Bone loss. |
| Bisphosphonate and osteoporosis and PMO and homocysteine |
| Denosumab and osteoporosis and PMO and Heterogeneity index |
| Denosumab and osteoporosis and PMO and Mineral matrix ratio |
| Denosumab and osteoporosis and PMO and carbonate to phosphate ratio |
| Denosumab and osteoporosis and PMO and carbonate to amide ratio |
| Denosumab and osteoporosis and PMO and Collagen maturity |
| Denosumab and osteoporosis and PMO and enzymatic to non-enzymatic cross-linking |
| Denosumab and osteoporosis and PMO and Pentosidine |
| Denosumab and osteoporosis and PMO and Degree of mineralization |
| Denosumab and osteoporosis and PMO and FEA |
| Denosumab and osteoporosis and PMO and Nanoindentation |
| Denosumab and osteoporosis and PMO and 3-point bending |
| Denosumab and osteoporosis and PMO and Advance glycation end products |
| Denosumab and osteoporosis and PMO and Microdamage accumulation |
| Denosumab and osteoporosis and PMO and Bone loss. |
| Denosumab and osteoporosis and PMO and homocysteine |
| Denosumab and osteoporosis and PMO and Heterogeneity index |
| Teriparatide and osteoporosis and PMO and Mineral matrix ratio |
| Teriparatide and osteoporosis and PMO and carbonate to phosphate ratio |
| Teriparatide and osteoporosis and PMO and carbonate to amide ratio |
| Teriparatide and osteoporosis and PMO and Collagen maturity |
| Teriparatide and osteoporosis and PMO and enzymatic to non-enzymatic cross-linking |
| Teriparatide and osteoporosis and PMO and Pentosidine |
| Teriparatide and osteoporosis and PMO and Degree of mineralization |
| Teriparatide and osteoporosis and PMO and FEA |
| Teriparatide and osteoporosis and PMO and Nanoindentation |
| Teriparatide and osteoporosis and PMO and 3-point bending |
| Teriparatide and osteoporosis and PMO and Advance glycation end products |
| Teriparatide and osteoporosis and PMO and Microdamage accumulation |
| Teriparatide and osteoporosis and PMO and Bone loss. |
| Teriparatide and osteoporosis and PMO and homocysteine |
| Teriparatide and osteoporosis and PMO and Heterogeneity index |
| Abaloparatide and osteoporosis and PMO and Mineral matrix ratio |
| Abaloparatide and osteoporosis and PMO and carbonate to phosphate ratio |
| Abaloparatide and osteoporosis and PMO and carbonate to amide ratio |
| Abaloparatide and osteoporosis and PMO and Collagen maturity |
| Abaloparatide and osteoporosis and PMO and enzymatic to non-enzymatic cross-linking |
| Abaloparatide and osteoporosis and PMO and Pentosidine |
| Abaloparatide and osteoporosis and PMO and Degree of mineralization |
| Abaloparatide and osteoporosis and PMO and FEA |
| Abaloparatide and osteoporosis and PMO and Nanoindentation |
| Abaloparatide and osteoporosis and PMO and 3-point bending |
| Abaloparatide and osteoporosis and PMO and Advance glycation end products |
| Abaloparatide and osteoporosis and PMO and Microdamage accumulation |
| Abaloparatide and osteoporosis and PMO and Bone loss. |
| Abaloparatide and osteoporosis and PMO and homocysteine |
| Abaloparatide and osteoporosis and PMO and Heterogeneity index |
| Raloxifene and osteoporosis and PMO and Mineral matrix ratio |
| Raloxifene and osteoporosis and PMO and carbonate to phosphate ratio |
| Raloxifene and osteoporosis and PMO and carbonate to amide ratio |
| Raloxifene and osteoporosis and PMO and Collagen maturity |
| Raloxifene and osteoporosis and PMO and enzymatic to non-enzymatic cross-linking |
| Raloxifene and osteoporosis and PMO and Pentosidine |
| Raloxifene and osteoporosis and PMO and Degree of mineralization |
| Raloxifene and osteoporosis and PMO and FEA |
| Raloxifene and osteoporosis and PMO and Nanoindentation |
| Raloxifene and osteoporosis and PMO and 3-point bending |
| Raloxifene and osteoporosis and PMO and Advance glycation end products |
| Raloxifene and osteoporosis and PMO and Microdamage accumulation |
| Raloxifene and osteoporosis and PMO and Bone loss. |
| Raloxifene and osteoporosis and PMO and homocysteine |
| Raloxifene and osteoporosis and PMO and Heterogeneity index |
| Romosozumab and osteoporosis and PMO and Mineral matrix ratio |
| Romosozumab and osteoporosis and PMO and carbonate to phosphate ratio |
| Romosozumab and osteoporosis and PMO and carbonate to amide ratio |
| Romosozumab and osteoporosis and PMO and Collagen maturity |
| Romosozumab and osteoporosis and PMO and enzymatic to non-enzymatic cross-linking |
| Romosozumab and osteoporosis and PMO and Pentosidine |
| Romosozumab and osteoporosis and PMO and Degree of mineralization |
| Romosozumab and osteoporosis and PMO and FEA |
| Romosozumab and osteoporosis and PMO and Nanoindentation |
| Romosozumab and osteoporosis and PMO and 3-point bending |
| Romosozumab and osteoporosis and PMO and Advance glycation end products |
| Romosozumab and osteoporosis and PMO and Microdamage accumulation |
| Romosozumaband osteoporosis and PMO and Bone loss. |
| Romosozumab and osteoporosis and PMO and homocysteine |
| Romosozumab and osteoporosis and PMO and Heterogeneity index |
| Strontium ranelate and osteoporosis and PMO and Mineral matrix ratio |
| Strontium ranelate and osteoporosis and PMO and carbonate to phosphate ratio |
| Strontium ranelate and osteoporosis and PMO and carbonate to amide ratio |
| Strontium ranelate and osteoporosis and PMO and Collagen maturity |
| Strontium ranelate and osteoporosis and PMO and enzymatic to non-enzymatic cross-linking |
| Strontium ranelate and osteoporosis and PMO and Pentosidine |
| Strontium ranelate and osteoporosis and PMO and Degree of mineralization |
| Strontium ranelate and osteoporosis and PMO and FEA |
| Strontium ranelate and osteoporosis and PMO and Nanoindentation |
| Strontium ranelate and osteoporosis and PMO and 3-point bending |
| Strontium ranelate and osteoporosis and PMO and Advance glycation end products |
| Strontium ranelate and osteoporosis and PMO and Microdamage accumulation |
| Strontium ranelate and osteoporosis and PMO and Bone loss. |
| Strontium ranelate and osteoporosis and PMO and homocysteine |
| Strontium ranelate and osteoporosis and PMO and Heterogeneity index |
| Bisphosphonate and osteoporosis and Diabetes and Mineral matrix ratio |
| Bisphosphonate and osteoporosis and Diabetes and carbonate to phosphate ratio |
| Bisphosphonate and osteoporosis and Diabetes and carbonate to amide ratio |
| Bisphosphonate and osteoporosis and Diabetes and Collagen maturity |
| Bisphosphonate and osteoporosis and Diabetes and enzymatic to non-enzymatic cross-linking |
| Bisphosphonate and osteoporosis and Diabetes and Pentosidine |
| Bisphosphonate and osteoporosis and Diabetes and Degree of mineralization |
| Bisphosphonate and osteoporosis and Diabetes and FEA |
| Bisphosphonate and osteoporosis and Diabetes and Nanoindentation |
| Bisphosphonate and osteoporosis and Diabetes and 3-point bending |
| Bisphosphonate and osteoporosis and Diabetes and Advance glycation end products |
| Bisphosphonate and osteoporosis and Diabetes and Microdamage accumulation |
| Bisphosphonate and osteoporosis and Diabetes and Bone loss. |
| Bisphosphonate and osteoporosis and Diabetes and homocysteine |
| Denosumab and osteoporosis and Diabetes and Heterogeneity index |
| Denosumab and osteoporosis and Diabetes and Mineral matrix ratio |
| Denosumab and osteoporosis and Diabetes and carbonate to phosphate ratio |
| Denosumab and osteoporosis and Diabetes and carbonate to amide ratio |
| Denosumab and osteoporosis and Diabetes and Collagen maturity |
| Denosumab and osteoporosis and Diabetes and enzymatic to non-enzymatic cross-linking |
| Denosumab and osteoporosis and Diabetes and Pentosidine |
| Denosumab and osteoporosis and Diabetes and Degree of mineralization |
| Denosumab and osteoporosis and Diabetes and FEA |
| Denosumab and osteoporosis and Diabetes and Nanoindentation |
| Denosumab and osteoporosis and Diabetes and 3-point bending |
| Denosumab and osteoporosis and Diabetes and Advance glycation end products |
| Denosumab and osteoporosis and Diabetes and Microdamage accumulation |
| Denosumab and osteoporosis and Diabetes and Bone loss. |
| Denosumab and osteoporosis and Diabetes and homocysteine |
| Denosumab and osteoporosis and Diabetes and Heterogeneity index |
| Teriparatide and osteoporosis and Diabetes and Mineral matrix ratio |
| Teriparatide and osteoporosis and Diabetes and carbonate to phosphate ratio |
| Teriparatide and osteoporosis and Diabetes and carbonate to amide ratio |
| Teriparatide and osteoporosis and Diabetes and Collagen maturity |
| Teriparatide and osteoporosis and Diabetes and enzymatic to non-enzymatic cross-linking |
| Teriparatide and osteoporosis and Diabetes and Pentosidine |
| Teriparatide and osteoporosis and Diabetes and Degree of mineralization |
| Teriparatide and osteoporosis and Diabetes and FEA |
| Teriparatide and osteoporosis and Diabetes and Nanoindentation |
| Teriparatide and osteoporosis and Diabetes and 3-point bending |
| Teriparatide and osteoporosis and Diabetes and Advance glycation end products |
| Teriparatide and osteoporosis and Diabetes and Microdamage accumulation |
| Teriparatide and osteoporosis and Diabetes and Bone loss. |
| Teriparatide and osteoporosis and Diabetes and homocysteine |
| Teriparatide and osteoporosis and Diabetes and Heterogeneity index |
| Abaloparatide and osteoporosis and Diabetes and Mineral matrix ratio |
| Abaloparatide and osteoporosis and Diabetes and carbonate to phosphate ratio |
| Abaloparatide and osteoporosis and Diabetes and carbonate to amide ratio |
| Abaloparatide and osteoporosis and Diabetes and Collagen maturity |
| Abaloparatide and osteoporosis and Diabetes and enzymatic to non-enzymatic cross-linking |
| Abaloparatide and osteoporosis and Diabetes and Pentosidine |
| Abaloparatide and osteoporosis and Diabetes and Degree of mineralization |
| Abaloparatide and osteoporosis and Diabetes and FEA |
| Abaloparatide and osteoporosis and Diabetes and Nanoindentation |
| Abaloparatide and osteoporosis and Diabetes and 3-point bending |
| Abaloparatide and osteoporosis and Diabetes and Advance glycation end products |
| Abaloparatide and osteoporosis and Diabetes and Microdamage accumulation |
| Abaloparatide and osteoporosis and Diabetes and Bone loss. |
| Abaloparatide and osteoporosis and Diabetes and homocysteine |
| Abaloparatide and osteoporosis and Diabetes and Heterogeneity index |
| Raloxifene and osteoporosis and Diabetes and Mineral matrix ratio |
| Raloxifene and osteoporosis and Diabetes and carbonate to phosphate ratio |
| Raloxifene and osteoporosis and Diabetes and carbonate to amide ratio |
| Raloxifene and osteoporosis and Diabetes and Collagen maturity |
| Raloxifene and osteoporosis and Diabetes and enzymatic to non-enzymatic cross-linking |
| Raloxifene and osteoporosis and Diabetes and Pentosidine |
| Raloxifene and osteoporosis and Diabetes and Degree of mineralization |
| Raloxifene and osteoporosis and Diabetes and FEA |
| Raloxifene and osteoporosis and Diabetes and Nanoindentation |
| Raloxifene and osteoporosis and Diabetes and 3-point bending |
| Raloxifene and osteoporosis and Diabetes and Advance glycation end products |
| Raloxifene and osteoporosis and Diabetes and Microdamage accumulation |
| Raloxifene and osteoporosis and Diabetes and Bone loss. |
| Raloxofene and osteoporosis and Diabetes and homocysteine |
| Raloxifene and osteoporosis and Diabetes and Heterogeneity index |
| Romosozumab and osteoporosis and Diabetes and Mineral matrix ratio |
| Romosozumab and osteoporosis and Diabetes and carbonate to phosphate ratio |
| Romosozumab and osteoporosis and Diabetes and carbonate to amide ratio |
| Romosozumab and osteoporosis and Diabetes and Collagen maturity |
| Romosozumab and osteoporosis and Diabetes and enzymatic to non-enzymatic cross-linking |
| Romosozumab and osteoporosis and Diabetes and Pentosidine |
| Romosozumab and osteoporosis and Diabetes and Degree of mineralization |
| Romosozumab and osteoporosis and Diabetes and FEA |
| Romosozumab and osteoporosis and Diabetes and Nanoindentation |
| Romosozumab and osteoporosis and Diabetes and 3-point bending |
| Romosozumab and osteoporosis and Diabetes and Advance glycation end products |
| Romosozumab and osteoporosis and Diabetes and Microdamage accumulation |
| Romosozumaband osteoporosis and Diabetes and Bone loss. |
| Romosozumab and osteoporosis and Diabetes and homocysteine |
| Romosozumab and osteoporosis and Diabetes and Heterogeneity index |
| Strontium ranelate and osteoporosis and Diabetes and Mineral matrix ratio |
| Strontium ranelate and osteoporosis and Diabetes and carbonate to phosphate ratio |
| Strontium ranelate and osteoporosis and Diabetes and carbonate to amide ratio |
| Strontium ranelate and osteoporosis and Diabetes and Collagen maturity |
| Strontium ranelate and osteoporosis and Diabetes and enzymatic to non-enzymatic cross-linking |
| Strontium ranelate and osteoporosis and Diabetes and Pentosidine |
| Strontium ranelate and osteoporosis and Diabetes and Degree of mineralization |
| Strontium ranelate and osteoporosis and Diabetes and FEA |
| Strontium ranelate and osteoporosis and Diabetes and Nanoindentation |
| Strontium ranelate and osteoporosis and Diabetes and 3-point bending |
| Strontium ranelate and osteoporosis and Diabetes and Advance glycation end products |
| Strontium ranelate and osteoporosis and Diabetes and Microdamage accumulation |
| Strontium ranelate and osteoporosis and Diabetes and Bone loss. |
| Strontium ranelate and osteoporosis and Diabetes and homocysteine |
| Strontium ranelate and osteoporosis and Diabetes and Heterogeneity index |
| Bisphosphonate and osteoporosis and Arthritis and Mineral matrix ratio |
| Bisphosphonate and osteoporosis and Arthritis and carbonate to phosphate ratio |
| Bisphosphonate and osteoporosis and Arthritis and carbonate to amide ratio |
| Bisphosphonate and osteoporosis and Arthritis and Collagen maturity |
| Bisphosphonate and osteoporosis and Arthritis and enzymatic to non-enzymatic cross-linking |
| Bisphosphonate and osteoporosis and Arthritis and Pentosidine |
| Bisphosphonate and osteoporosis and Arthritis and Degree of mineralization |
| Bisphosphonate and osteoporosis and Arthritis and FEA |
| Bisphosphonate and osteoporosis and Arthritis and Nanoindentation |
| Bisphosphonate and osteoporosis and Arthritis and 3-point bending |
| Bisphosphonate and osteoporosis and Arthritis and Advance glycation end products |
| Bisphosphonate and osteoporosis and Arthritis and Microdamage accumulation |
| Bisphosphonate and osteoporosis and Arthritis and Bone loss. |
| Bisphosphonate and osteoporosis and Arthritis and homocysteine |
| Denosumab and osteoporosis and Arthritis and Heterogeneity index |
| Denosumab and osteoporosis and Arthritis and Mineral matrix ratio |
| Denosumab and osteoporosis and Arthritis and carbonate to phosphate ratio |
| Denosumab and osteoporosis and Arthritis and carbonate to amide ratio |
| Denosumab and osteoporosis and Arthritis and Collagen maturity |
| Denosumab and osteoporosis and Arthritis and enzymatic to non-enzymatic cross-linking |
| Denosumab and osteoporosis and Arthritis and Pentosidine |
| Denosumab and osteoporosis and Arthritis and Degree of mineralization |
| Denosumab and osteoporosis and Arthritis and FEA |
| Denosumab and osteoporosis and Arthritis and Nanoindentation |
| Denosumab and osteoporosis and Arthritis and 3-point bending |
| Denosumab and osteoporosis and Arthritis and Advance glycation end products |
| Denosumab and osteoporosis and Arthritis and Microdamage accumulation |
| Denosumab and osteoporosis and Arthritis and Bone loss. |
| Denosumab and osteoporosis and Arthritis and homocysteine |
| Denosumab and osteoporosis and Arthritis and Heterogeneity index |
| Teriparatide and osteoporosis and Arthritis and Mineral matrix ratio |
| Teriparatide and osteoporosis and Arthritis and carbonate to phosphate ratio |
| Teriparatide and osteoporosis and Arthritis and carbonate to amide ratio |
| Teriparatide and osteoporosis and Arthritis and Collagen maturity |
| Teriparatide and osteoporosis and Arthritis and enzymatic to non-enzymatic cross-linking |
| Teriparatide and osteoporosis and Arthritis and Pentosidine |
| Teriparatide and osteoporosis and Arthritis and Degree of mineralization |
| Teriparatide and osteoporosis and Arthritis and FEA |
| Teriparatide and osteoporosis and Arthritis and Nanoindentation |
| Teriparatide and osteoporosis and Arthritis and 3-point bending |
| Teriparatide and osteoporosis and Arthritis and Advance glycation end products |
| Teriparatide and osteoporosis and Arthritis and Microdamage accumulation |
| Teriparatide and osteoporosis and Arthritis and Bone loss. |
| Teriparatide and osteoporosis and Arthritis and homocysteine |
| Teriparatide and osteoporosis and Arthritis and Heterogeneity index |
| Abaloparatide and osteoporosis and Arthritis and Mineral matrix ratio |
| Abaloparatide and osteoporosis and Arthritis and carbonate to phosphate ratio |
| Abaloparatide and osteoporosis and Arthritis and carbonate to amide ratio |
| Abaloparatide and osteoporosis and Arthritis and Collagen maturity |
| Abaloparatide and osteoporosis and Arthritis and enzymatic to non-enzymatic cross-linking |
| Abaloparatide and osteoporosis and Arthritis and Pentosidine |
| Abaloparatide and osteoporosis and Arthritis and Degree of mineralization |
| Abaloparatide and osteoporosis and Arthritis and FEA |
| Abaloparatide and osteoporosis and Arthritis and Nanoindentation |
| Abaloparatide and osteoporosis and Arthritis and 3-point bending |
| Abaloparatide and osteoporosis and Arthritis and Advance glycation end products |
| Abaloparatide and osteoporosis and Arthritis and Microdamage accumulation |
| Abaloparatide and osteoporosis and Arthritis and Bone loss. |
| Abaloparatide and osteoporosis and Arthritis and homocysteine |
| Abaloparatide and osteoporosis and Arthritis and Heterogeneity index |
| Raloxifene and osteoporosis and Arthritis and Mineral matrix ratio |
| Raloxifene and osteoporosis and Arthritis and carbonate to phosphate ratio |
| Raloxifene and osteoporosis and Arthritis and carbonate to amide ratio |
| Raloxifene and osteoporosis and Arthritis and Collagen maturity |
| Raloxifene and osteoporosis and Arthritis and enzymatic to non-enzymatic cross-linking |
| Raloxifene and osteoporosis and Arthritis and Pentosidine |
| Raloxifene and osteoporosis and Arthritis and Degree of mineralization |
| Raloxifene and osteoporosis and Arthritis and FEA |
| Raloxifene and osteoporosis and Arthritis and Nanoindentation |
| Raloxifene and osteoporosis and Arthritis and 3-point bending |
| Raloxifene and osteoporosis and Arthritis and Advance glycation end products |
| Raloxifene and osteoporosis and Arthritis and Microdamage accumulation |
| Raloxifene and osteoporosis and Arthritis and Bone loss. |
| Raloxifene and osteoporosis and Arthritis and homocysteine |
| Raloxifene and osteoporosis and Arthritis and Heterogeneity index |
| Romosozumab and osteoporosis and Arthritis and Mineral matrix ratio |
| Romosozumab and osteoporosis and Arthritis and carbonate to phosphate ratio |
| Romosozumab and osteoporosis and Arthritis and carbonate to amide ratio |
| Romosozumab and osteoporosis and Arthritis and Collagen maturity |
| Romosozumab and osteoporosis and Arthritis and enzymatic to non-enzymatic cross-linking |
| Romosozumab and osteoporosis and Arthritis and Pentosidine |
| Romosozumab and osteoporosis and Arthritis and Degree of mineralization |
| Romosozumab and osteoporosis and Arthritis and FEA |
| Romosozumab and osteoporosis and Arthritis and Nanoindentation |
| Romosozumab and osteoporosis and Arthritis and 3-point bending |
| Romosozumab and osteoporosis and Arthritis and Advance glycation end products |
| Romosozumab and osteoporosis and Arthritis and Microdamage accumulation |
| Romosozumaband osteoporosis and Arthritis and Bone loss. |
| Romosozumab and osteoporosis and Arthritis and homocysteine |
| Romosozumab and osteoporosis and Arthritis and Heterogeneity index |
| Strontium ranelate and osteoporosis and Arthritis and Mineral matrix ratio |
| Strontium ranelate and osteoporosis and Arthritis and carbonate to phosphate ratio |
| Strontium ranelate and osteoporosis and Arthritis and carbonate to amide ratio |
| Strontium ranelate and osteoporosis and Arthritis and Collagen maturity |
| Strontium ranelate and osteoporosis and Arthritis and enzymatic to non-enzymatic cross-linking |
| Strontium ranelate and osteoporosis and Arthritis and Pentosidine |
| Strontium ranelate and osteoporosis and Arthritis and Degree of mineralization |
| Strontium ranelate and osteoporosis and Arthritis and FEA |
| Strontium ranelate and osteoporosis and Arthritis and Nanoindentation |
| Strontium ranelate and osteoporosis and Arthritis and 3-point bending |
| Strontium ranelate and osteoporosis and Arthritis and Advance glycation end products |
| Strontium ranelate and osteoporosis and Arthritis and Microdamage accumulation |
| Strontium ranelate and osteoporosis and Arthritis and Bone loss. |
| Strontium ranelate and osteoporosis and Arthritis and homocysteine |
